# Catechol Thioesters: Ligands for Hierarchically Formed Lithium‐Bridged Titanium(IV) Helicates and Helicate‐Based Switches

**DOI:** 10.1002/chem.201905212

**Published:** 2020-03-05

**Authors:** A. Carel N. Kwamen, Gilles S. de Macedo, Constanze Wiederhold, Iris M. Oppel, Markus Albrecht

**Affiliations:** ^1^ Institut für Organische Chemie RWTH Aachen University Landoltweg 1 52074 Aachen Germany; ^2^ Institut für Anorganische Chemie RWTH Aachen University Landoltweg 1 52074 Aachen Germany

**Keywords:** helicates, molecular switches, self-assembly, thermodynamics, titanium

## Abstract

The thioester moiety is introduced as a lithium binding unit for the hierarchical formation of titanium(IV) catecholate‐based lithium‐bridged helicates. In solution, the coordination compounds show a monomer–dimer equilibrium which —in comparison to the oxo esters— is significantly shifted towards the monomers. In addition, the influence of the thioester side chain on the dimerization behavior is investigated and an expansible/compressible molecular switch is synthesized. In the latter case expansion and compression are performed reversibly in methanol, whereas in DMSO spontaneous expansion occurs.

## Graphical abstract

**Titanium springs**: Titanium (IV) triscatechol thioester complexes form in the presence of lithium cations dinuclear helicates, showing a monomer–dimer equilibrium with dimerization tendencies lying between those of the corresponding ketones and oxoesters. Based on this principle, it is possible to obtain a switchable spring‐type helicate which shows some spontaneous expansion in [D_6_]DMSO.

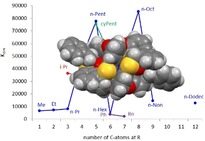

## Introduction

Thioesters are the „little brothers“ of oxoesters. In comparison to the oxygen homologous, they are more reactive regarding the cleavage of the C(=O)−S bond. The C=O double bond is less polarized. The high reactivity of the thioester functionality is important in synthesis as well as in nature for *trans*‐acylation reactions.^1^ As a prominent example, acetyl coenzyme A as an important synthetic building block in the biosynthesis of a huge number of natural products has to be mentioned.^2^

**Scheme 1:**
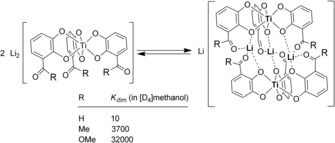
The monomer–dimer equilibrium which is observed for hierarchically assembled titanium(IV) triscatecholate helicates showing the dimerization constants of the aldehyde, the methylketone, and the methylester.

In 2005, we described the first examples of hierarchically formed helicates^3^ based on triple lithium‐bridged titanium(IV) triscatecholates with carbonyl moieties in 3‐position of the catechol ligand. In solution, the dimeric helicates are in equilibrium with the corresponding monomers. This equilibrium can be well observed by NMR spectroscopy and kinetic as well as thermodynamic parameters can be obtained for dimer association/dissociation. Hereby, the dimerization tendency strongly depends on the strength of lithium binding within the helicate. This mainly is influenced by the solvent, the charge of the monomeric unit, and by the donor ability of the carbonyl units. Scheme 1 shows that the poor aldehyde donor results in a lower dimerization constant compared to the better ketone donor and the much better ester donor.^4^ Additionally, inter‐substituent interactions as well as solvophobic/solvophilic effects do contribute to the dimer stabilization.^5^

In more recent studies, the lithium‐dependent dimerization of carbonyl substituted titanium(IV) triscatecholate complexes has been used as a molecular switch to control the selectivity of a Diels–Alder reaction^6^ or for the development of a helicate system which is able to expand or compress depending on some external stimuli showing some spring‐type behavior.^7^

In the present study a series of thioester‐substituted catechols has been synthesized and has been used to prepare the corresponding titanium(IV) complexes.^8^ It is expected that the thioesters show some distinct differences in the stability of the hierarchical helicates compared to the ester complexes. This is due to the lower polarization of C=O of the thioester and a corresponding weaker binding of the lithium cations in the dimer.^9^

In addition, an example for an expansible/compressible helicate based on an alkyl‐bridged bis(catechol thioester) is described and a special spontaneous expansion behavior is observed in [D_6_]DMSO.

## Results and Discussion

**Scheme 2:**
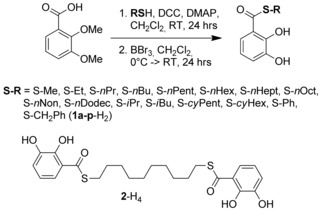
Preparation of the catechol thioesters **1 a**–**p‐**H_2_ and **2**‐H_4_.

Preparation of the catechol thioesters follows a modified protocol as described for the oxoesters (Scheme 2).^10^ Dimethoxybenzoic acid was transformed into the thioester by coupling of the corresponding thiol in the presence of DMAP (4‐*N*,*N*‐dimethylaminopyridine) and DCC (dicyclohexyl carbodiimide).^11^ In the second step the methyl ethers are cleaved by reaction with BBr_3_.^12^ The catechol thioester ligands **1**‐H_2_ are reacted with titanoyl bis(acetylacetonate) in the presence of lithium carbonate to obtain dinuclear triple lithium‐bridged titanium(IV) helicates Li[Li_3_(**1 a**–**p**)_6_Ti_2_].^4^

**Figure 1:**
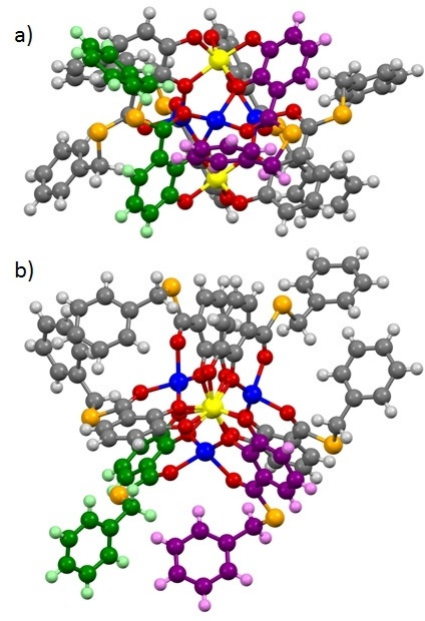
Side (a) and top view (b) of the molecular structure of the anion [Li_3_(**1 p**)_6_Ti_2_]^−^ in the crystal. C: black, H, white, O: red, S: orange, Li: blue, Ti, yellow. One ligand of the one monomer is shown in green, one of the other in purple.

X‐Ray quality crystals of the benzyl derivative Li[Li_3_(**1 p**)_6_Ti_2_] were obtained, the molecular structure of the anion [Li_3_(**1 p**)_6_Ti_2_]^−^ is shown in Figure 1.

The X‐ray structure analysis shows the dimeric central part of the hierarchically formed helicate with two titanium and three lithium cations (Ti⋅⋅⋅Ti=5.53, Ti⋅⋅⋅Li=3.38–3.49, and Li⋅⋅⋅Li=3.46–3.49 Å) very similar to the one observed for the corresponding aldehyde, ketone, or oxoester derivatives.^4^, ^5^ However, the C‐S‐C angle at the sulfur atom (97.8–100.8°) is smaller than observed for the corresponding oxoesters. In addition, the thioesters show short distances of the protons of the methylene group to the neighboring catechol unit (H⋅⋅⋅C_arom_=2.8–3.2 Å) contributing significantly to the stability of the dimer. This has been also observed for the oxygen homologues.^5^

**Figure 2:**
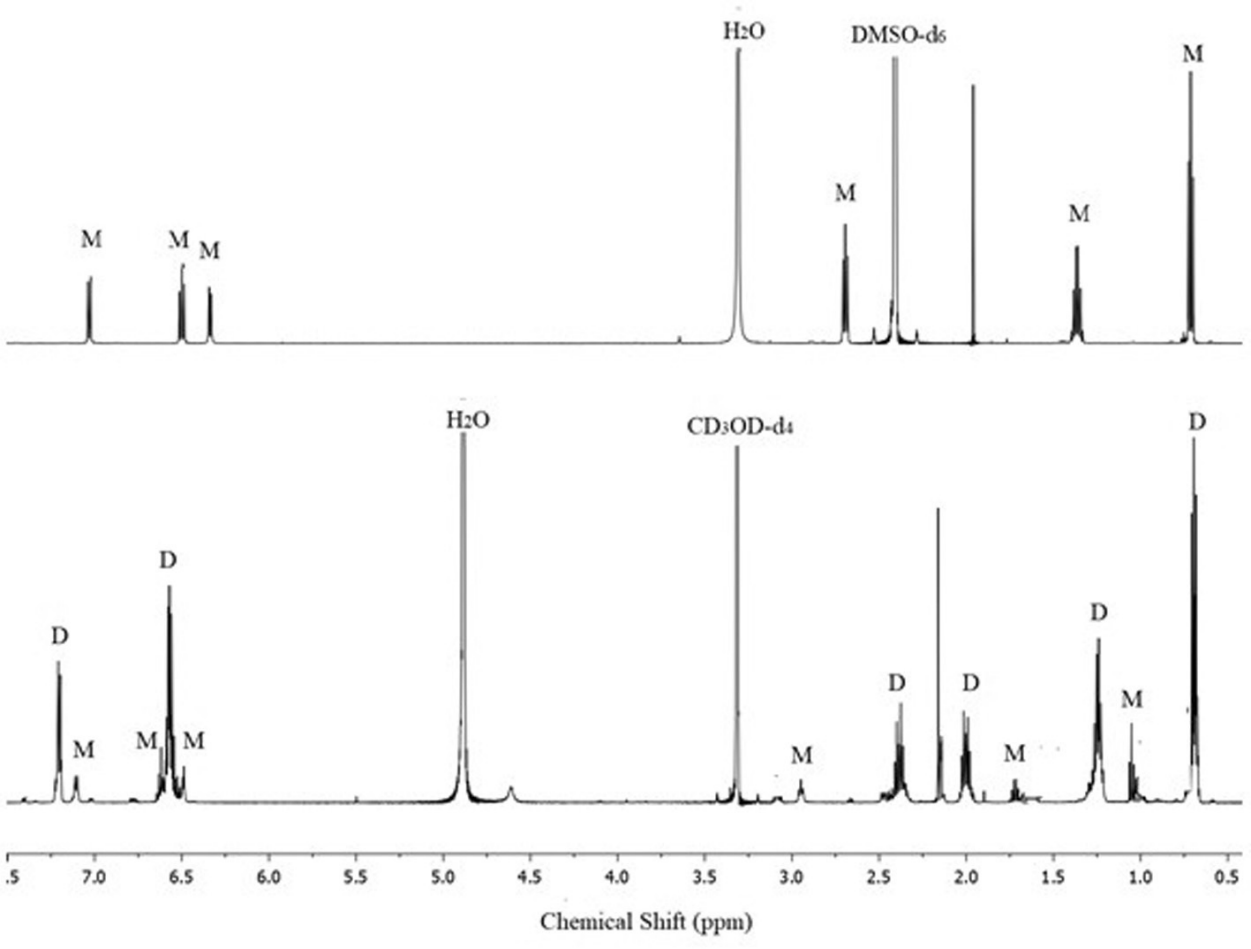
^1^H NMR spectra of the the catechol thioester complex Li[Li_3_(**1 c**)_6_Ti_2_]/[Li_2_(**1 c**)_3_Ti] in [D_6_]DMSO (a) showing only the monomeric species (M) and in [D_4_]MeOH (b) showing both monomer (M) and the dominating dimer (D).

**Figure 3:**
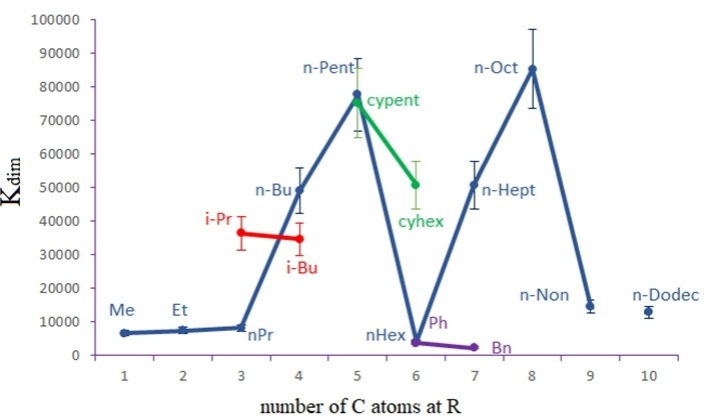
Dimerization constants *K*_dim_ [L mol^−1^] of Li[Li_3_(**1 a**–**p**)_6_Ti_2_] in [D_4_]MeOH. The ester substituent R is used to indicate the corresponding complex.

In [D_4_]MeOH solution both, the dimer as well as the monomer can be observed by ^1^H NMR spectroscopy (Figure 2). In [D_6_]DMSO, only peaks of the monomeric triscatecholate titanium(IV) complex are found due to the high lithium cation‐coordinating ability of the solvent. Removal of lithium cations from the dimer results in its full dissociation. Characteristic for the monomer is the observation of the CH_2_ group at sulfur as one single peak due to fast inversion of the stereochemistry at the complex unit. In the dimer, the stereochemistry at the complex is locked and diastereotopic protons are detected for SCH_2_.^13^ This is observed for the major species of Li[Li_3_(**1 c**)_6_Ti_2_]/[Li_2_(**1 c**)_3_Ti] in [D_4_]MeOH. The minor species is the monomer which does not show this diastereotopic behavior. At the room‐temperature equilibrium state of the monomer/dimer equilibrium in [D_4_]MeOH, the dimerization constant can be easily obtained from the NMR integration (see Table 1 and Figure 3, a concentration of 1 m is used as reference state in the determination of *K*_dim_).

**Table 1:** Dimerization constants of complexes Li[Li_3_(**1 a**–**p**)_6_Ti_2_] in [D_4_]MeOH at room temperature.

Complex	R	*K*_dim_		Complex	R	K_dim_
Li[Li_3_(**1 a**)_6_Ti_2_]	Me	6650±880		Li[Li_3_(**1 i**)_6_Ti_2_]	*n*‐Non	14600±1970
Li[Li_3_(**1 b**)_6_Ti_2_]	Et	7380±980		Li[Li_3_(**1 j**)_6_Ti_2_]	*n*‐Dodec	12870±1740
Li[Li_3_(**1 c**)_6_Ti_2_]	*n*‐Pr	8170±1090		Li[Li_3_(**1 k**)_6_Ti_2_]	*i*Pr	36440±5010
Li[Li_3_(**1 d**)_6_Ti_2_]	*n*‐Bu	49070±6760		Li[Li_3_(**1 l**)_6_Ti_2_]	*i*Bu	34670±4760
Li[Li_3_(**1 e**)_6_Ti_2_]	*n*‐Pent	77770±1080		Li[Li_3_(**1 m**)_6_Ti_2_]	*cy*Pent	75250±1040
Li[Li_3_(**1 f**)_6_Ti_2_]	*n*‐Hex	4020±520		Li[Li_3_(**1 n**)_6_Ti_2_]	*cy*Hex	50810±7010
Li[Li_3_(**1 g**)_6_Ti_2_]	*n*‐Hept	50820±7010		Li[Li_3_(**1 o**)_6_Ti_2_]	Ph	3790±490
Li[Li_3_(**1 h**)_6_Ti_2_]	*n*‐Oct	85370±1180		Li[Li_3_(**1 p**)_6_Ti_2_]	Bn	2230±280

Following remarkable aspects can be realized from the collection of dimerization constants of the thioester‐derived complexes:

1) In case of the methyl versus the ethyl oxoesters, the dimerization constant in [D_4_]MeOH of the corresponding dimeric coordination complexes raises dramatically from methyl (25 600) to ethyl (only dimer observed) due to stronger electron‐donating properties of the ethyl compared to the methyl substituent.^5^, ^14^ In case of the thioesters, the methyl (Li[Li_3_(**1 a**)_6_Ti_2_], *K*_dim_=6650), ethyl (Li[Li_3_(**1 b**)_6_Ti_2_], *K*_dim_=7380) and *n*‐propyl thioester dimers (Li[Li_3_(**1 c**)_6_Ti_2_], *K*_dim_=8170) are significantly lower and are only slightly increasing with the chain length. This shows a strong electronic isolation of the ester carbonyl and the alkyl substituent by the sulfur atom.

2) For the *n*Bu, *n*Pent, *n*Hept, and *n*Oct thioesters relatively high *K*_dim_ values are observed in [D_4_]MeOH, whereas the hexyl thioester results in a dramatic decrease. This drop has been also observed for the corresponding hexyl oxoester in [D_6_]DMSO,^5^ although it has not been as dramatic as with the thioester. This effect is assigned to a special conformation of the *n*‐hexyl chain.

3) For *n*Non and *n*Dodec thioesters, low dimer stability is observed. A similar effect has been observed for the oxoesters and there has been attributed to a loss of entropy due to side‐chain aggregation.^5^ The aggregation of the chains occurs due to solvophobicity of the alkyl groups. Chain aggregation minimizes the contact area with the solvent.

4) The branched *i*Pr and *i*Bu, as well as the cyclic *cy*Pent and *cy*Hex, thioester derivatives show relatively high dimerization constants which may be due to some dispersive interactions^15^ between the side chains in addition to solvophobic effects.

5) Aromatic units (Ph, Bn) show high solphophilicity in [D_4_]MeOH and thus *K*_dim_ is low.

The observed lithium‐dependent dimerization of the thioester‐derived triscatechol titanium(IV) complexes show some behavior as observed for the oxoesters^5^ but also some different distinct features which can be attributed to the sulfur atom of the thioester moiety.

In addition to the hierarchical helicates, a classical helicate Li[Li_3_(**2**)_3_Ti_2_] has been prepared by tethering two thioesters by a decyl‐bridge. Coordination of three internal lithium cations results in a compressed structure. Corresponding complexes with oxoesters have been earlier prepared and show the ability to be switched from the compressed to the expanded state or vice versa by simply removing or adding lithium cations. With the oxoesters the switching proceeds smoothly in [D_6_]DMSO solution.^7^

**Figure 4:**
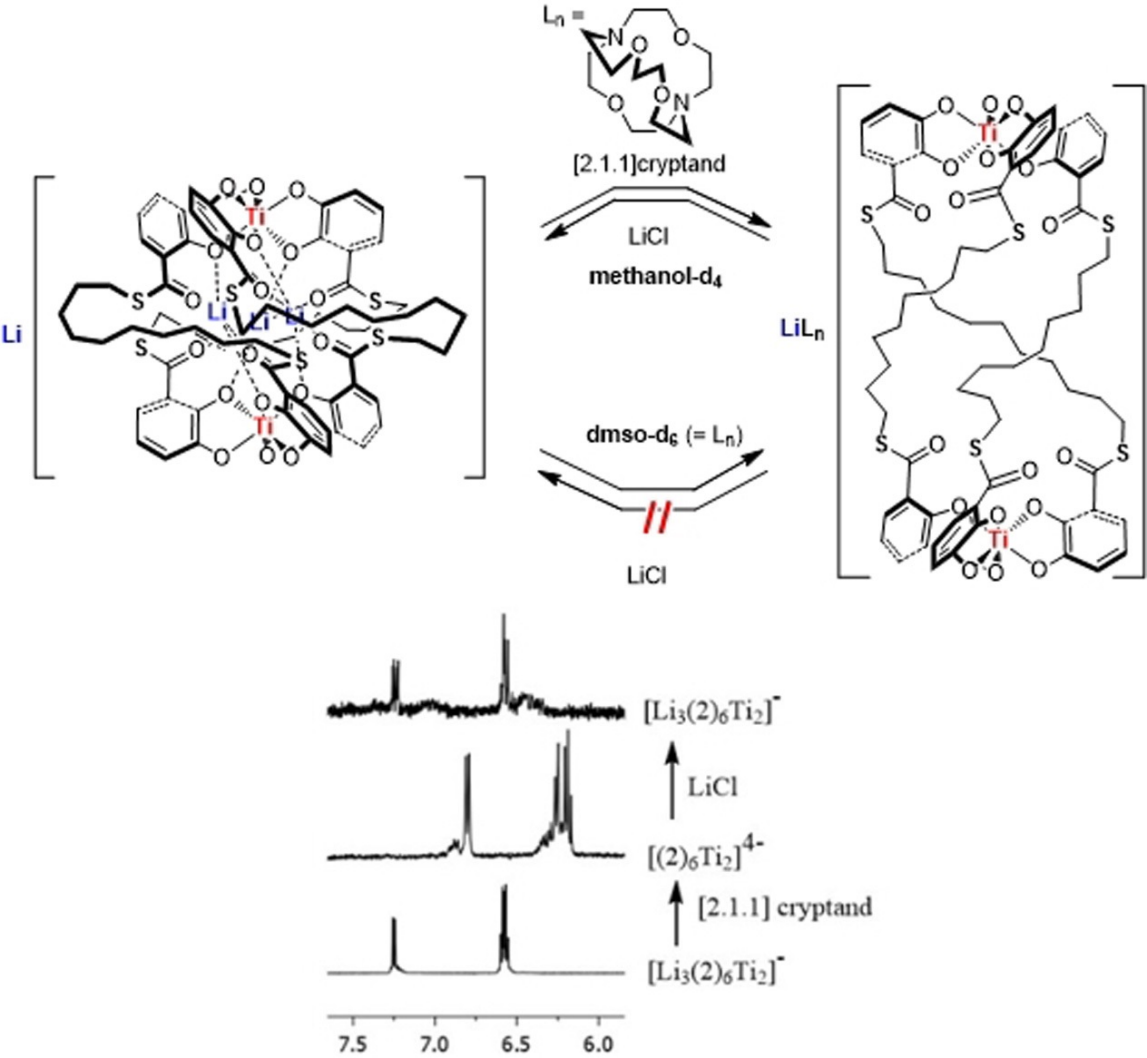
Lithium cation and solvent dependent expansion and compression of Li[Li_3_(**2**)_3_Ti_2_] to [Li([2.1.1]cryptand)]_4_[(**2**)_3_Ti_2_]. This process is reversible in [D_4_]MeOH at room temperature, as can be seen by following the signals of the catechol protons. The quality of the NMR spectra decreases in each switching step due to the addition of excess cryptand or LiCl to the NMR tube (bottom).

**Figure 5:**
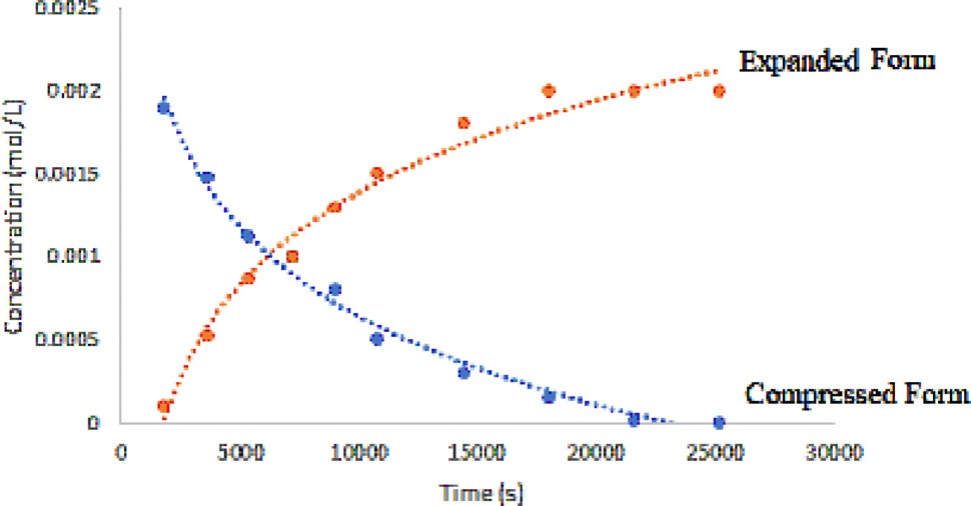
Time‐dependent expansion of Li[Li_3_(**2**)_3_Ti_2_] upon dissolution in [D_6_]DMSO at room temperature (initial concentration: 2×10^−3^ mol L^−1^).

The compressed lithium‐containing complex Li[Li_3_(**2**)_3_Ti_2_] displays a highly dominating signal in the negative ESI MS at *m*/*z=*1539.294 for [Li_3_(**2**)_3_Ti_2_]^−^ (calcd: *m*/*z=*1539.295). The ^1^H NMR in [D_4_]MeOH reveals characteristic signals for aromatic protons at *δ*=7.25 (dd, 1 H) and 6.56 (m, 2 H) and of the diastereotopic SCH_2_ protons at *δ*=2.59 (m, 1 H), and 1.99 ppm (m, 1 H). Addition of [2.1.1]cryptand changes the spectrum significantly which can be best observed following the aromatic protons (Figure 4). They now appear for the dominating species as three separate signals at *δ*=6.95, 6.42, and 6.35 ppm. A similar spectrum has been obtained for K_4_[(**2**)_3_Ti_2_]. This indicates the expansion of the spring type complex to form [(**2**)_3_Ti_2_]^4−^. Upon addition of an excess of lithium chloride, the spectrum of the compressed compound [Li_3_(**2**)_3_Ti_2_]^−^ is restored again. This behavior is the same as has been observed for the oxoesters in DMSO.^7^ Directly after dissolution of Li[Li_3_(**2**)_3_Ti_2_] in [D_6_]DMSO, a typical spectrum of the compressed helicate Li[Li_3_(**2**)_3_Ti_2_] is observed with aromatic catechol signals found at *δ*=7.1, 6.5, and 6.4 ppm. With time, the signal intensity decreases at room temperature and a new set of signals grows in at *δ*=6.8, 6.2, and 6.1 ppm until full conversion is obtained. The new signals are similar to those observed for the expanded potassium complex K_4_[(**2**)_3_Ti_2_]. Following the kinetics of the expansion for a solution of 2×10^−3^ mol L^−1^, a rate constant of *k*=2.2±0.4×10^−4^ s^−1^ is obtained for the first‐order expansion reaction at room temperature (Figure 5).

## Conclusions

Herein, we introduced the thioester moiety as a new functionality to facilitate the lithium‐dependent hierarchical formation of dinuclear triscatecholate titanium(IV) based helicates. Dimerization constants of the thioester derivatives are much lower than observed for corresponding oxoesters but higher than for corresponding ketones. Dimerization constants strongly depend on the solvent as well as on the kind of thioester substituent. For the thioesters, the substituents are electronically well isolated from the carbonyl unit and *K*_dim_ within the series of complexes is mainly influenced by side‐chain–side‐chain interactions caused by solvophobicity/solvophilicity or maybe by dispersion interactions.

Regarding the oxoesters, a molecular switch was created by introducing an alkyl tether at the complex units which easily can be switched in methanol between an expanded and compressed state. However, DMSO as solvent is a strong competitor for lithium‐cation binding so that in this solvent the complex spontaneously releases lithium cations and expands. The kinetics of this process can be easily followed by NMR spectroscopy.

Herein, a new class of compounds is added to the family of hierarchically formed dinuclear titanium(IV) catecholates. Those show a distinct different dimerization behavior compared to the earlier investigated oxoesters, ketones, and aldehydes, which is important for the development of functional switches and switchable catalysts with tunable switching properties.

## Conflict of interest

The authors declare no conflict of interest.

## Supporting information

As a service to our authors and readers, this journal provides supporting information supplied by the authors. Such materials are peer reviewed and may be re‐organized for online delivery, but are not copy‐edited or typeset. Technical support issues arising from supporting information (other than missing files) should be addressed to the authors.

Supplementary
