# Supplementary material for: Catechol Thioesters: Ligands for Hierarchically Formed Lithium‐Bridged Titanium(IV) Helicates and Helicate‐Based Switches
Source: Chemistry. 2020 Mar 5;26(17):3829–33. doi: 10.1002/chem.201905212 (PMC7154688; doi:10.1002/chem.201905212)
Supplement: Supplementary file 1 — Supplementary [file CHEM-26-3829-s001.pdf]

CHEMISTRY
A European Journal

Supporting Information

Catechol Thioesters: Ligands for Hierarchically Formed Lithium-Bridged Titanium(IV) Helicates and Helicate-Based Switches

A. Carel N. Kwamen,[a] Gilles S. de Macedo,[a] Constanze Wiederhold,[b] Iris M. Oppel,[b] and Markus Albrecht*[a]

chem_201905212_sm_miscellaneous_information.pdf

Table of Content

1. Materials and Methods 2

2. Preparation of ligands and complexes 2

3. Crystallographic studies 45

1. Materials and Methods

2,3-Dimethoxybenzoic acid was obtained from Sigma Aldrich, thionyl chloride from Acros Organics, triethylamine from Alfa Aesar. All solvents were used after distillation. MeOH- d_4 was purchased from euriso-top. NMR spectra were obtained with Varian VNMRS 400 and 600 NMR spectrometers. The compounds were analytically characterized on a LTQ Orbitrap XL for ESI-MS. IR measurements were performed using a Perkin-Elmer 100 spectrometer and Elemental analysis measured with a Heraeus CHN-O-Rapid. Melting points were determined with a BÜCHI B-540 melting point instrument and are reported uncorrected.

General esterification procedure:

To a solution of 2,3-Dimethoxybenzoic acid (1 eq.), DCC (3 eq.), DMAP (1.5 eq.) in DCM was added the corresponding alkanethiol (1 eq.). The resulting solution was stirred at RT overnight, then filtered, evaporated and purified via column chromatography. The obtained 2,3-dimethoxybenzyl ester intermediate was dissolved in DCM and was demethylated overnight at RT with BBr_3. Methanol was added to quench the reaction before removing the solvent under vacuum. The product is obtained after collecting the residue in DCM, washing with water, drying over $MgSO_4$, removing the solvent and purifying via column chromatography.

General complexation procedure:

The catechol thioester ligand (3 eq.) is mixed with $TiO(acac)_2$ (1 eq.) and Li_2CO_3 (1 eq.) and dissolved in methanol. The pure complexes are obtained after stirring this solution for one day followed by removal of the solvent. No further purification is necessary due to quantitative complexation.

2. Preparation of ligands and complexes

Ligands 1(a-p)-H$_2$

Methyl-2,3-dihydroxybenzothioate (1a-H$_2$):

The ligand is synthesized with sodium methanethiolate (76.95 mg, 1.10 mmol) according to the general procedure. Column chromatography (DCM, R_f = 0.21) results in the product as a yellowish solid (64.5 %, 130 mg, 0.71 mmol). **M.p.**: 77 °C - 78 °C (last solvent used: DCM). **^{1}H NMR** (600 MHz, $CDCl_3$): δ = 11.20 (s, 1H, OH), 7.36 (dd, *J* = 8.0, 1.6 Hz, 1H, $H_{arom.}$), 7.10 (dd, *J* = 8.0, 1.6 Hz, 1H, $H_{arom.}$), 6.81 (t, *J* = 8.0 Hz, 1H, $H_{arom.}$), 5.67 (s, 1H, OH), 2.48 (s, 3H, SCH_3) ppm. **^{13}C NMR** (151 MHz, $CDCl_3$): δ = 198.01 ($COSCH_2$), 146.50 (C_{arom}), 145.28 (C_{arom}), 119.84 (C_{arom}), 119.77 (C_{arom}), 119.47 (C_{arom}), 119.40 (C_{arom}), 11.50 (SCH_3) ppm. **MS** (negative and positive ESI-MS, MeOH, acidified): *m/z* (%) = 183.0105 (100, [M-H$^+$], $C_8H_7O_3S^-$, calcd. 183.0116; 207.0084 (30, [M+Na$^+$], $C_8H_8O_3SNa^+$, calcd. 207.0092). **IR** (KBr):$\tilde{\nu}$ (cm^{-1}) = 3900, 3463, 3082, 2926, 2853, 2350, 2151, 2080, 2019, 1989, 1928, 1865, 1672, 1627, 1591, 1485, 1447, 1337, 1259, 1203, 1081, 1026, 965, 902, 853, 788, 729, 661. **Elemental Analysis**: $C_8H_8O_3S$: calcd. C = 52.16 %, H = 4.38 %; found C = 52.09 %, H = 4.30 %.

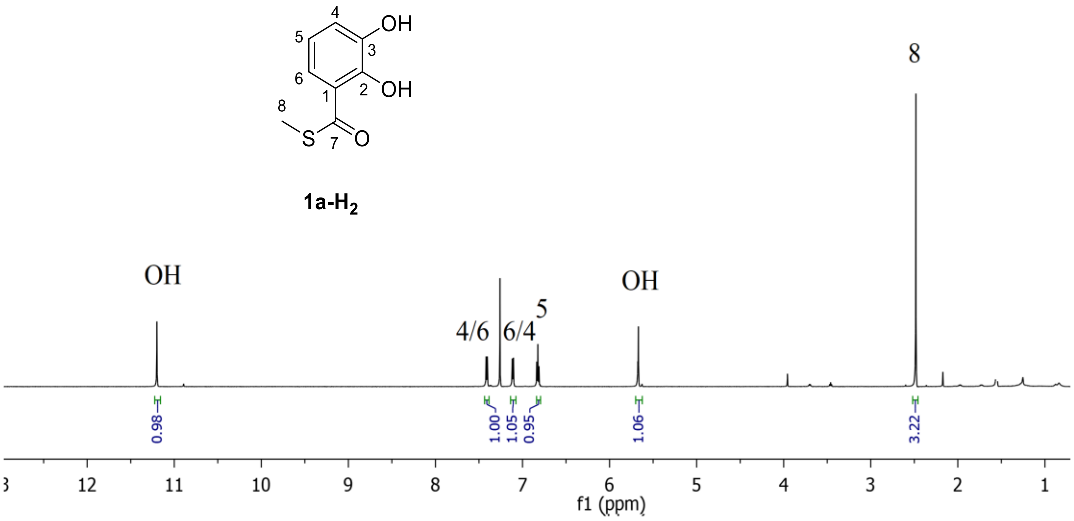

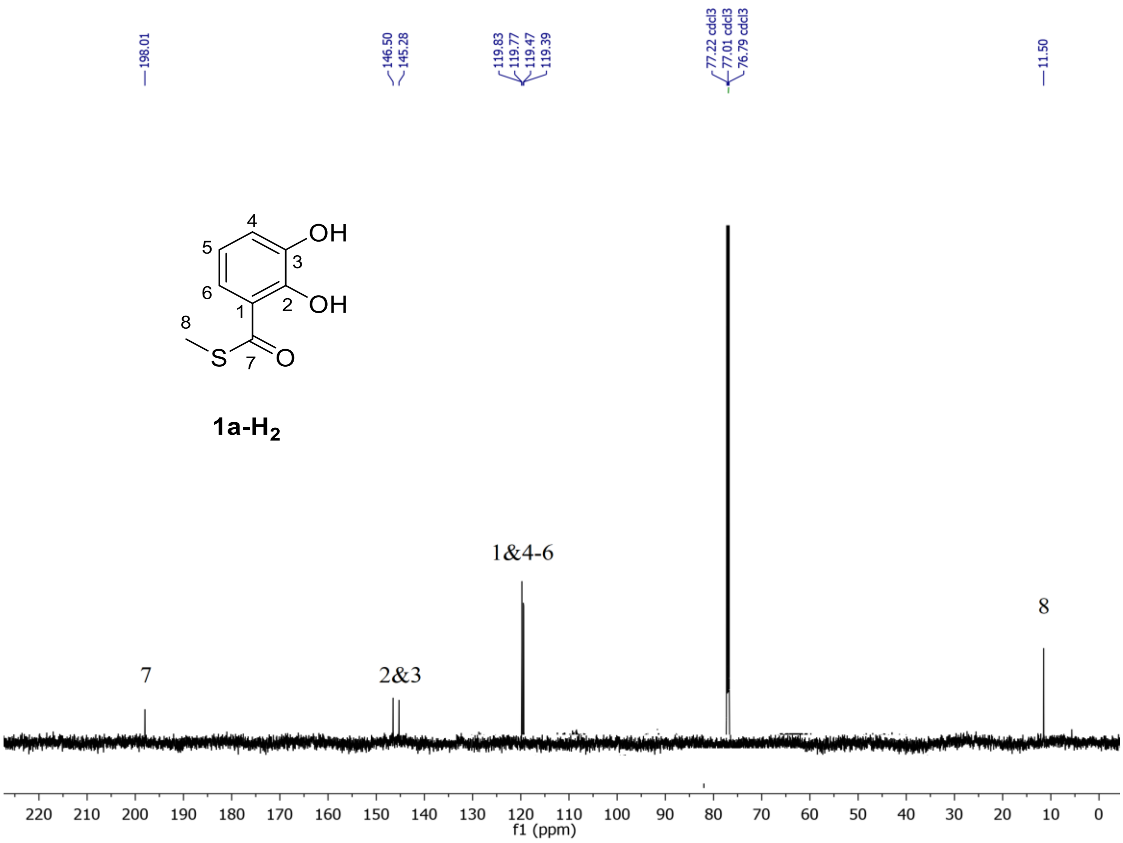

Ethyl-2,3-dihydroxybenzothioate (1b-H_2):

The ligand is synthesized with ethanethiol (0.08 mL, 1.1 mmol) according to the general procedure. Column chromatography (pentane/ethyl acetate 8:1, R_f = 0.22) results in the product as a yellowish oil (69 %, 150 mg, 0.76 mmol). **^{1}H NMR** (400 MHz, $CDCl_3$): δ = 11.23 (s, 1H, OH), 7.38 (dd, *J* = 7.9, 1.5 Hz, 1H, $H_{arom.}$), 7.09 (dd, *J* = 7.9, 1.5 Hz, 1H, $H_{arom.}$), 6.79 (t, *J* = 7.9 Hz, 1H, $H_{arom.}$), 5.68 (s, 1H, OH), 23.06 (q, *J* = 7.4 Hz, 2H, SCH_2), 1.38 (t, *J* = 7.5 Hz, 3H, CH_2CH_3) ppm. **^{13}C NMR** (101 MHz, $CDCl_3$): δ = 197.83 (*C*$OSCH_2$), 146.64 (C_{arom}), 145.28 (C_{arom}), 119.94 (C_{arom}), 119.72 (C_{arom}), 119.43 (C_{arom}), 119.35 (C_{arom}),

23.33 (SCH_2), 14.49 (CH_2CH_3) ppm. **MS** (negative and positive ESI-MS, MeOH, acidified): *m/z* (%) = 197.0283 (100, [M-H$^+$], $C_9H_9O_3S^-$, calcd. 197.0272; 221.0240 (30, [M+Na$^+$], $C_9H_{10}O_3SNa^+$, calcd. 221.0273). **IR** (KBr): $\tilde{\nu}$ (cm^{-1}) = 3467, 3057, 2970, 2933, 2869, 2722, 2486, 2327, 2113, 1916, 1620, 1454, 1359, 1251, 1024, 855, 789, 725. **Elemental Analysis**: $C_9H_{10}O_3S$ · 1/4 pentane: calcd. C = 56.92 %, H = 6.06 %; found C = 56.01 %, H = 6.07 %.

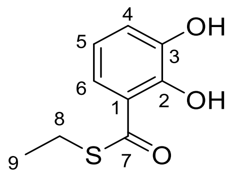

1b-H$_2$

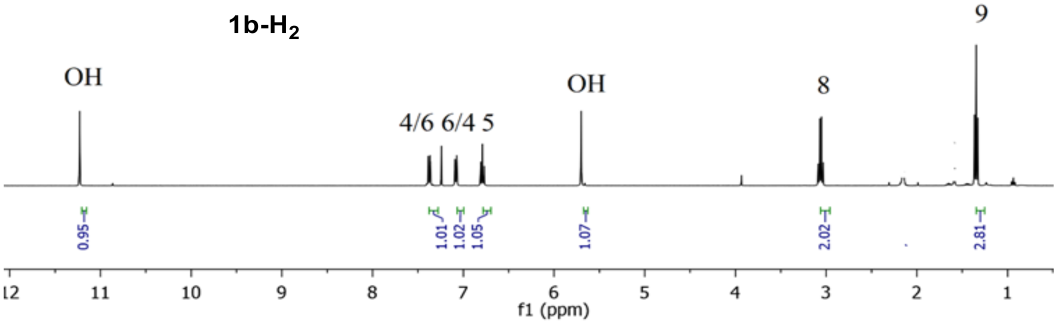

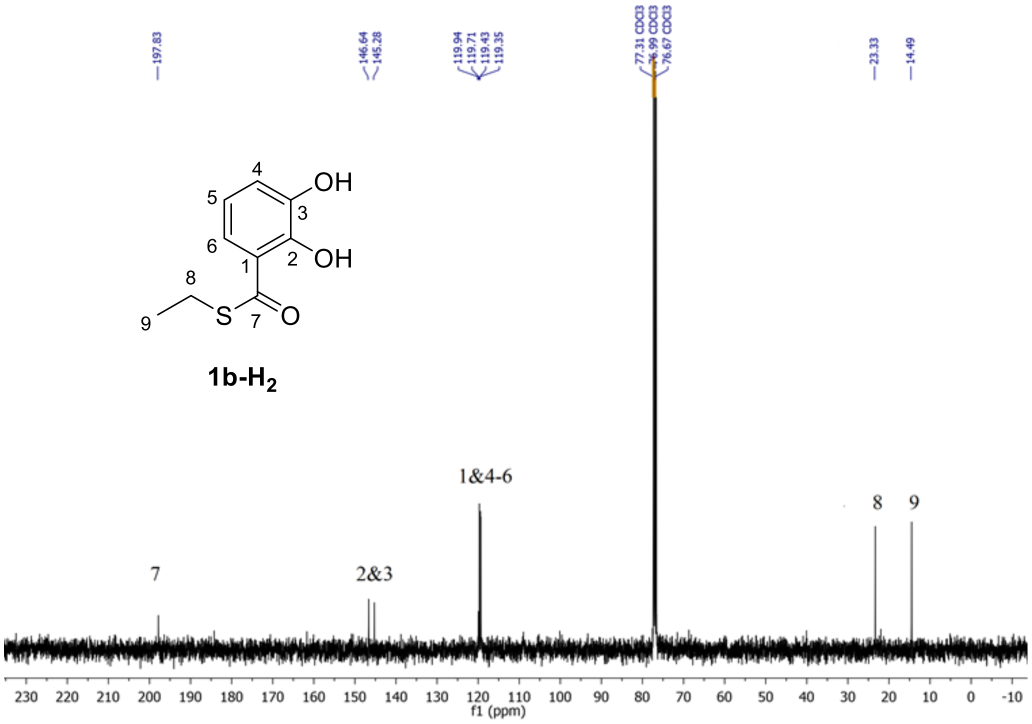

1b-H$_2$

Propyl-2,3-dihydroxybenzothioate (1c-H$_2$):

The ligand is synthesized with 1-propanethiol (0.10 mL, 1.1 mmol) according to the general procedure. Column chromatography (pentane/ethyl acetate 4:1, R_f = 0.20) results in the product as a yellowish oil (70 %, 140 mg, 0.66 mmol). **^{1}H NMR** (600 MHz, $CDCl_3$): δ = 11.26 (s, 1H, OH), 7.42 (dd, *J* =

8.1, 1.2 Hz, 1H, $H_{arom.}$), 7.10 (dd, *J* = 8.1, 1.2 Hz, 1H, $H_{arom.}$), 6.81 (t, *J* = 8.1 Hz, 1H, $H_{arom.}$), 5.67 (s, 1H, OH), 3.06 (q, *J* = 7.3 Hz, 2H, SCH_2), 1.77-1.75 (m, 2H, CH_2CH_2), 1.04 (t, *J* = 7.4 Hz, 3H, CH_2CH_3) ppm. **^{13}C NMR** (151 MHz, $CDCl_3$): δ = 197.86 (*C*$OSCH_2$), 146.63 (C_{arom}), 145.27 (C_{arom}), 119.96 (C_{arom}), 119.69 (C_{arom}), 119.50 (C_{arom}), 119.36 (C_{arom}), 30.74 (SCH_2), 22.75 (CH_2CH_2), 13.40 (CH_2CH_3) ppm. **MS** (negative and positive ESI-MS, MeOH, acidified): *m/z* (%) = 211.0430 (100, [$M-H^+$], $C_{10}H_{11}O_3S^-$, calcd. 211.0429; 235.0410 (100, [$M+Na^+$], $C_{10}H_{12}O_3SNa^+$, calcd. 235.0405). **IR** (KBr): ṽ (cm^{-1}) = 3469, 3063, 2965, 2930, 2873, 2060, 1908, 1628, 1597, 1458, 1375, 1266, 1166, 1078, 1029, 855, 793, 731, 659, 577, 482. **Elemental Analysis**: $C_{10}H_{12}O_3S$: calcd. C = 56.59 %, H = 5.70 %; found C = 56.32 %, H = 5.78 %.

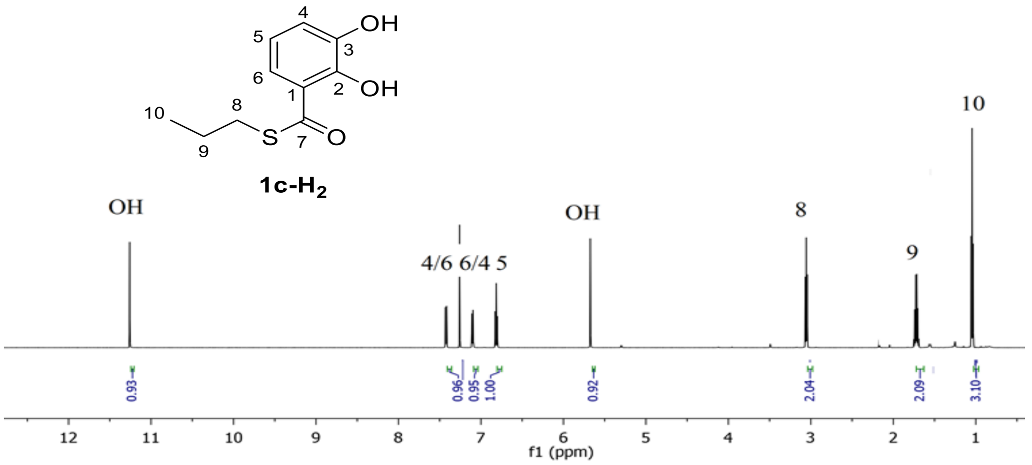

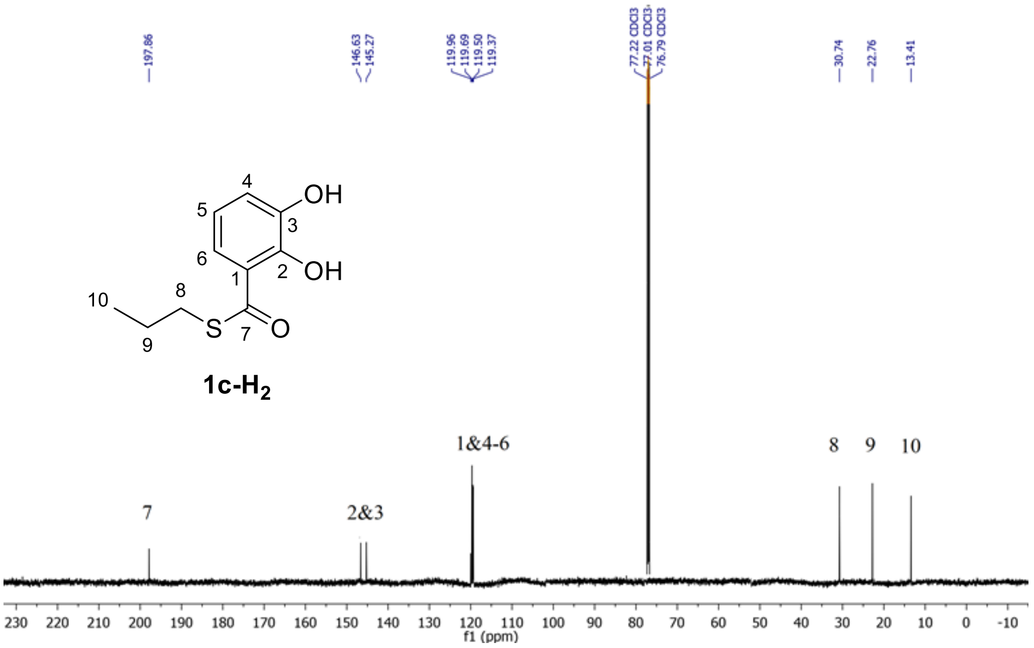

Butyl-2,3-dihydroxybenzothioate (1d-H_2):

The ligand is synthesized with 1-butanethiol (0.12 mL, 1.1 mmol) according to the general procedure. Column chromatography (pentane/ethyl acetate 4:1, R_f = 0.24) results in the product as a yellowish oil (63 %, 156 mg, 0.69 mmol). **^{1}H NMR** (600 MHz, $CDCl_3$): δ = 11.25 (s, 1H, OH), 7.42 (dd, *J* = 8.0, 1.5

Hz, 1H, $H_{arom.}$), 7.10 (dd, *J* = 8.0, 1.5 Hz, 1H, $H_{arom.}$), 6.81 (t, *J* = 8.0 Hz, 1H, $H_{arom.}$), 5.67 (s, 1H, OH), 3.08 (t, *J* = 7.4 Hz, 2H, SCH_2), 1.68-1.66 (m, 2H, CH_2CH_2), 1.47-1.45 (m, 2H, CH_2CH_2), 0.96 (t, *J* = 7.4 Hz, 3H, CH_2CH_3) ppm. **^{13}C NMR** (151 MHz, $CDCl_3$): δ = 197.87 (*C*$OSCH_2$), 146.62 (C_{arom}), 145.27 (C_{arom}), 119.96 (C_{arom}), 119.67 (C_{arom}), 119.49 (C_{arom}), 119.36 (C_{arom}), 31.33 (CH_2CH_2), 28.57 (SCH_2), 22.00 (CH_2CH_2), 13.59 (CH_2CH_3) ppm. **MS** (negative and positive ESI-MS, MeOH, acidified): *m/z* (%) = 225.0596 (100, [$M-H^+$], $C_{11}H_{13}O_3S^-$, calcd. 225.0585; 249.0556 (100, [$M+Na^+$], $C_{11}H_{14}O_3SNa^+$, calcd. 249.0561). **IR** (KBr): $\tilde{\nu}$ (cm^{-1}) = 3470, 3057, 2956, 2867, 2727, 2326, 2166, 1914, 1625, 1595, 1455, 1362, 1257, 1080, 1025, 855, 790, 727, 659. **Elemental Analysis**: $C_{11}H_{14}O_3S \cdot 1/2\ H_2O$: calcd. C = 56.15 %, H = 6.43 %; found C = 56.30 %, H = 6.99 %.

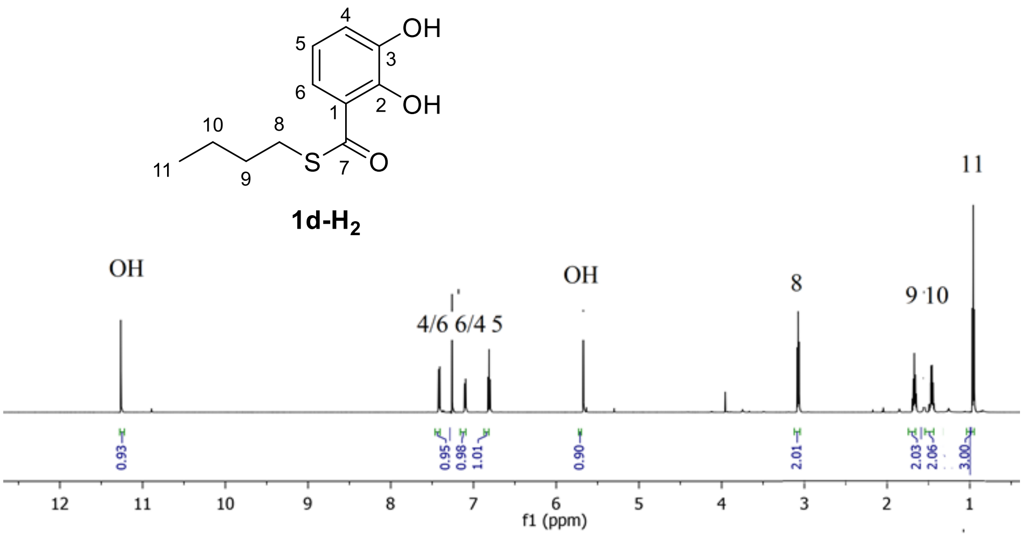

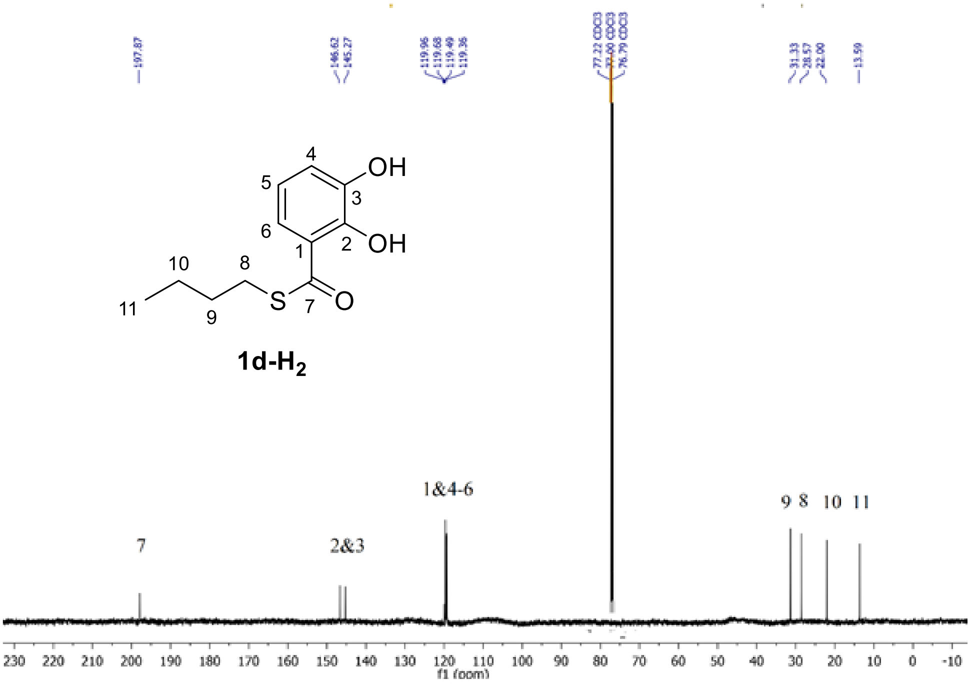

Pentyl-2,3-dihydroxybenzothioate (1e-H_2):

The ligand is synthesized with 1-pentanethiol (114.63 mg, 1.1 mmol) according to the general procedure. Column chromatography (DCM, R_f = 0.25) results in the product as a colourless oil (45 %, 120 mg, 0.50 mmol). **^{1}H NMR** (600 MHz, $CDCl_3$): δ = 11.27 (s, 1H, OH), 7.41 (dd, *J* = 8.0, 1.2 Hz, 1H, $H_{arom.}$), 7.10 (dd, *J* = 8.0, 1.2 Hz, 1H, $H_{arom.}$), 6.82 (t, *J* = 8.0 Hz, 1H, $H_{arom.}$), 5.69 (s, 1H, OH), 3.06 (t, *J* = 7.4 Hz, 2H, SCH_2), 1.69-1.67 (m, 2H, CH_2CH_2), 1.42-1.39 (m, 4H, 2×CH_2), 0.92 (t, *J* = 7.2 Hz, 3H, CH_2CH_3) ppm. **^{13}C NMR** (151 MHz, $CDCl_3$): δ = 197.88 (*C*$OSCH_2$), 146.63 (C_{arom}), 145.28 (C_{arom}), 119.97 (C_{arom}), 119.69 (C_{arom}), 119.49 (C_{arom}), 119.36 (C_{arom}), 31.00 (CH_2CH_2), 28.99 (SCH_2), 28.84 (CH_2CH_2), 22.21 (CH_2CH_2), 13.93 (CH_2CH_3) ppm. **MS** (negative and positive ESI-MS, MeOH, acidified): *m/z* (%) = 239.0747 (100, [M-H$^+$], $C_{12}H_{15}O_3S^-$, calcd. 239.0742; 263.0704 (20, [M+Na$^+$], $C_{12}H_{16}O_3SNa^+$, calcd. 263.0718). **IR** (KBr): $\tilde{\upsilon}$ (cm^{-1}) = 3469, 3061, 2928, 2861, 2663, 2326, 2083, 1992, 1905, 1627, 1595, 1455, 1369, 1257, 1165, 1079, 1027, 854, 791, 729, 659. **Elemental Analysis**: $C_{12}H_{16}O_3S$: calcd. C = 59.98 %, H = 6.71 %; found C = 59.89 %, H = 6.50 %.

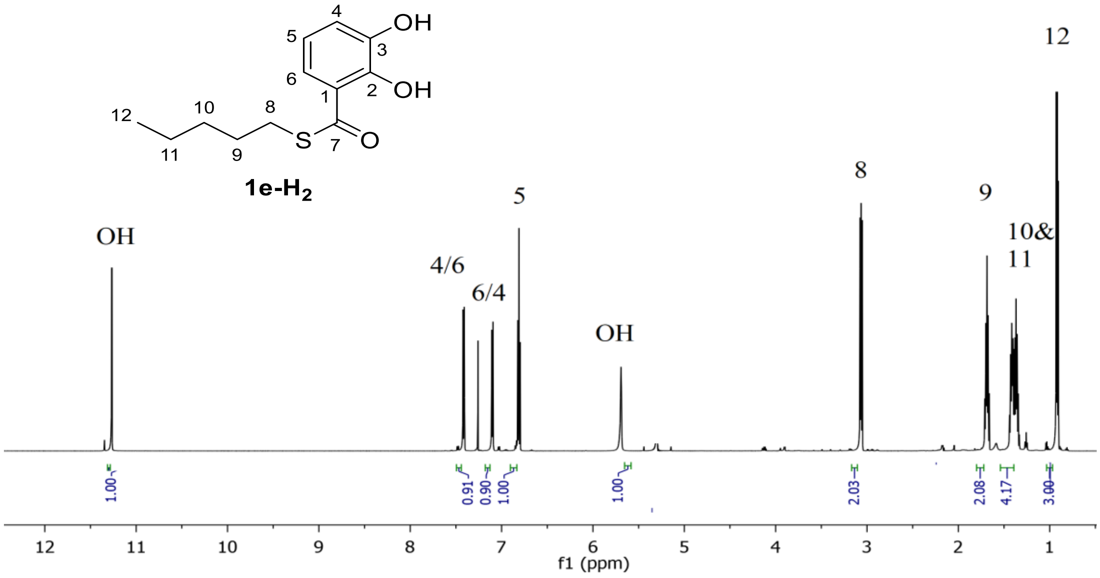

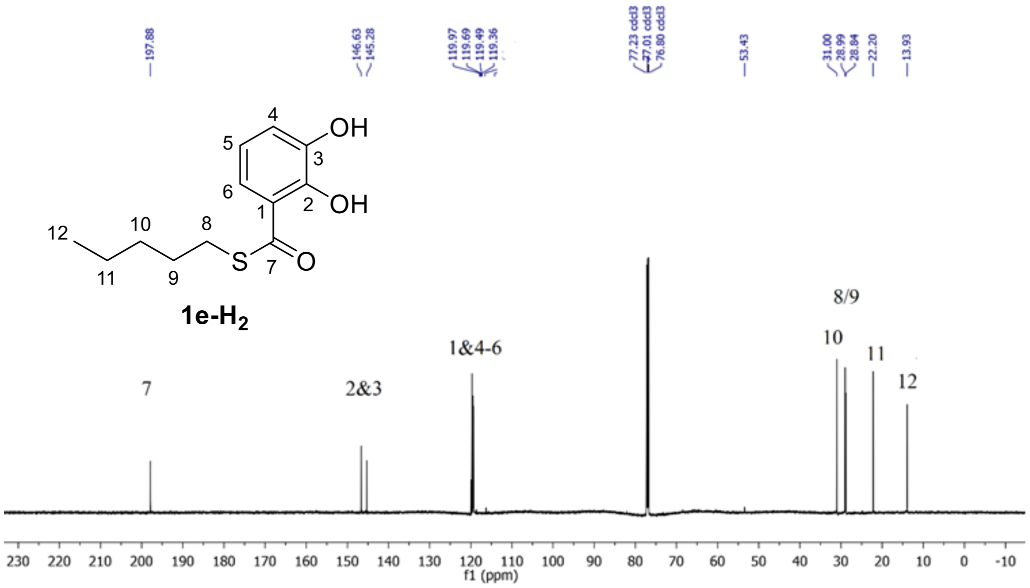

Hexyl-2,3-dihydroxybenzothioate (1f-H_2):

The ligand is synthesized with 1-hexanethiol (0.16 mL, 1.1 mmol) according to the general procedure. Column chromatography (DCM, R_f = 0.23) results in the product as a yellowish solid (44 %, 122 mg, 0.48 mmol). **M.p.**: 98 °C - 99 °C (last solvent used: DCM). **^{1}H NMR** (600 MHz, $CDCl_3$): δ = 11.30 (s, 1H, OH), 7.41 (dd, *J* = 7.9, 1.3 Hz, 1H, $H_{arom.}$), 7.08 (dd, *J* = 7.9, 1.3 Hz, 1H, $H_{arom.}$), 6.81 (t, *J* = 7.9 Hz, 1H, $H_{arom.}$), 5.65 (s, 1H, OH), 3.07 (t, *J* = 7.4 Hz, 2H, SCH_2), 1.69-1.67 (m, 2H, CH_2CH_2), 1.44-1.42 (m, 2H, CH_2CH_2), 1.37-1.35 (m, 4H, 2×CH_2), 0.89 (t, *J* = 7.4 Hz, 3H, CH_2CH_3) ppm. **^{13}C NMR** (151 MHz, $CDCl_3$): δ = 197.87 (*C*$OSCH_2$), 146.63 (C_{arom}), 145.28 (C_{arom}), 119.99 (C_{arom}), 119.68 (C_{arom}), 119.49 (C_{arom}), 119.35 (C_{arom}), 31.77 (CH_2CH_2), 29.25 (SCH_2), 29.06 (CH_2CH_2), 28.88 (CH_2CH_2), 22.62 (CH_2CH_2), 13.99 (CH_2CH_3) ppm. **MS** (negative and positive ESI-MS, MeOH, acidified): *m/z* (%) = 253.0902 (100, [M-H^+], $C_{13}H_{17}O_3S^-$, calcd. 253.0898; 277.0864 (30, [M+Na^+], $C_{13}H_{18}O_3SNa^+$, calcd. 277.0874). **IR** (KBr): ṽ (cm^{-1}) = 3469, 3062, 2927, 2858, 2059, 1904, 1628, 1597, 1458, 1373, 1265, 1165, 1079, 1030, 855, 793, 730, 659, 577, 481. **Elemental Analysis**: $C_{13}H_{18}O_3S$: calcd. C = 61.39 %, H = 7.13 %; found C = 61.56 %, H = 7.13 %.

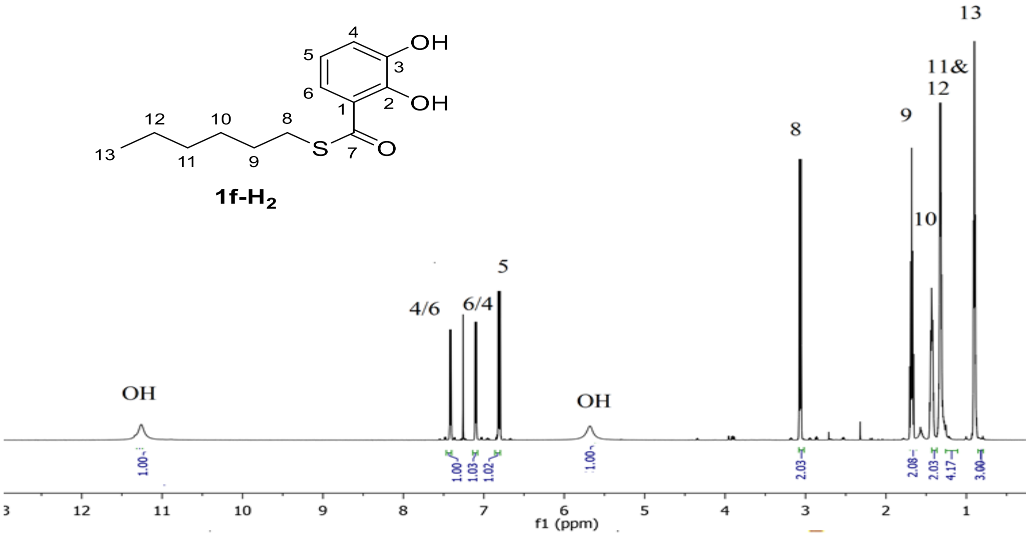

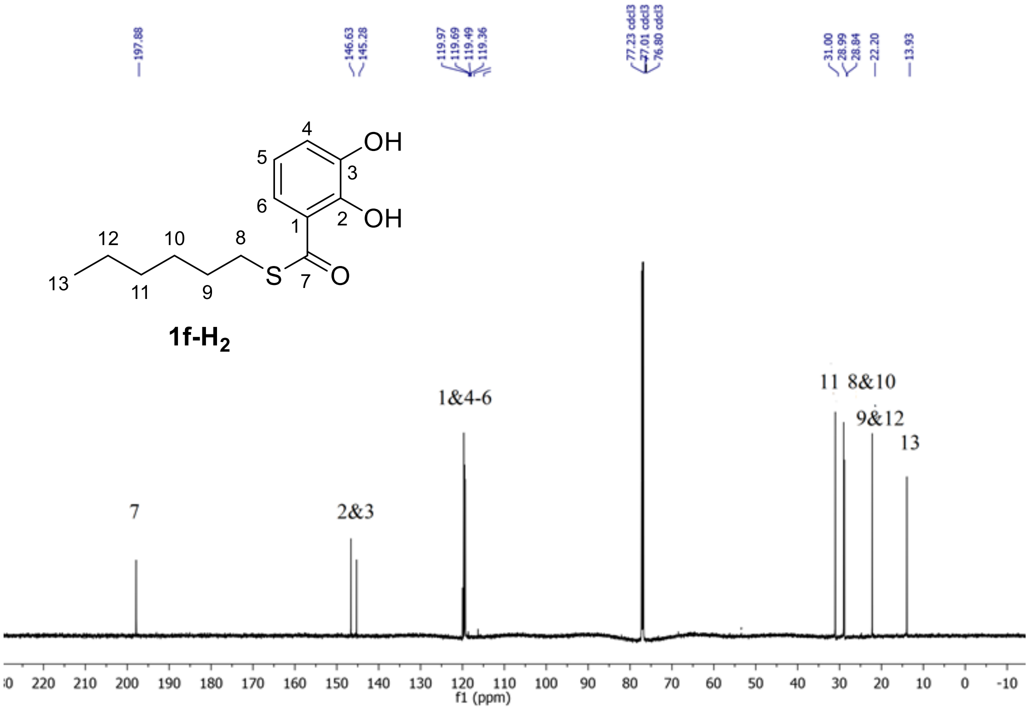

Heptyl-2,3-dihydroxybenzothioate (1g-H_2):

The ligand is synthesized with 1-heptanethiol (142.50 mg, 1.1 mmol) according to the general procedure. Column chromatography (DCM, R_f = 0.18) results in the product as a yellowish oil (37 %, 110 mg, 0.41 mmol). **^{1}H NMR** (600 MHz, $CDCl_3$): δ = 11.27 (s, 1H, OH), 7.42 (dd, *J* = 8.1, 1.4 Hz, 1H, $H_{arom.}$), 7.10 (dd, *J* = 8.1, 1.4 Hz, 1H, $H_{arom.}$), 6.81 (t, *J* = 8.1 Hz, 1H, $H_{arom.}$), 5.66 (s, 1H, OH), 3.05 (t, *J* = 7.4 Hz, 2H, SCH_2), 1.70-1.68 (m, 2H, CH_2CH_2), 1.42-1.28 (m, 8H, 4×CH_2), 0.91 (t, *J* = 6.9 Hz, 3H, CH_2CH_3) ppm. **^{13}C NMR** (151 MHz, $CDCl_3$): δ = 197.88 (*C*OSCH_2), 146.63 (C_{arom}), 145.28 (C_{arom}), 119.97 (C_{arom}), 119.67 (C_{arom}), 119.49 (C_{arom}), 119.36 (C_{arom}), 31.67 (CH_2CH_2), 29.30 (SCH_2), 28.88 (CH_2CH_2), 28.84 (CH_2CH_2), 28.77 (CH_2CH_2), 22.20 (CH_2CH_2), 13.93 (CH_2CH_3) ppm. **MS** (negative and positive ESI-MS,

MeOH, acidified): *m/z* (%) = 267.1063 (100, [M-H⁺], $C_{14}H_{19}O_3S^-$, calcd. 267.1057; 291.1023 (40, [M+Na⁺], $C_{14}H_{20}O_3SNa^+$, calcd. 291.1031). **IR** (KBr): $\tilde{\nu}$ (cm^{-1}) = 3889, 3469, 3062, 2925, 2856, 2723, 2329, 2115, 1912, 1627, 1596, 1518, 1456, 1370, 1259, 1165, 1079, 1027, 854, 791, 728, 659. **Elemental Analysis**: $C_{14}H_{20}O_3S$: calcd. C = 62.66 %, H = 7.51 %; found C = 62.70 %, H = 7.44 %.

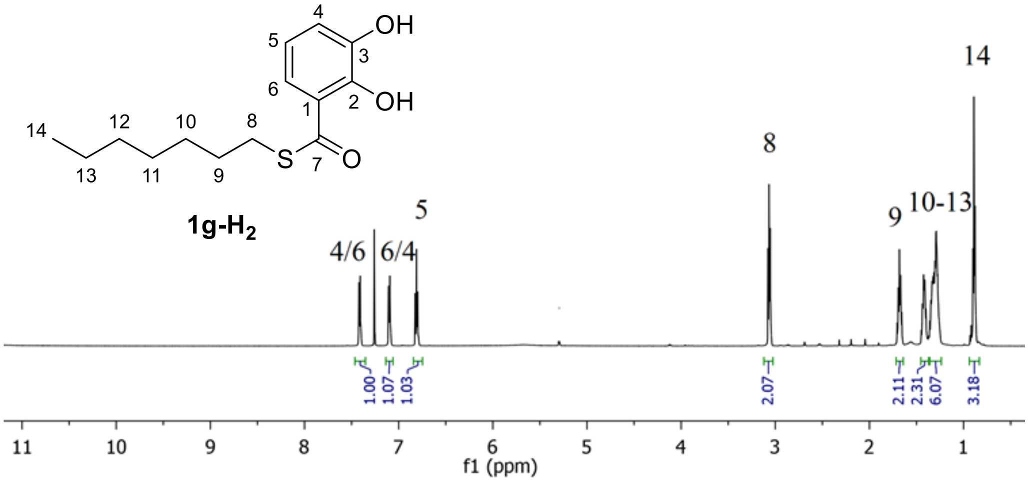

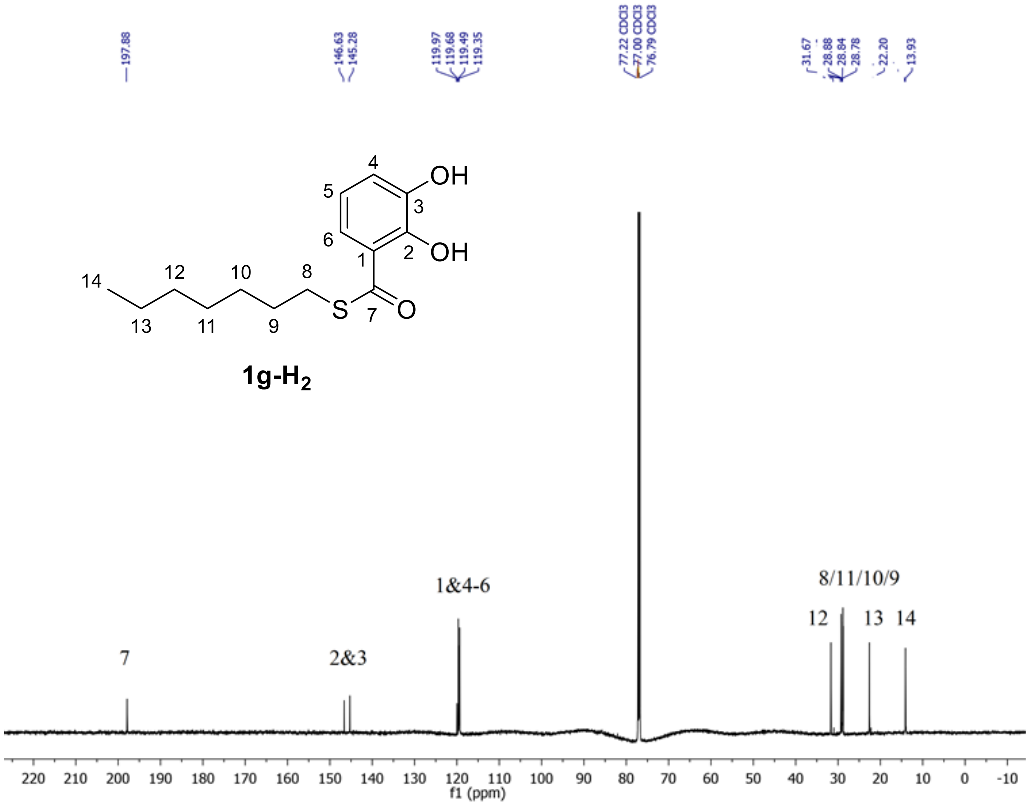

Octyl-2,3-dihydroxybenzothioate (1h-H_2):

The ligand is synthesized with 1-octanethiol (0.19 mL, 1.1 mmol) according to the general procedure. Column chromatography (DCM, R_f = 0.23) results in the product as a colourless oil (48 %, 150 mg, 0.53 mmol). **^{1}H NMR** (600 MHz, $CDCl_3$): δ = 11.26 (s, 1H, OH), 7.42 (dd, *J* = 8.0, 1.3 Hz, 1H, $H_{arom.}$), 7.10 (dd, *J* = 8.0, 1.3 Hz, 1H, $H_{arom.}$), 6.81 (t, *J* = 8.0 Hz, 1H, $H_{arom.}$), 5.66 (s, 1H, OH), 3.07 (t, *J* = 7.4 Hz, 2H, SCH_2), 1.69-1.67 (m, 2H, CH_2CH_2), 1.45-1.39 (m, 2H, CH_2CH_2), 1.36-1.23 (m, 8H, 4×CH_2), 0.89 (t, *J* = 8.5 Hz, 3H, CH_2CH_3) ppm. **^{13}C NMR** (151 MHz, $CDCl_3$): δ = 197.86 (*C*OSCH_2), 146.62 (C_{arom}), 145.28 (C_{arom}), 119.99 (C_{arom}), 119.67 (C_{arom}), 119.49 (C_{arom}), 119.35 (C_{arom}), 31.77 (CH_2CH_2), 29.29 (SCH_2), 29.12 (CH_2CH_2), 29.06 (CH_2CH_2), 28.88 (2×CH_2), 22.62 (CH_2CH_2), 14.07 (CH_2CH_3) ppm. **MS** (negative ESI-MS, MeOH, acidified): *m/z* (%) = 281.1217 (100, [M-H^+], $C_{15}H_{21}O_3S^-$, calcd. 281.1211). **IR** (KBr): $\tilde{\nu}$ (cm^{-1}) = 3468, 3062, 2926, 2856, 2252, 2058, 1902, 1839, 1685, 1628, 1598, 1458, 1371, 1265, 1166, 1079, 1030, 856, 793, 730, 659, 578, 481. **Elemental Analysis**: $C_{15}H_{22}O_3S$: calcd. C = 63.80 %, H = 7.85 %; found C = 63.88 %, H = 7.89 %.

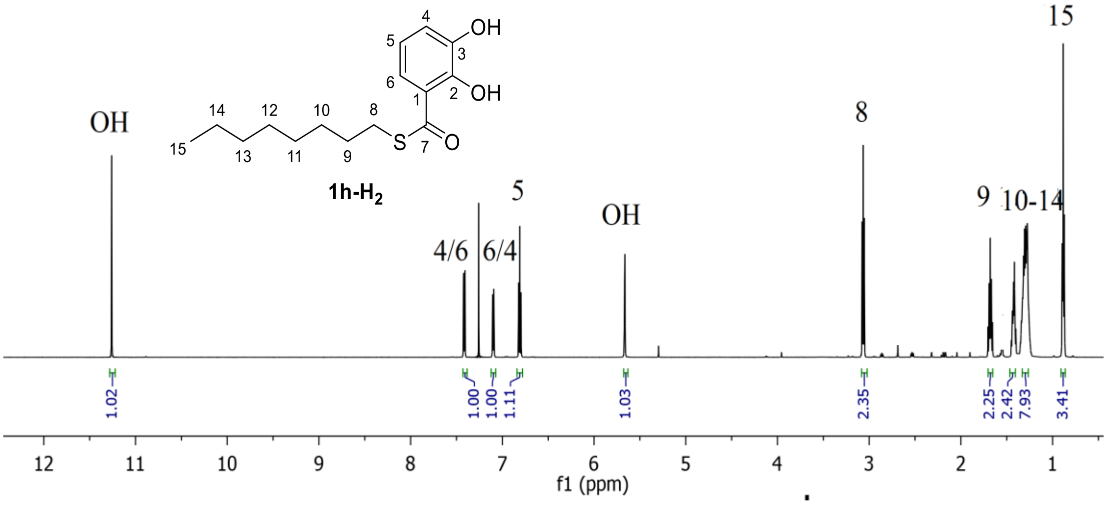

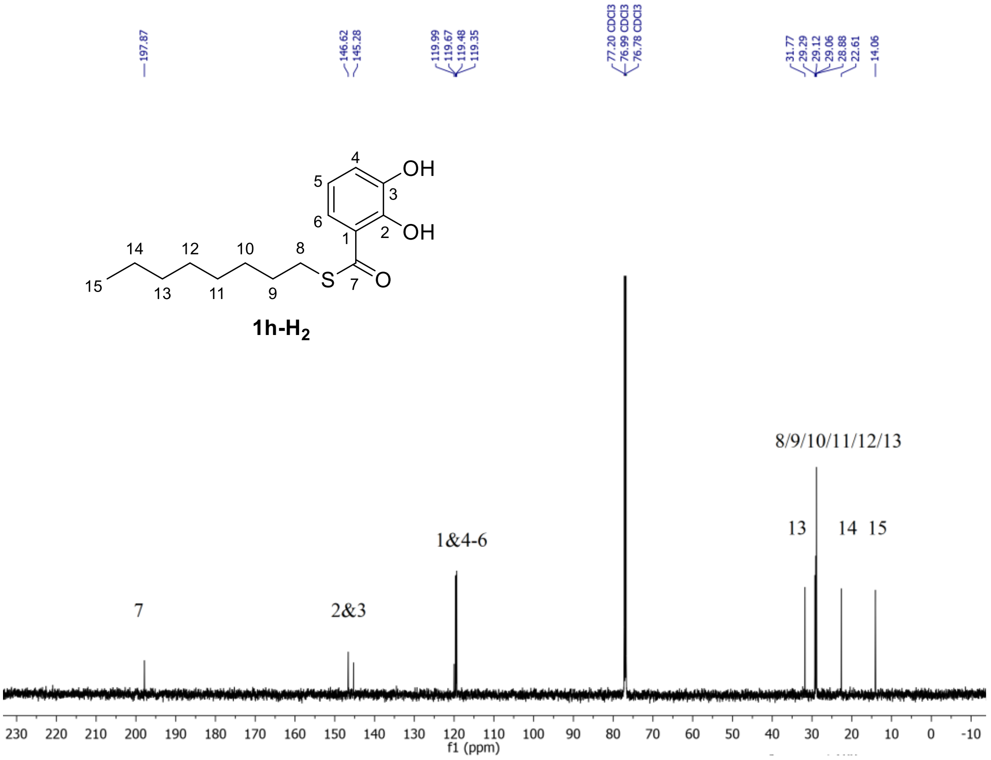

Nonyl-2,3-dihydroxybenzothioate (1i-H_2):

The ligand is synthesized with 1-nonanethiol (176.35 mg, 1.1 mmol) according to the general procedure. Column chromatography (DCM, R_f = 0.21) results in the product as a colourless oil (36 %, 118 mg, 0.40 mmol). **^{1}H NMR** (600 MHz, $CDCl_3$): δ = 11.26 (s, 1H, OH), 7.42 (dd, *J* = 7.9, 1.5 Hz, 1H, $H_{arom.}$), 7.08 (dd, *J* = 7.9, 1.5 Hz, 1H, $H_{arom.}$), 6.81 (t, *J* = 7.5 Hz, 1H, $H_{arom.}$), 5.67 (s, 1H, OH), 3.06 (t, *J* = 7.4 Hz, 2H, SCH_2), 1.69-1.67 (m, 2H, CH_2CH_2), 1.43-1.41 (m, 2H, CH_2CH_2), 1.32-1.30 (m, 10H, 5×CH_2), 0.88 (t, *J* = 8.6 Hz, 3H, CH_2CH_3) ppm. **^{13}C NMR** (151 MHz, $CDCl_3$): δ = 197.89 (*C*$OSCH_2$), 146.62 (C_{arom}), 145.27 (C_{arom}), 119.97 (C_{arom}), 119.67 (C_{arom}), 119.50 (C_{arom}), 119.36 (C_{arom}), 31.84 (CH_2CH_2), 29.43 (SCH_2), 29.30 (CH_2CH_2), 29.23 (CH_2CH_2), 29.12 (CH_2CH_2), 28.88 (2×CH_2), 22.66 (CH_2CH_2), 14.11 (CH_2CH_3) ppm. **MS** (negative and positive ESI-MS, MeOH, acidified): *m/z* (%) = 295.1359 (100, [M-H$^+$], $C_{16}H_{23}O_3S^-$, calcd. 295.1368; 319.1337 (100, [M+Na$^+$], $C_{16}H_{24}O_3SNa^+$, calcd. 319.1344). **IR** (KBr): $\tilde{\nu}$ (cm^{-1}) = 3889, 3470, 3061, 2924, 2854, 2327, 2112, 1992, 1899, 1627, 1596, 1456, 1369, 1259, 1165, 1079, 1027, 854, 791, 728, 659. **Elemental Analysis**: $C_{16}H_{24}O_3S$: calcd. C = 64.83 %, H = 8.16 %; found C = 65.06 %, H = 8.17 %.

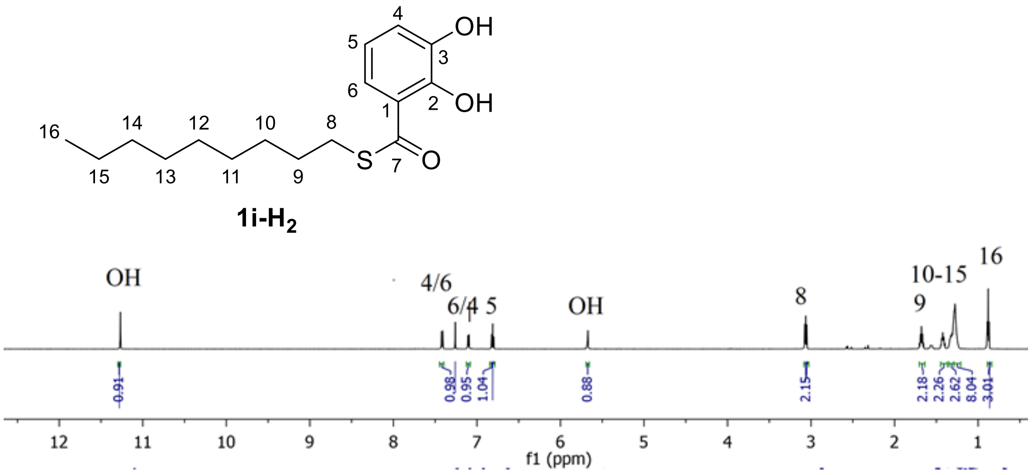

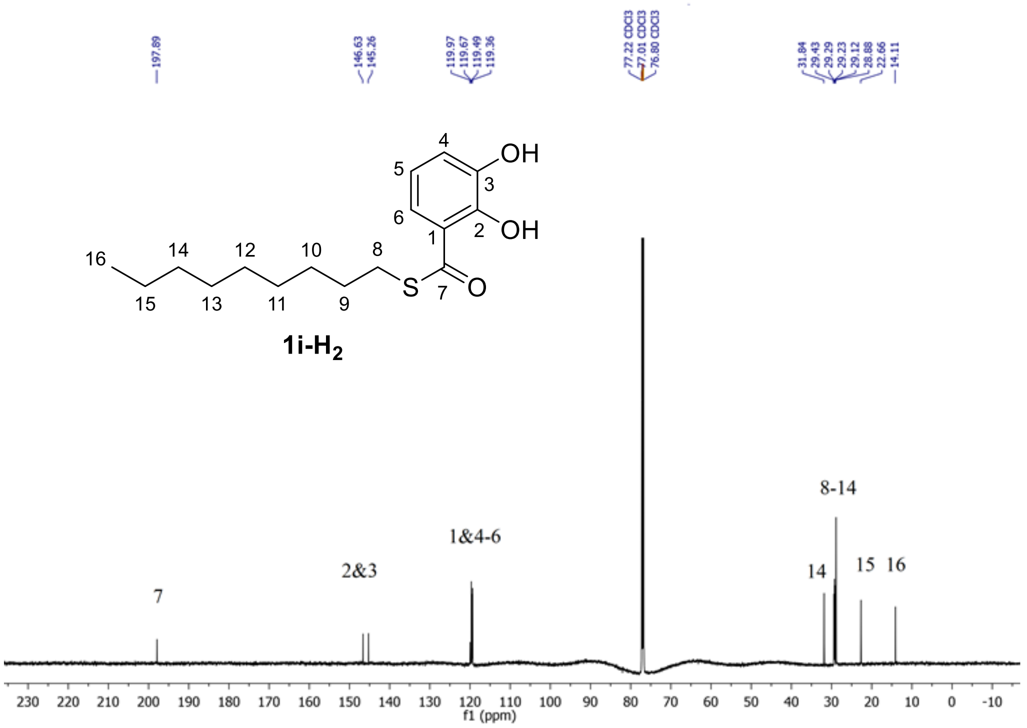

Dodecyl-2,3-dihydroxybenzothioate (1j-H$_2$):

The ligand is synthesized with 1-dodecanethiol (0.26 mL, 1.1 mmol) according to the general procedure. Column chromatography (DCM, R_f = 0.19) results in the product as a yellowish solid (29 %, 110 mg, 0.32 mmol). **M.p.**: 45°C - 46 °C (last solvent used: DCM). **^{1}H NMR** (600 MHz, CDCl$_3$): δ = 11.27 (s, 1H, OH), 7.43 (dd, *J* = 8.0, 1.4 Hz, 1H, H$_{arom.}$), 7.09 (dd, *J* = 8.0, 1.4 Hz, 1H, H$_{arom.}$), 6.81 (t, *J* = 8.0 Hz, 1H, H$_{arom.}$), 5.66 (s, 1H, OH), 3.06 (t, *J* = 7.4 Hz, 2H, SCH$_2$), 1.69-1.67 (m, 2H, CH$_2$CH$_2$), 1.43-1.41 (m, 2H, CH$_2$CH$_2$), 1.32-1.30 (m, 16H, 8×CH$_2$), 0.88 (t, *J* = 7.0 Hz, 3H, CH$_2$CH$_3$) ppm. **^{13}C NMR** (151 MHz, CDCl$_3$): δ = 197.88 (*C*OSCH$_2$), 146.62 (C$_{arom}$), 145.27 (C$_{arom}$), 119.97 (C$_{arom}$), 119.67 (C$_{arom}$), 119.49 (C$_{arom}$), 119.35 (C$_{arom}$), 31.91 (CH$_2$CH$_2$), 29.62 (SCH$_2$), 29.57 (2×CH$_2$), 29.47 (CH$_2$CH$_2$), 29.34 (CH$_2$CH$_2$), 29.29 (CH$_2$CH$_2$), 29.11 (CH$_2$CH$_2$), 28.88 (2×CH$_2$), 22.69 (CH$_2$CH$_2$), 14.12 (CH$_2$CH$_3$) ppm. **MS** (negative and

positive ESI-MS, MeOH, acidified): *m/z* (%) = 337.1839 (50, [M-H$^+$], $C_{19}H_{29}O_3S^-$, calcd. 337.1837; 361.1810 (100, [M+Na$^+$], $C_{19}H_{30}O_3SNa^+$, calcd. 361.1813). **IR** (KBr): $\tilde{\nu}$ (cm^{-1}) = 3357, 3257, 2956, 2915, 2849, 2170, 2030, 1902, 1841, 1686, 1624, 1591, 1459, 1382, 1265, 1088, 1027, 854, 789, 699, 663. **Elemental Analysis**: $C_{19}H_{30}O_3S$: calcd. C = 67.42 %, H = 8.93 %; found C = 67.65 %, H = 8.91 %.

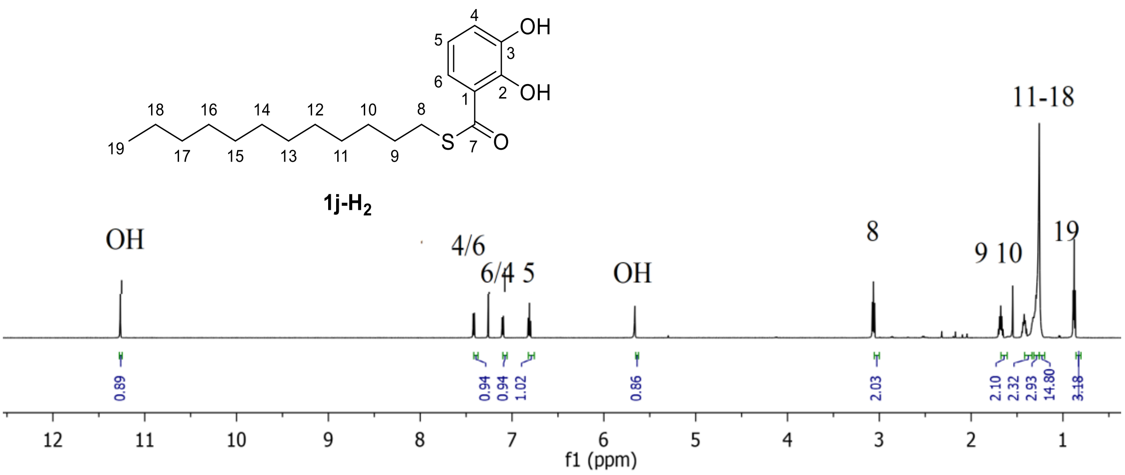

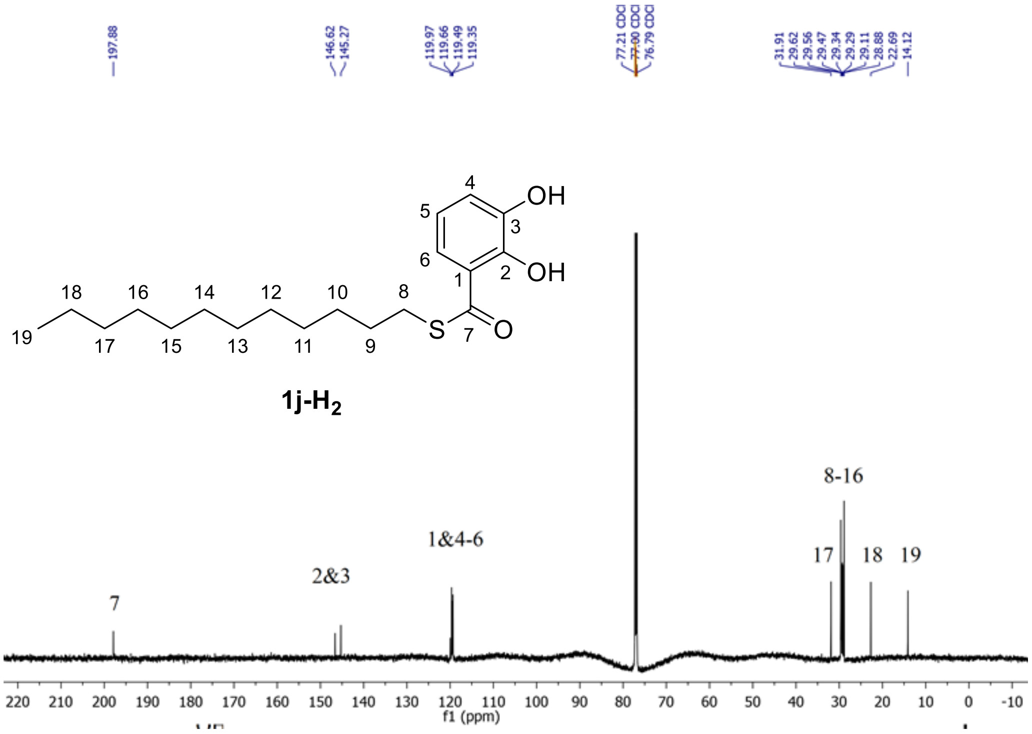

Isopropyl-2,3-dihydroxybenzothioate (1k-H$_2$):

The ligand is synthesized with 2-propanethiol (0.10 mL, 1.1 mmol) according to the general procedure. Column chromatography (pentane/ethyl acetate 4:1, R_f = 0.18) results in the product as a yellowish oil (62 %, 144 mg, 0.68 mmol). **^{1}H NMR** (400 MHz, $CDCl_3$): δ = 11.29 (s, 1H, OH), 7.38 (dd, *J* =

8.0, 1.5 Hz, 1H, $H_{arom.}$), 7.08 (dd, *J* = 8.0, 1.5 Hz, 1H, $H_{arom.}$), 6.78 (t, *J* = 8.0 Hz, 1H, $H_{arom.}$), 5.66 (s, 1H, OH), 3.95-3.88 (m, 1H, SCH), 1.40 (d, *J* = 6.9 Hz, 6H, 2×CH_3) ppm. **^{13}C NMR** (101 MHz, $CDCl_3$): δ = 197.97 (*C*OSCH$_2$), 146.70 (C_{arom}), 145.26 (C_{arom}), 119.99 (C_{arom}), 119.62 (C_{arom}), 119.42 (C_{arom}), 119.28 (C_{arom}), 35.02 (SCH), 22.91 (2×CH_3) ppm. **MS** (negative ESI-MS, MeOH, acidified): *m/z* (%) = 211.0444 (100, [M-H$^+$], $C_{10}H_{11}O_3S^-$, calcd. 211.0429). **IR** (KBr): ṽ (cm^{-1}) = 3899, 3470, 3062, 2969, 2926, 2868, 2727, 2484, 2250, 2059, 1906, 1843, 1781, 1627, 1596, 1456, 1368, 1264, 1162, 1029, 931, 856, 792, 731, 664, 579, 546, 481. **Elemental Analysis**: $C_{10}H_{12}O_3S$: calcd. C = 56.59 %, H = 5.70 %; found C = 56.56 %, H = 6.04 %.

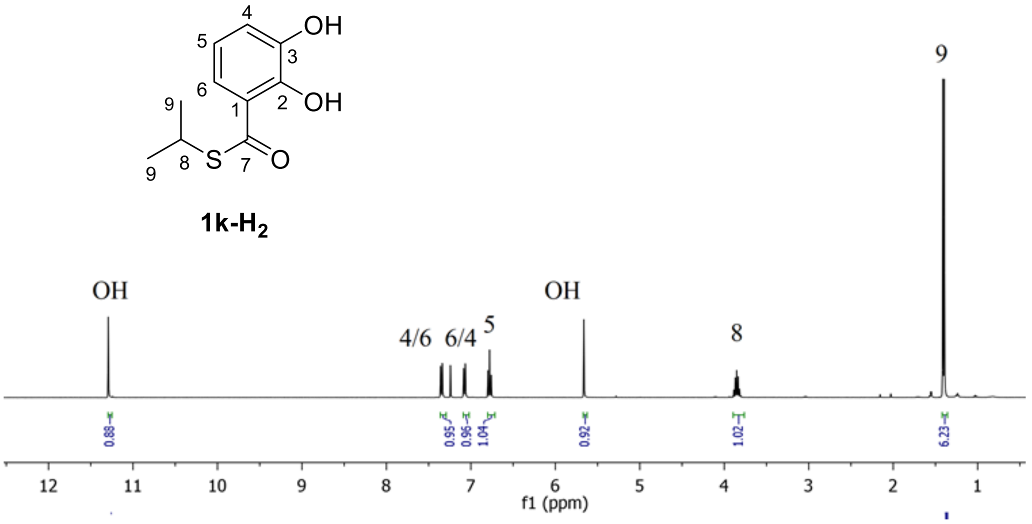

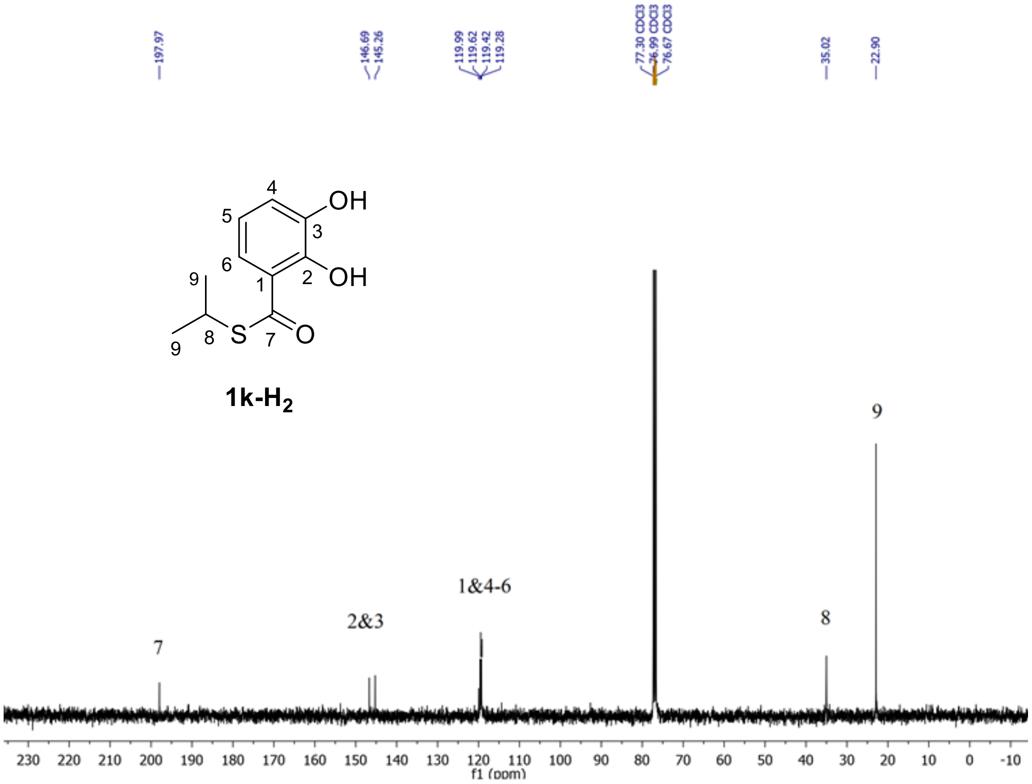

2-Methyl-1-Propanyl-2,3-dihydroxybenzothioate (1l-H_2):

The ligand is synthesized with 2-methyl-1-propanethiol (0.12 mL, 1.1 mmol) according to the general procedure. Column chromatography (DCM, R_f = 0.19) results in the product as a yellowish oil (51 %, 126 mg, 0.56 mmol). **^{1}H NMR** (600 MHz, $CDCl_3$): δ = 11.26 (s, 1H, OH), 7.42 (dd, *J* = 8.0, 1.4 Hz, 1H, $H_{arom.}$), 7.10 (dd, *J* = 8.0, 1.4 Hz, 1H, $H_{arom.}$), 6.82 (t, *J* = 8.0 Hz, 1H, $H_{arom.}$), 5.69 (s, 1H, OH), 3.00 (d, *J* = 7.5 Hz, 2H, SCH_2), 1.94-1.92 (m, 1H, CH), 1.14 (d, *J* = 7.0 Hz, 6H, 2×CH_3) ppm. **^{13}C NMR** (151 MHz, $CDCl_3$): δ = 197.73 (*C*OSCH_2), 146.63 (C_{arom}), 145.28 (C_{arom}), 119.98 (C_{arom}), 119.68 (C_{arom}), 119.53 (C_{arom}), 119.36 (C_{arom}), 37.06 (CH_2CH_2), 28.63 (SCH_2), 21.84 (2×CH_3) ppm. **MS** (negative and positive ESI-MS, MeOH, acidified): *m/z* (%) = 225.0582 (70, [M-H$^+$], $C_{11}H_{13}O_3S^-$, calcd. 225.0585; 249.0563 (20, [M+Na$^+$], $C_{11}H_{14}O_3SNa^+$, calcd. 249.0561). **IR** (KBr): ṽ (cm^{-1}) = 3465, 3062, 2960, 2872, 2328, 2119, 1901, 1627, 1596, 1455, 1366, 1256, 1166, 1079, 1025, 853, 790, 728, 659. **Elemental Analysis**: $C_{11}H_{14}O_3S$: calcd. C = 58.39 %, H = 6.24 %; found C = 58.46 %, H = 6.19 %.

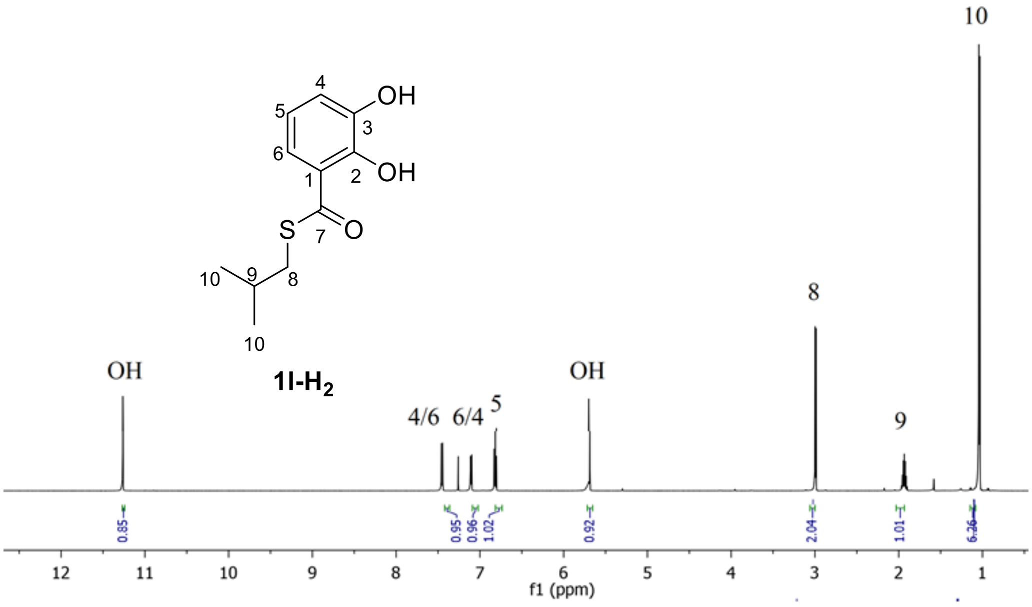

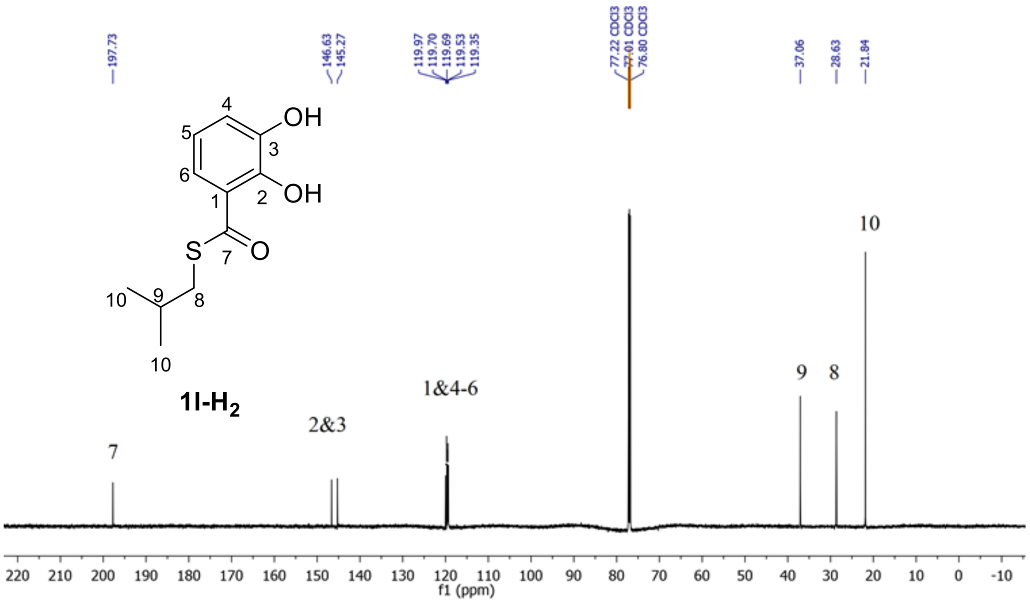

Cyclopentanyl-2,3-dihydroxybenzothioate (1m-H_2):

The ligand is synthesized with cyclopentanethiol (112.42 mg, 1.1 mmol) according to the general procedure. Column chromatography (DCM, R_f = 0.24) results in the product as a yellowish solid (55 %, 144 mg, 0.61 mmol). **M.p.**: 49 °C – 51 °C (last solvent used: DCM). **^{1}H NMR** (400 MHz, $CDCl_3$): δ = 11.31 (s, 1H, OH), 7.35 (dd, *J* = 8.0, 1.5 Hz, 1H, $H_{arom.}$), 7.07 (dd, *J* = 8.0, 1.5 Hz, 1H, $H_{arom.}$), 6.78 (t, *J* = 8.0 Hz, 1H, $H_{arom.}$), 5.65 (s, 1H, OH), 3.91-3.89 (m, 1H, SCH), 2.20-2.18 (m, 2H, $CH_{2cypent}$), 1.68-1.48 (m, 6H, 3×$CH_{2cypent}$) ppm. **^{13}C NMR** (101 MHz, $CDCl_3$): δ = 198.59 (*C*OSCH_2), 146.64 (C_{arom}), 145.25 (C_{arom}), 119.99 (C_{arom}), 119.57 (C_{arom}), 119.51 (C_{arom}), 119.27 (C_{arom}), 42.56 (SCH), 33.14 (2×C_{cypent}), 24.75 (2×C_{cyhex}) ppm. **MS** (negative and positive ESI-MS, MeOH, acidified): *m/z* (%) = 237.0591 (100, [M-H^+], $C_{12}H_{13}O_3S^-$, calcd. 237.0585; 261.0555 (20, [M+Na^+], $C_{12}H_{14}O_3SNa^+$, calcd. 261.0561). **IR** (KBr): ṽ (cm^{-1}) = 3337, 2954, 2867, 2661, 2328, 2178, 2050, 1883, 1824, 1626, 1590, 1482, 1453, 1375, 1205, 1086, 1020, 848, 787,749, 712, 660. **Elemental Analysis**: $C_{12}H_{14}O_3S$: calcd. C = 60.48 %, H = 5.92 %; found C = 60.30 %, H = 7.85 %.

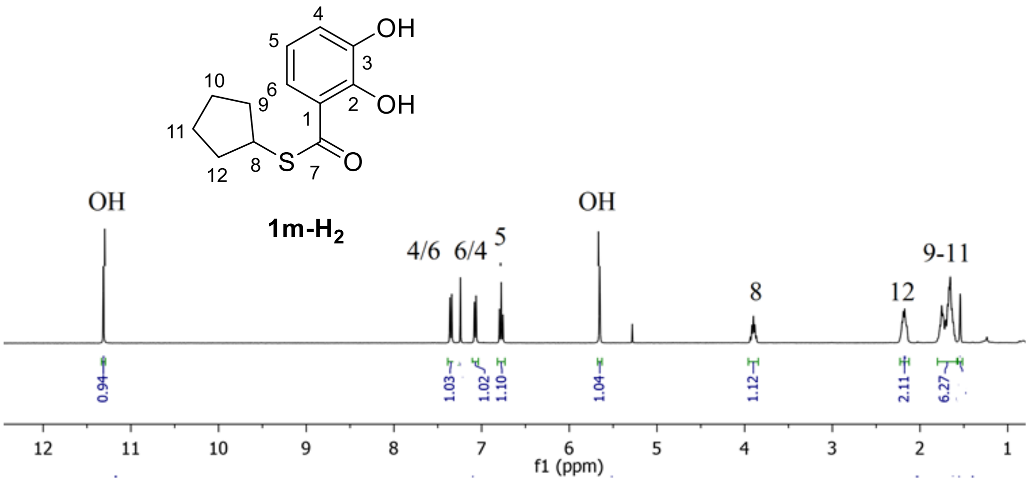

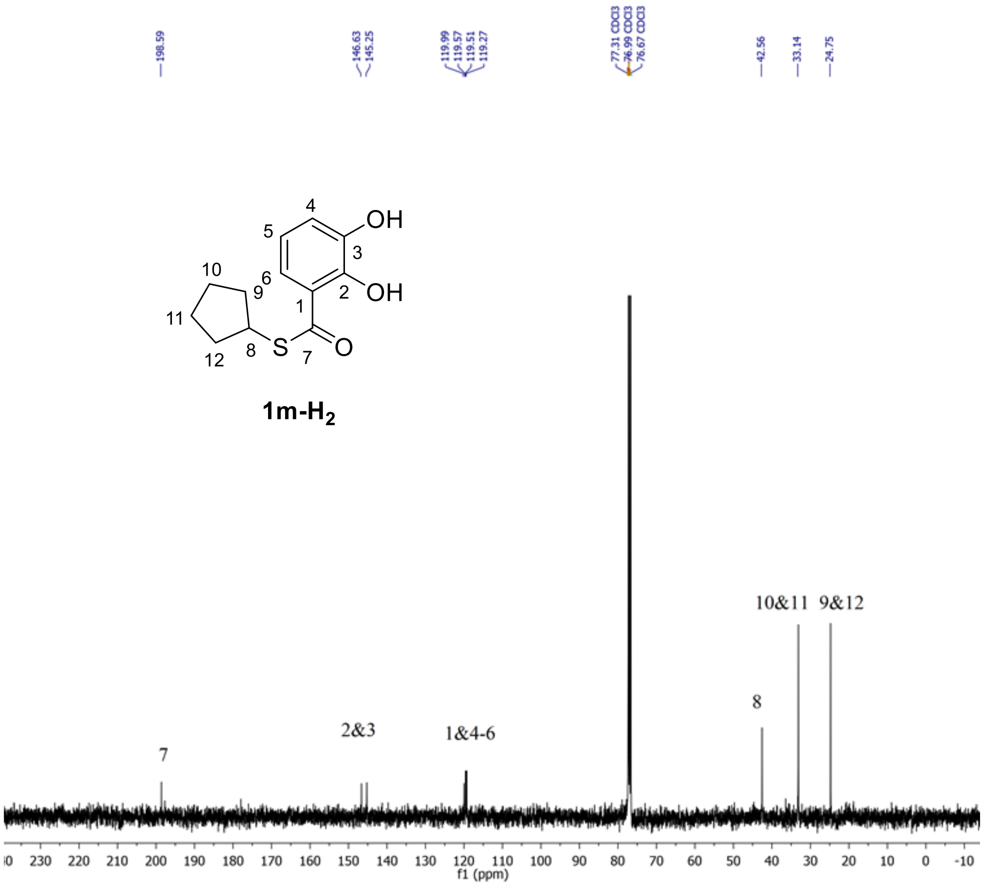

Cyclohexanyl-2,3-dihydroxybenzothioate (1n-H_2):

The ligand is synthesized with cyclohexanethiol (0.13 mL, 1.1 mmol) according to the general procedure. Column chromatography (DCM, R_f = 0.17) results in the product as a yellowish oil (47 %, 130 mg, 0.52 mmol). **^{1}H NMR** (600 MHz, $CDCl_3$): δ = 11.32 (s, 1H, OH), 7.43 (dd, *J* = 8.0, 1.4 Hz, 1H, $H_{arom.}$), 7.09 (dd, *J* = 8.0, 1.4 Hz, 1H, $H_{arom.}$), 6.79 (t, *J* = 8.0 Hz, 1H, $H_{arom.}$), 5.67 (s, 1H, OH), 3.75-3.73 (m, 1H, SCH), 2.03-2.01 (m, 2H, CH_{2cyhex}), 1.77-1.75 (m, 2H, CH_{2cyhex}), 1.58-1.43 (m, 4H, 2×CH_{2cyhex}), 1.39-

1.37 (m, 2H, CH_{2cyhex}) ppm. **^{13}C NMR** (151 MHz, $CDCl_3$): δ = 197.76 (*C*$OSCH_2$), 146.74 (C_{arom}), 145.27 (C_{arom}), 120.04 (C_{arom}), 119.60 (C_{arom}), 119.47 (C_{arom}), 119.27 (C_{arom}), 42.62 (SCH), 32.94 (2×C_{cyhex}), 25.95 (C_{cyhex}), 25.52 (2×C_{cyhex}) ppm. **MS** (negative and positive ESI-MS, MeOH, acidified): *m/z* (%) = 251.0729 (100, [M-H$^+$], $C_{13}H_{15}O_3S^-$, calcd. 251.0742; 275.0702 (100, [M+Na$^+$], $C_{13}H_{16}O_3SNa^+$, calcd. 275.0718). **IR** (KBr): $\tilde{\nu}$ (cm^{-1}) = 3485, 3255, 2936, 2854, 2475, 2323, 2157, 2070, 2027, 1908, 1841, 1776, 1624, 1586, 1526, 1487, 1448, 1324, 1266, 1224, 1198, 1169, 1081, 1024, 854, 786, 729, 661. **Elemental Analysis**: $C_{13}H_{16}O_3S$: calcd. C = 61.88 %, H = 6.39 %; found C = 61.77 %, H = 6.52 %.

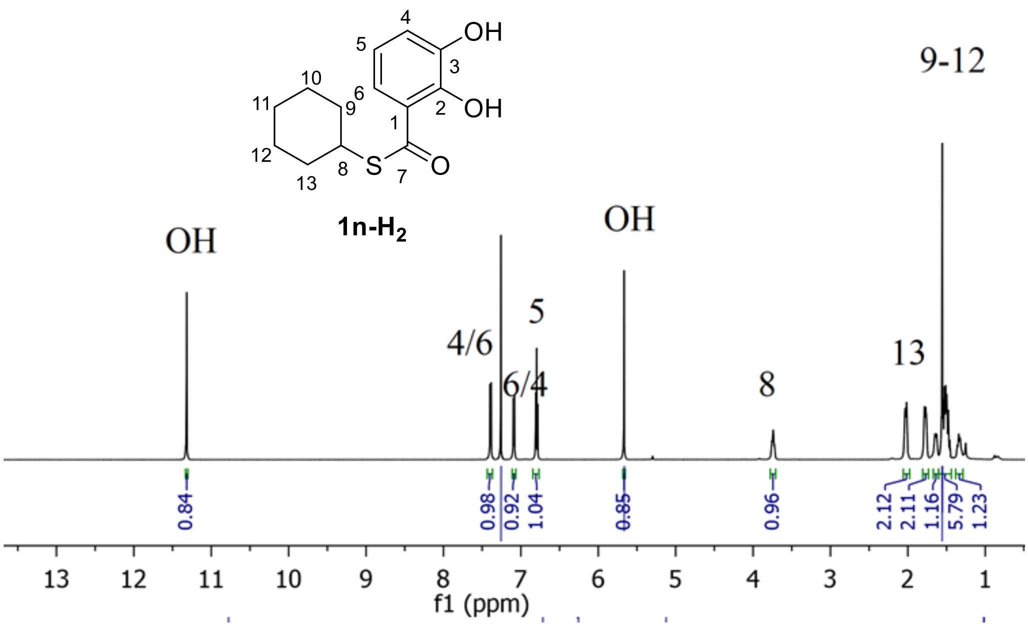

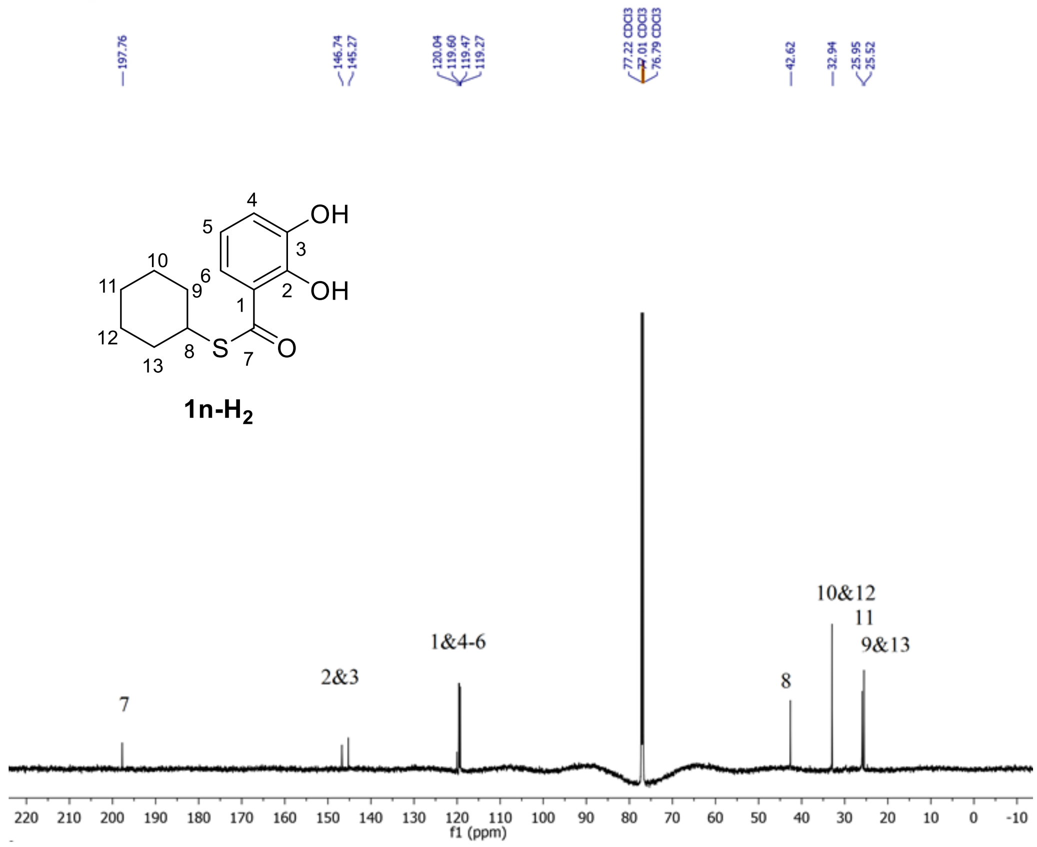

Phenyl-2,3-dihydroxybenzothioate (1o-H$_2$):

The ligand is synthesized with thiophenol (0.40 mL, 3.89 mmol) according to the general procedure. Column chromatography (pentane/ ethyl acetate 8:1, R_f = 0.25) results in the product as a white solid (41 %, 130 mg, 0.53 mmol). **M.p.**: 50 °C – 51 °C (last solvent used: DCM). **^{1}H NMR** (400 MHz, $CDCl_3$, a/Kwamen_CK704): δ = 10.89 (s, 1H, OH), 7.43-7.46 (m, 6H, $H_{arom.}$), 7.14 (dd, *J* = 7.9, 1.5 Hz, 1H, H_{arom}), 6.87 (t, *J* = 7.9 Hz, 1H, H_{arom}), 5.64 (s, 1H, OH) ppm. **MS** (negative ESI-MS, MeOH): *m/z* (%) = 245.0283 (100, $[M-H^+]$, $C_{13}H_9O_3S^-$, calcd. 245.0272). **Elemental Analysis**: $C_{13}H_{10}O_3S \cdot 1/2\ H_2O$: calcd. C = 61.16 %, H = 4.34 %; found C = 61.53 %, H = 4.67 %.

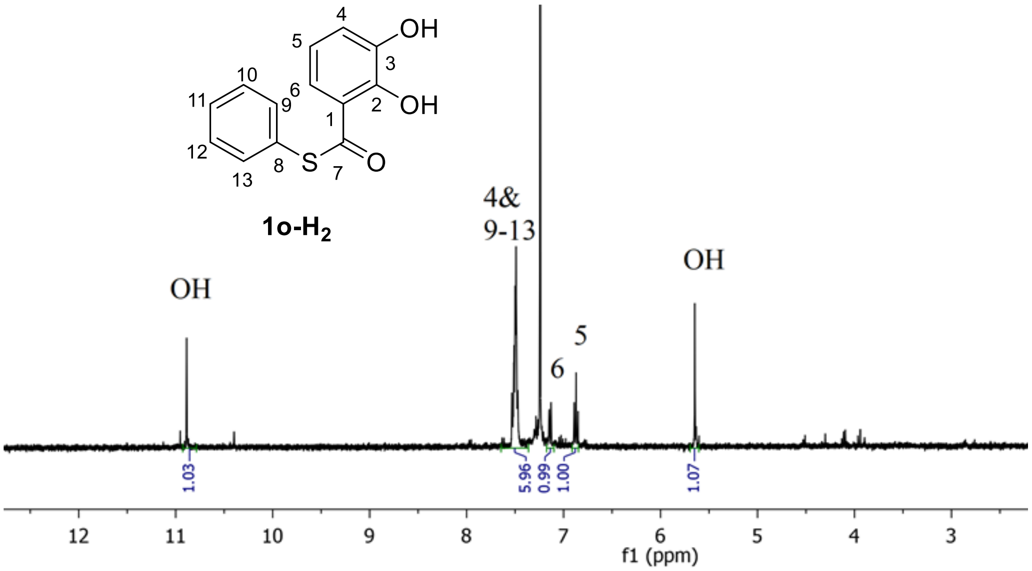

Benzyl-2,3-dihydroxybenzothioate (1p-H_2):

The ligand is synthesized with benzyl mercaptan (136.62 mg, 1.1 mmol) according to the general procedure. Column chromatography (pentane/ ethyl acetate 20:1, R_f = 0.3) results in the product as a yellowish solid (47 %, 130 mg, 0.52 mmol). **M.p.**: 75 °C – 77 °C (last solvent used: DCM). **^{1}H NMR** (600 MHz, $CDCl_3$): δ = 11.15 (s, 1H, OH), 7.39-7.37 (m, 3H, $H_{arom.}$), 7.34-7.32 (m, 2H, H_{arom}), 7.28 (dd, *J* = 7.8, 1.5 Hz, 1H, H_{arom}), 7.10 (dd, *J* = 7.8, 1.5 Hz, 1H, $H_{arom.}$), 6.80 (t, *J* = 7.8 Hz, 1H, $H_{arom.}$), 5.69 (s, 1H, OH), 4.32 (s, 2H, SCH_2) ppm. **^{13}C NMR** (151 MHz, $CDCl_3$): δ = 196.84 (*C*OSCH_2), 146.70 (C_{arom}), 145.31 (C_{arom}), 136.61 (C_{arom}), 128.98 (2×C_{arom}), 128.74 (2×C_{arom}), 127.59 (C_{arom}), 119.96 (2×C_{arom}), 119.50 (2×C_{arom}), 33.11 (SCH_2) ppm. **MS** (negative and positive ESI-MS, MeOH, acidified): *m/z* (%) = 259.0473 (100, [M-H^+], $C_{14}H_{11}O_3S^-$, calcd. 259.0429; 283.0395 (50, [M+Na^+], $C_{14}H_{12}O_3SNa^+$, calcd. 283.0405). **IR** (KBr): $\tilde{\nu}$ (cm^{-1}) = 3867, 3642, 3453, 3062, 2927, 2719, 2311, 2164, 2074, 2000, 1907, 1757, 1626, 1453, 1355, 1240, 1074, 1019, 911, 848, 785, 697. **Elemental Analysis**: $C_{14}H_{12}O_3S$: calcd. C = 64.60 %, H = 4.65 %; found C = 64.81 %, H = 4.90 %.

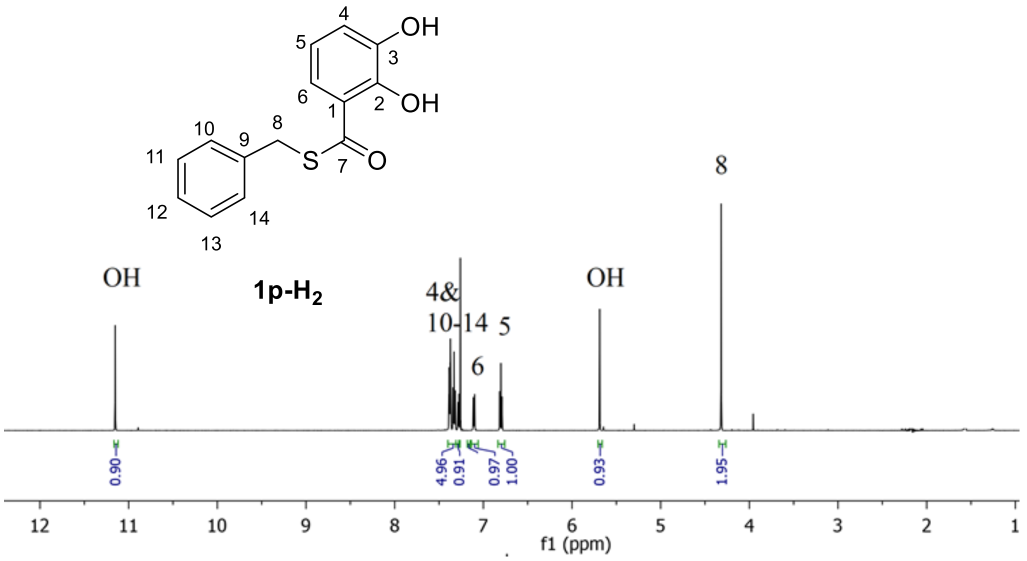

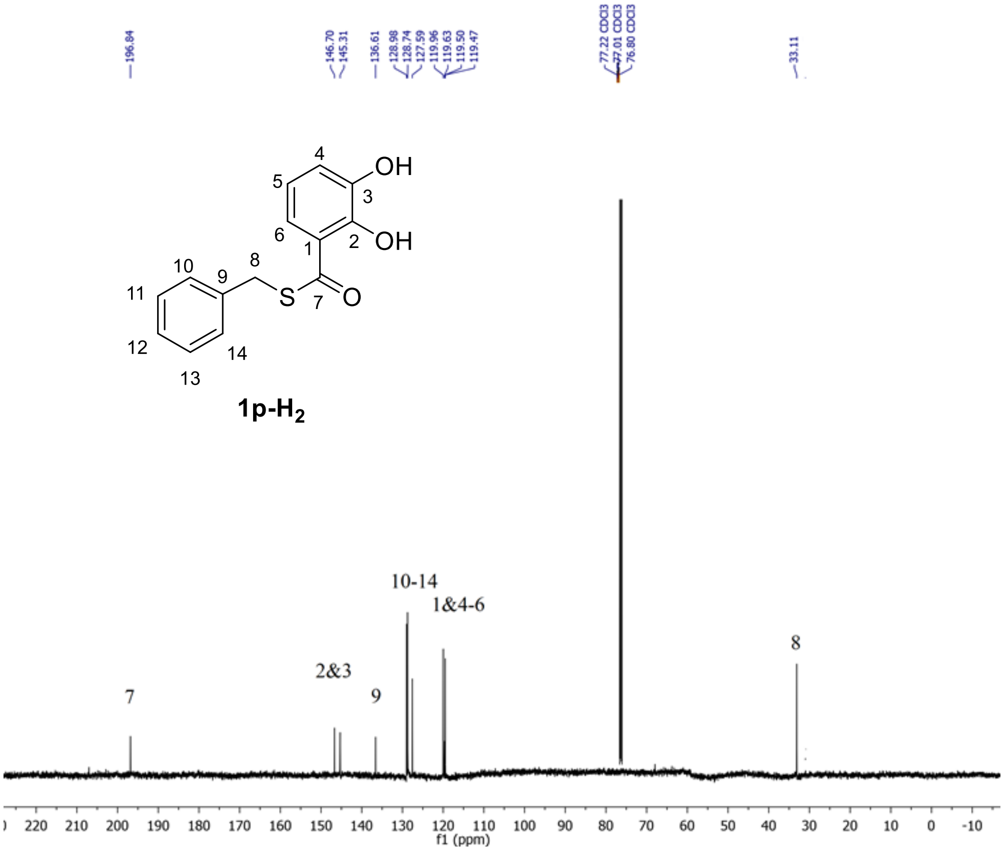

Complexes Li[Li$_3$(1 (a-p)$_3$Ti)$_2$]

Li[Li$_3$(1a$_3$Ti)$_2$]:

Ligand **1a**-H$_2$ (50 mg, 0.27 mmol) is converted into the corresponding complex in methanol (50mL). The product is obtained after removal of the solvent under reduced pressure as red solid (quantitative). **^{1}H NMR** (600 MHz, MeOH-d$_4$): Dimer (major component): δ = 7.24-7.21 (m, 1H, H$_{arom.}$), 6.59-6.57 (m, 2H, H$_{arom}$), 2.00 (s, 3H, SCH$_3$) ppm. Monomer (minor component): δ = 7.12-7.10 (m, 1H,

H_{arom}), 2.15 (s, 3H, SCH_3) ppm. Signals not listed are overlapping and cannot be assigned. **^{1}H NMR** (600 MHz, DMSO-d_6): Only monomer: δ = 6.76 (dd, J = 7.9, 1.5 Hz, 1H, H_{arom}), 6.28 (t, J = 7.9 Hz, 1H, H_{arom}), 6.13 (dd, J = 7.9, 1.5 Hz, 1H, H_{arom}), 2.15 (s, 3H, SCH_3) ppm. **MS** (negative and positive ESI-MS, MeOH, acidified): m/z (%) = 1208.9661 (100, [M_D-Li^+], $C_{48}H_{36}O_{18}S_6Li_3Ti_2^-$, calcd. 1208.9664), 600.9721 (98, [M_M-Li^+], $C_{24}H_{18}O_9S_3LiTi^-$, calcd. 600.9752); 1223.0001 (40, [M_D+Li^+], $C_{48}H_{36}O_{18}S_6Li_5Ti_2^+$, calcd. 1222.9984), 615.0062 (100, [M_M+Li^+], $C_{24}H_{18}O_9S_3Li_3Ti^+$, calcd. 615.0072). **IR (KBr)**: $\tilde{\nu}$ (cm^{-1}) = 3628, 3190, 3058, 2926, 2850, 2651, 2321, 2178, 2064, 1993, 1954, 1741, 1689, 1621, 1552, 1431, 1304, 1251, 1210, 1169, 1091, 1049, 964, 862, 799, 726, 690. **Elemental analysis** $C_{48}H_{36}O_{18}S_6Li_4Ti_2 \cdot 5\ H_2O$: calcd. C = 44.12 %, H = 3.55 %; found C = 44.20 %, H = 4.22 %.

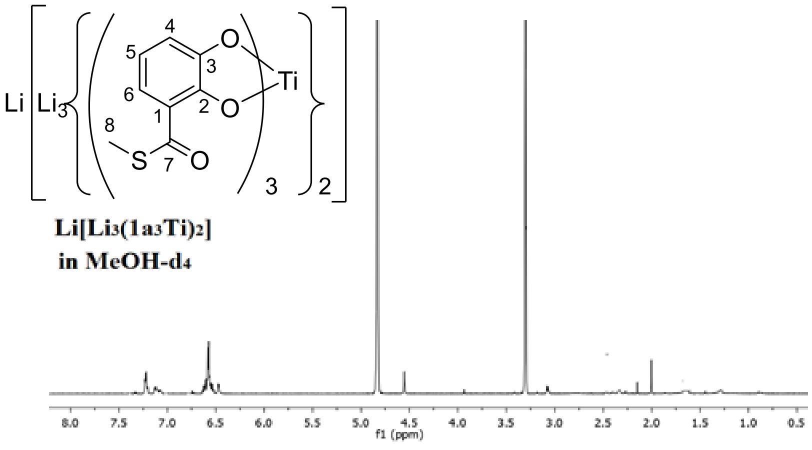

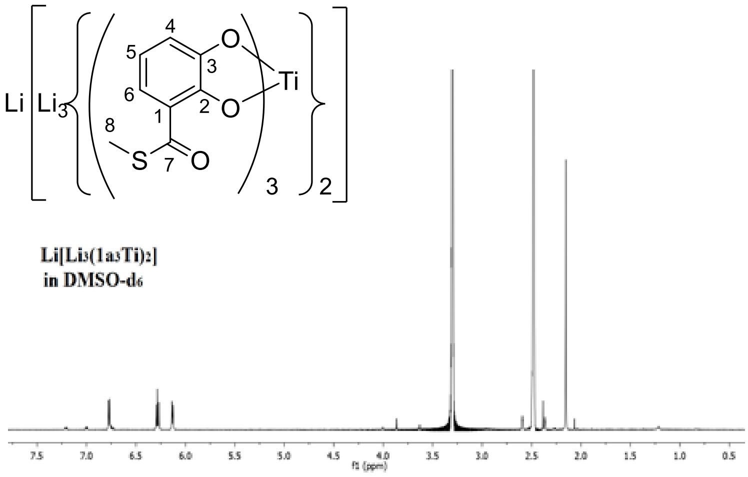

Li[Li_3($1b_3$Ti)$_2$]:

Ligand **1b**-H_2 (50 mg, 0.25 mmol) is converted into the corresponding complex in methanol (50mL). The product is obtained after removal of the solvent under reduced pressure as red solid (quantitative). **^{1}H NMR** (400 MHz, MeOH-d_4): Dimer (major component): δ = 7.15 (dd, J = 7.8, 1.5 Hz, 1H, H_{arom}), 2.38-2.36 (m, 1H, SCH_2), 2.03-2.01 (m, 1H, SCH_2), 0.87 (t, J = 7.5 Hz, 3H, CH_2CH_3) ppm. Monomer (minor component): δ = 7.08 (dd, J = 7.8, 1.5 Hz, 1H, H_{arom}), 6.48 (dd, J = 7.8, 1.5 Hz, 1H,

H_{arom}), 2.96 (q, *J* =7.5 Hz, 2H, SCH_2) 1.28 (t, *J* = 7.5 Hz, 3H, CH_2CH_3) ppm. Signals not listed are overlapping and cannot be assigned. **^{1}H NMR** (400 MHz, DMSO-d$_6$): Only monomer: δ = 6.77 (dd, *J* = 7.9, 1.5 Hz, 1H, H_{arom}), 6.28 (t, *J* = 7.9 Hz, 1H, H_{arom}), 6.13 (dd, *J* = 7.9, 1.6 Hz, 1H, H_{arom}), 2.76 (q, *J* = 7.4 Hz, 2H, SCH_2), 1.16 (t, *J* = 7.4 Hz, 3H, CH_2CH_3) ppm. **MS** (negative ESI-MS, MeOH, acidified): *m/z* (%) = 1293.0592 (70, [M_D-Li^+], $C_{54}H_{48}O_{18}S_6Li_3Ti_2^-$, calcd. 1293.0604), 643.0197 (100, [M_M-Li^+], $C_{27}H_{24}O_9S_3LiTi^-$, calcd. 643.0222). **IR (KBr)**: $\tilde{\nu}$ (cm^{-1}) = 3629, 3374, 3060, 2928, 2868, 2656, 2500, 2332, 2163, 2113, 2074, 2029, 1961, 1880, 1742, 1618, 1552, 1429, 1300, 1249, 1207, 1093, 1044, 968, 862, 797, 688. **Elemental analysis** $C_{54}H_{48}O_{18}S_6Li_4Ti_2 \cdot 4\ H_2O$: calcd. C = 47.24 %, H = 4.11 %; found C = 47.27 %, H = 4.18 %.

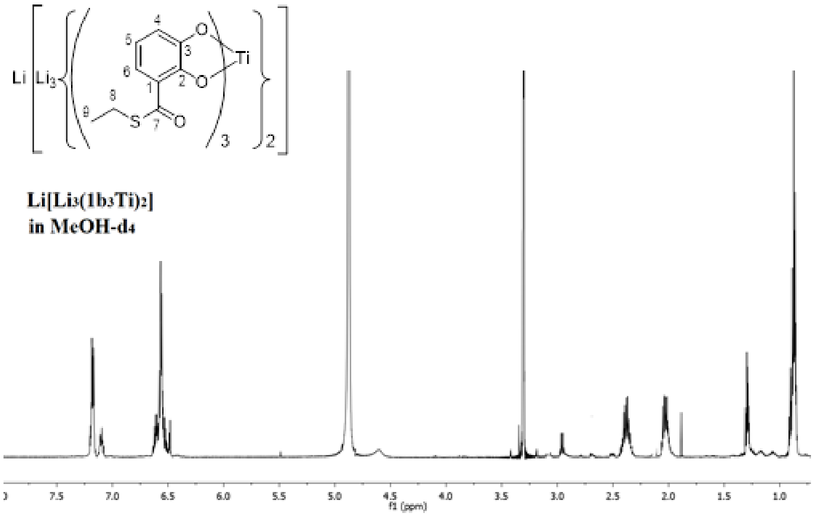

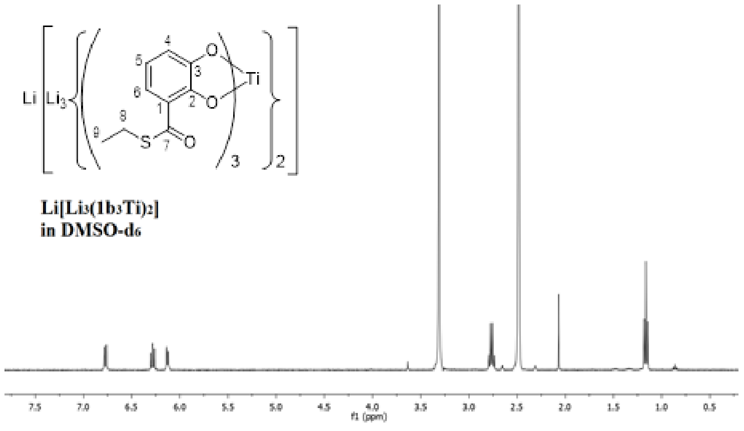

Li[Li$_3$(1c$_3$Ti)$_2$]:

Ligand **1c**-H_2 (50 mg, 0.24 mmol) is converted into the corresponding complex in methanol (50mL). The product is obtained after removal of the solvent under reduced pressure as red solid (quantitative). **^{1}H NMR** (400 MHz, MeOH-d$_4$): Dimer (major component): δ = 7.18 (dd, *J* = 7.9, 1.4 Hz, 1H, H_{arom}), 2.47-2.45 (m, 1H, SCH_2), 1.97-1.95 (m, 1H, SCH_2), 1.15-1.13 (m, 2H, CH_2CH_3), 0.89 (t, *J* = 7.5

Hz, 3H, CH_2CH_3) ppm. Monomer (minor component): δ = 7.09 (dd, J = 7.9, 1.5 Hz, 1H, H_{arom}), 6.48 (dd, J = 7.9, 1.5 Hz, 1H, H_{arom}), 2.94 (t, J =7.7 Hz, 2H, SCH_2), 1.66-1.64 (m, 2H, CH_2CH_3), 0.98 (t, J = 7.7 Hz, 3H, CH_2CH_3) ppm. Signals not listed are overlapping and cannot be assigned. **^{1}H NMR** (400 MHz, DMSO-d_6): Only monomer: δ = 6.77 (dd, J = 7.9, 1.5 Hz, 1H, H_{arom}), 6.28 (t, J = 7.9 Hz, 1H, H_{arom}), 6.13 (dd, J = 7.9, 1.5 Hz, 1H, H_{arom}), 2.75 (t, J = 7.5 Hz, 2H, SCH_2), 1.53-1.51 (m, 2H, CH_2CH_3), 0.92 (t, J = 7.4 Hz, 3H, CH_2CH_3) ppm. **MS** (negative and positive ESI-MS, MeOH, acidified): *m/z* (%) = 1377.1571 (100, [M_D-Li^+], $C_{60}H_{60}O_{18}S_6Li_3Ti_2^-$, calcd. 1377.1542), 685.0684 (99, [M_M-Li^+], $C_{30}H_{30}O_9S_3LiTi^-$, calcd. 685.0691); 1391.1886 (10, [M_D+Li^+], $C_{60}H_{60}O_{18}S_6Li_5Ti_2^+$, calcd. 1391.1862), 699.1011 (20, [M_M+Li^+], $C_{30}H_{30}O_9S_3Li_3Ti^+$, calcd. 699.1011). **IR (KBr)**: $\tilde{\nu}$ (cm^{-1}) = 3643, 3419, 3060, 2961, 2866, 2669, 2322, 2176, 2085, 1884, 1744, 1622, 1549, 1431, 1251, 1210, 1091, 1045, 948, 863, 798, 728, 692. **Elemental analysis** $C_{60}H_{60}O_{18}S_6Li_4Ti_2 \cdot 4\ H_2O$: calcd. C = 49.46 %, H = 4.70 %; found C = 49.84 %, H = 4.62 %.

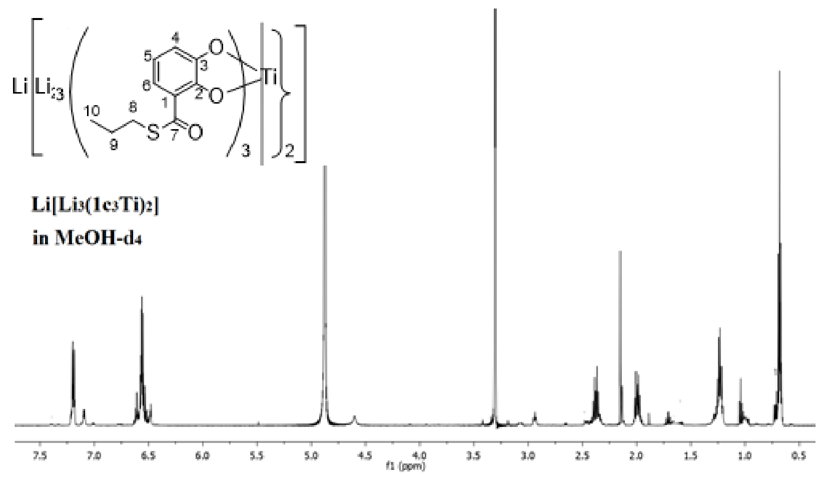

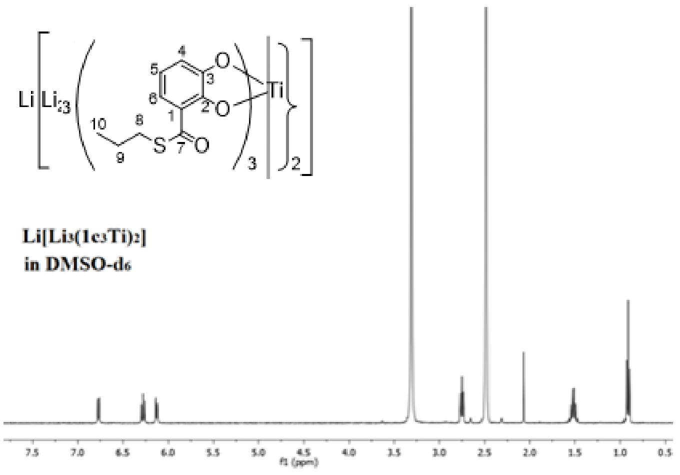

Li[Li$_3$(1d$_3$Ti)$_2$]:

Ligand **1d**-H$_2$ (50 mg, 0.22 mmol) is converted into the corresponding complex in methanol (50mL). The product is obtained after removal of the solvent under reduced pressure as red solid (quantitative). **^{1}H NMR** (400 MHz, MeOH-d$_4$): Dimer (major component): *δ* = 7.17 (dd, *J* = 7.8, 2.1 Hz, 1H, H$_{arom}$), 2.38-2.36 (m, 1H, SCH$_2$), 1.96-1.94 (m, 1H, SCH$_2$), 1.18-1.16 (m, 2H, CH$_2$CH$_2$), 1.03-1.01 (m, 2H, CH$_2$CH$_2$), 0.65 (t, *J* = 7.4 Hz, 3H, CH$_2$CH$_3$) ppm. Monomer (minor component): *δ* = 7.08 (dd, *J* = 7.8, 1.5 Hz, 1H, H$_{arom}$), 2.95 (t, *J* = 7.5 Hz, 2H, SCH$_2$), 1.62-1.60 (m, 2H, CH$_2$CH$_2$), 1.46-1.44 (m, 2H, CH$_2$CH$_2$), 0.95 (t, *J* = 7.4 Hz, 3H, CH$_2$CH$_3$) ppm. Signals not listed are overlapping and cannot be assigned. **^{1}H NMR** (400 MHz, DMSO-d$_6$): Only monomer: *δ* = 6.77 (dd, *J* = 8.0, 1.6 Hz, 1H, H$_{arom}$), 6.28 (t, *J* = 8.0 Hz, 1H, H$_{arom}$), 6.13 (dd, *J* = 8.0, 1.6 Hz, 1H, H$_{arom}$), 2.77 (t, *J* = 6.6 Hz, 2H, SCH$_2$), 1.49-1.47 (m, 2H, CH$_2$CH$_2$), 1.35-1.33 (m, 2H, CH$_2$CH$_2$), 0.86 (t, *J* = 7.3 Hz, 3H, CH$_2$CH$_3$) ppm. **MS** (negative ESI-MS, MeOH, acidified): *m/z* (%) = 1461.2460 (100, [M$_D$-Li$^+$], $C_{66}H_{72}O_{18}S_6Li_3Ti_2^-$, calcd. 1461.2482), 727.1140 (97, [M$_M$-Li$^+$], $C_{33}H_{36}O_9S_3LiTi^-$, calcd. 727.1161). **IR (KBr)**: $\tilde{\nu}$ (cm^{-1}) = 3605, 3414, 3209, 3058, 2929, 2864, 2652, 2496, 2323, 2108, 2024, 1980, 1915, 1745, 1618, 1551, 1426, 1302, 1248, 1204, 1092, 1044, 957, 863, 793, 731, 684. **Elemental analysis** $C_{66}H_{72}O_{18}S_6Li_4Ti_2 \cdot 4\ H_2O$: calcd. C = 51.44 %, H = 5.23 %; found C = 51.10 %, H = 5.23 %.

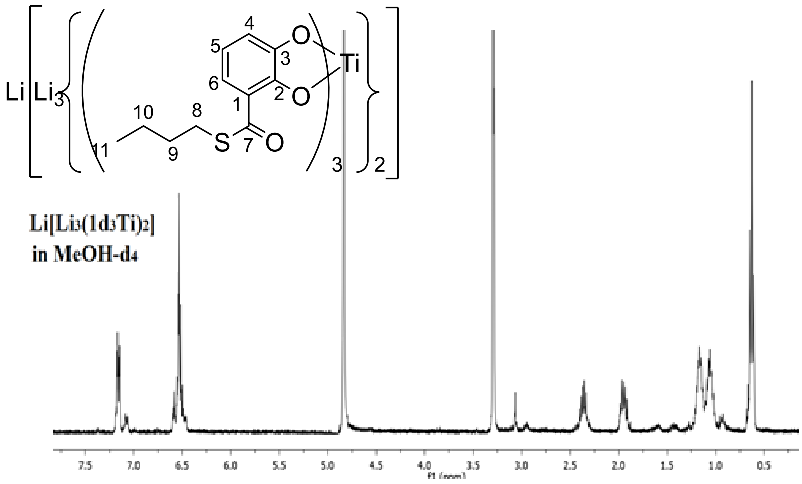

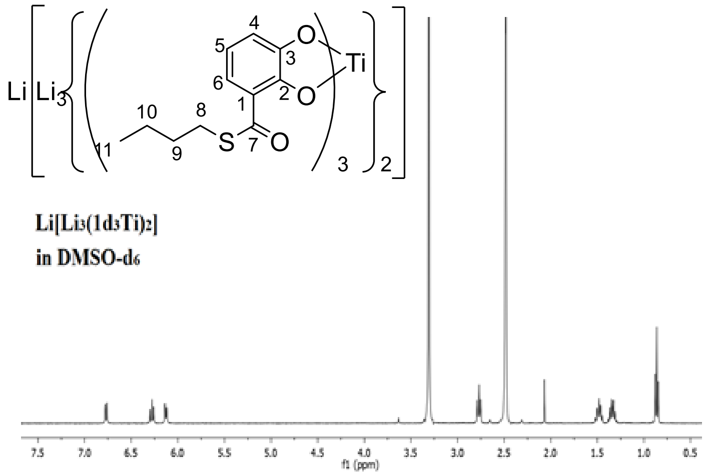

Li[Li_3(1e_3Ti)$_2$]:

Ligand **1e**-H_2 (50 mg, 0.21 mmol) is converted into the corresponding complex in methanol (50mL). The product is obtained after removal of the solvent under reduced pressure as red solid (quantitative). **^{1}H NMR** (400 MHz, MeOH-d_4): Dimer (major component): δ = 7.15 (dd, J = 7.8, 1.5 Hz, 1H, H_{arom}), 2.35-2.33 (m, 1H, SCH_2), 1.88-1.86 (m, 1H, SCH_2), 1.18-1.15 (m, 2H, CH_2CH_2), 1.10-0.98 (m, 4H, 2×CH_2), 0.63 (t, J = 6.7 Hz, 3H, CH_2CH_3) ppm. Monomer (minor component): δ = 7.07 (dd, J = 7.8, 1.5 Hz, 1H, H_{arom}), 2.95 (t, J = 7.3 Hz, 2H, SCH_2), 1.66-1.64 (m, 2H, CH_2CH_2), 1.37-1.35 (m, 4H, 2×CH_2), 0.92 (t, J = 6.8 Hz, 3H, CH_2CH_3) ppm. Signals not listed are overlapping and cannot be assigned. **^{1}H NMR** (400 MHz, DMSO-d_6): Only monomer: δ = 6.78 (dd, J = 7.9, 1.5 Hz, 1H, H_{arom}), 6.29 (t, J = 7.9 Hz, 1H, H_{arom}), 6.14 (dd, J = 7.9, 1.5 Hz, 1H, H_{arom}), 2.77 (t, J = 7.3 Hz, 2H, SCH_2), 1.51-1.49 (m, 2H, CH_2CH_2), 1.35-1.24 (m, 4H, 2×CH_2), 0.85 (t, J = 7.0 Hz, 3H, CH_2CH_3) ppm. **MS** (negative and positive ESI-MS, MeOH): *m/z* (%) = 1545.34314 (100, [M_D-Li^+], $C_{72}H_{84}O_{18}S_6Li_3Ti_2^-$, calcd. 1545.3422), 769.1622 (10, [M_M-Li^+], $C_{36}H_{42}O_9S_3LiTi^-$, calcd. 769.1631); 1559.3761 (80, [M_D+Li^+], $C_{72}H_{84}O_{18}S_6Li_5Ti_2^+$, calcd. 1559.3742). **IR (KBr)**:$\tilde{\nu}$ (cm^{-1}) = 3390, 3060, 2925, 2859, 2676, 2492, 2322, 2234, 2184, 2029, 1882, 1620, 1551, 1430, 1250, 1208, 1168, 1090, 1046, 983, 862, 795, 727, 690. **Elemental analysis** $C_{72}H_{84}O_{18}S_6Li_4Ti_2 \cdot 3\ H_2O$: calcd. C = 53.80 %, H = 5.64 %; found C = 53.76 %, H = 5.50 %.

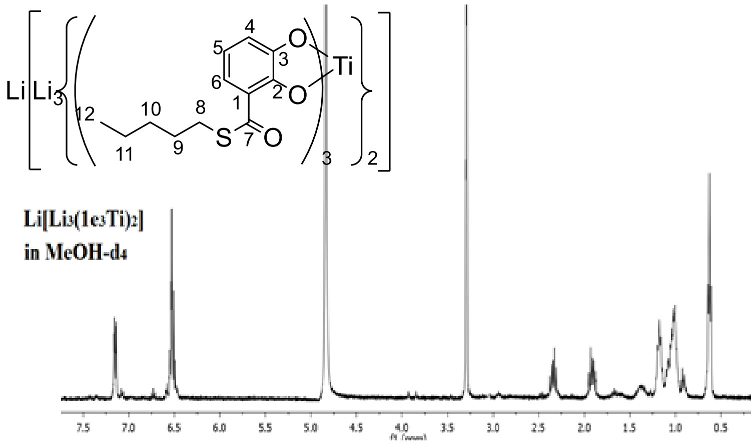

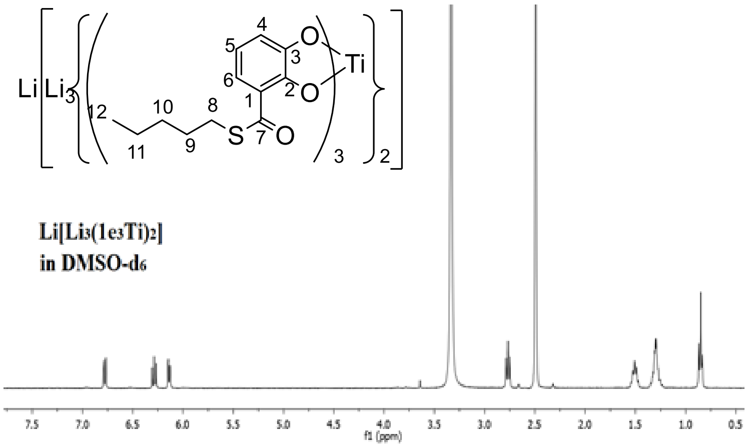

Li[Li$_3$(1f$_3$Ti)$_2$]:

Ligand **1f**-H$_2$ (50 mg, 0.20 mmol) is converted into the corresponding complex in methanol (50mL). The product is obtained after removal of the solvent under reduced pressure as red solid (quantitative). **^{1}H NMR** (400 MHz, MeOH-d$_4$): Dimer (major component): δ = 7.14 (dd, J = 7.9, 1.5 Hz, 1H, H$_{arom}$), 2.36-2.34 (m, 1H, SCH$_2$), 1.94-1.92 (m, 1H, SCH$_2$) ppm. Monomer (minor component): δ = 7.09 (dd, J = 7.9, 1.4 Hz, 1H, H$_{arom}$), 2.94 (t, J = 7.3 Hz, 2H, SCH$_2$), 1.66-1.64 (m, 2H, CH$_2$CH$_2$) ppm. Signals not listed are overlapping and cannot be assigned. **^{1}H NMR** (400 MHz, DMSO-d$_6$): Only monomer: δ = 6.77 (dd, J = 7.8, 1.6 Hz, 1H, H$_{arom}$), 6.28 (t, J = 7.8 Hz, 1H, H$_{arom}$), 6.14 (dd, J = 7.8, 1.6 Hz, 1H, H$_{arom}$), 2.77 (t, J = 7.4 Hz, 2H, SCH$_2$), 1.50-1.48 (m, 2H, CH$_2$CH$_2$), 1.39-1.10 (m, 6H, 3×CH$_2$), 0.85 (t, J = 6.9 Hz, 3H, CH$_2$CH$_3$) ppm. **MS** (negative and positive ESI-MS, MeOH): *m/z* (%) = 1629.4393 (100, [M$_D$-Li$^+$], $C_{78}H_{96}O_{18}S_6Li_3Ti_2^-$, calcd. 1629.4360), 811.2108 (20, [M$_M$-Li$^+$], $C_{39}H_{48}O_9S_3LiTi^-$, calcd. 811.2100); 1643.4697 (40, [M$_D$+Li$^+$], $C_{78}H_{96}O_{18}S_6Li_5Ti_2^+$, calcd. 1643.4680), 825.2523 (20, [M$_M$+Li$^+$], $C_{39}H_{48}O_9S_3Li_3Ti^+$, calcd. 825.2420). **IR (KBr)**: $\tilde{\nu}$ (cm^{-1}) = 3406, 3060, 2923, 2856, 2671, 2327, 2183, 2078, 1979, 1906, 1746, 1685, 1621, 1549, 1430, 1250, 1209, 1091, 1046, 980, 862, 797, 726, 690.

Elemental analysis $C_{78}H_{96}O_{18}S_6Li_4Ti_2 \cdot 4\ H_2O$: calcd. C = 54.80 %, H = 6.13 %; found C = 54.59 %, H = 5.90 %.

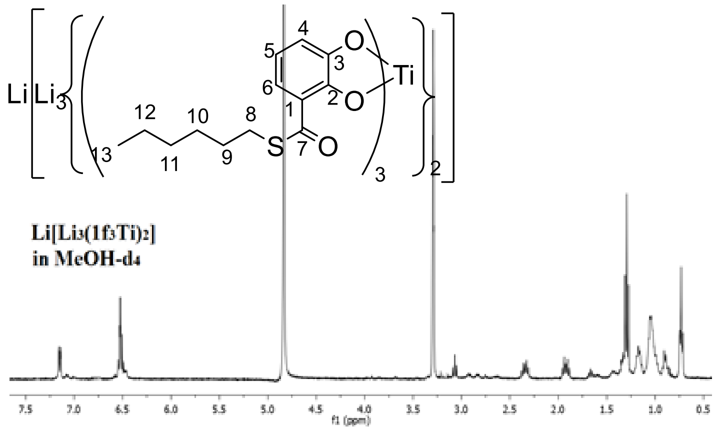

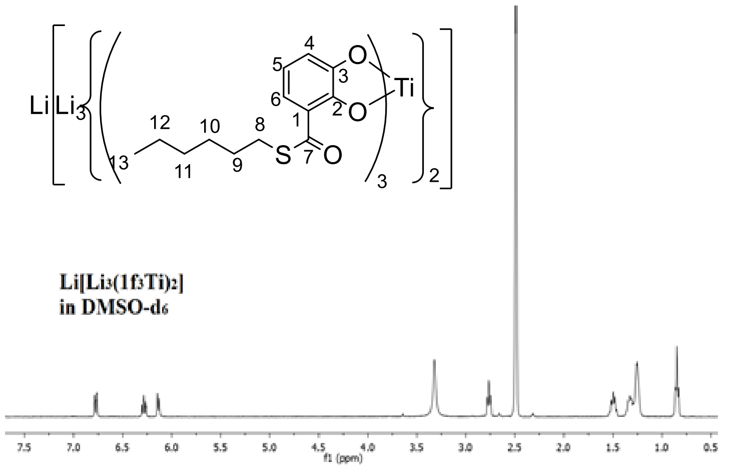

Li[Li$_3$(1g$_3$Ti)$_2$]:

Ligand **1g**-H_2 (50 mg, 0.19 mmol) is converted into the corresponding complex in methanol (50mL). The product is obtained after removal of the solvent under reduced pressure as red solid (quantitative). **^{1}H NMR** (400 MHz, MeOH-d$_4$): Dimer (major component): δ = 7.15 (dd, J = 7.9, 1.5 Hz, 1H, H$_{arom}$), 2.35-2.33 (m, 1H, SCH$_2$), 1.89-1.87 (m, 1H, SCH$_2$), 1.20-1.12 (m, 2H, CH$_2$CH$_2$) ppm. Monomer (minor component): δ = 7.07 (dd, J = 7.9, 1.5 Hz, 1H, H$_{arom}$), 2.94 (t, J =7.4 Hz, 2H, SCH$_2$), 1.66-1.64 (m, 2H, CH$_2$CH$_2$) ppm. Signals not listed are overlapping and cannot be assigned. **^{1}H NMR** (400 MHz, DMSO-d$_6$): Only monomer: δ = 6.78 (dd, J = 8.0, 1.5 Hz, 1H, H$_{arom}$), 6.28 (t, J = 8.0 Hz, 1H, H$_{arom}$), 6.14 (dd, J = 8.0, 1.5 Hz, 1H, H$_{arom}$), 2.76 (t, J = 7.3 Hz, 2H, SCH$_2$), 1.50-1.48 (m, 2H, CH$_2$CH$_2$), 1.32-1.21 (m, 8H, 4×CH$_2$), 0.85 (t, J = 6.8 Hz, 3H, CH$_2$CH$_3$) ppm. **MS** (negative ESI-MS, MeOH): m/z (%) = 1713.5349 (100, [M$_D$-Li$^+$], $C_{84}H_{108}O_{18}S_6Li_3Ti_2^-$, calcd. 1713.5300), 853.2589 (20, [M$_M$-Li$^+$], $C_{42}H_{54}O_9S_3LiTi^-$, calcd. 853.2570); 1727.5645 (30, [M$_D$+Li$^+$], $C_{84}H_{108}O_{18}S_6Li_5Ti_2^+$, calcd. 1727.5620),

867.2892 (30, $[M_M+Li^+]$, $C_{42}H_{54}O_9S_3Li_3Ti^+$, calcd. 867.2890). **IR (KBr)**: $\tilde{\nu}$ (cm^{-1}) = 3376, 3063, 2924, 2854, 2679, 2496, 2320, 2189, 2036, 1971, 1882, 1753, 1620, 1552, 1431, 1250, 1210, 1169, 1091, 1049, 863, 797, 727, 693. **Elemental analysis** $C_{84}H_{108}O_{18}S_6Li_4Ti_2 \cdot 2\ H_2O$: calcd. C = 57.40 %, H = 6.42 %; found C = 57.17 %, H = 6.59 %.

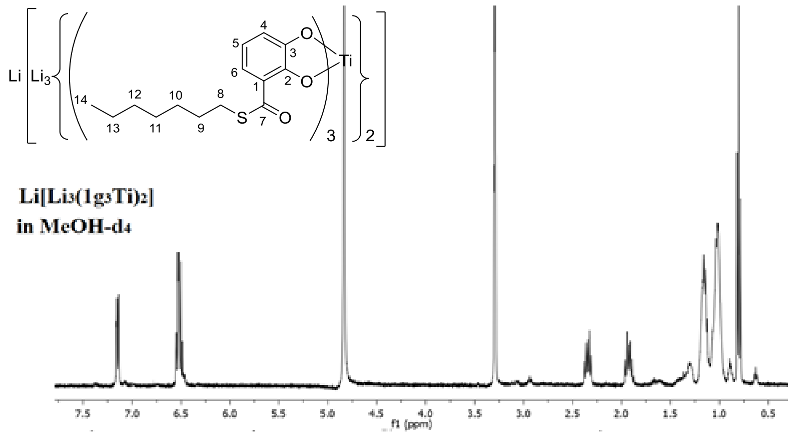

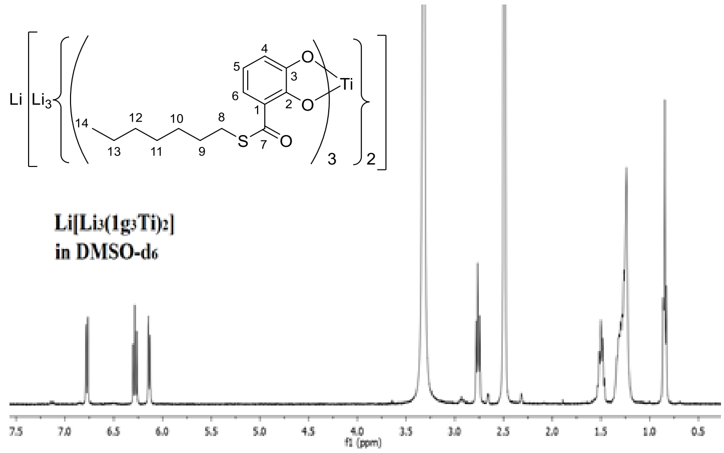

Li[Li$_3$(1h$_3$Ti)$_2$]:

Ligand **1h**-H_2 (50 mg, 0.18 mmol) is converted into the corresponding complex in methanol (50mL). The product is obtained after removal of the solvent under reduced pressure as red solid (quantitative). **^{1}H NMR** (400 MHz, MeOH-d$_4$): Dimer (major component): δ = 7.15 (dd, J = 7.8, 1.8 Hz, 1H, H_{arom}), 2.33-2.31 (m, 1H, SCH_2), 1.93-1.91 (m, 1H, SCH_2) ppm. Monomer (minor component): δ = 7.08 (dd, J = 8.0, 1.5 Hz, 1H, H_{arom}), 2.95 (t, J =7.3 Hz, 2H, SCH_2), 1.64-1.62 (m, 2H, CH_2CH_2) ppm. Signals not listed are overlapping and cannot be assigned. **^{1}H NMR** (400 MHz, DMSO-d$_6$): Only monomer: δ = 6.77 (dd, J = 7.9, 1.5 Hz, 1H, H_{arom}), 6.28 (t, J = 7.9 Hz, 1H, H_{arom}), 6.14 (dd, J = 7.9, 1.5 Hz, 1H, H_{arom}), 2.77 (t, J = 7.3 Hz, 2H, SCH_2), 1.47-1.45 (m, 2H, CH_2CH_2), 1.35-1.13 (m, 10H, $5{\times}CH_2$),

0.85 (t, J = 6.8 Hz, 3H, CH_2CH_3) ppm. **MS** (negative and positive ESI-MS, MeOH): m/z (%) = 1798.6229 (96, $[M_D\text{-}Li^+]$, $C_{90}H_{120}O_{18}S_6Li_3Ti_2^-$, calcd. 1797.6238), 895.3019 (100, $[M_M\text{-}Li^+]$, $C_{45}H_{60}O_9S_3LiTi^-$, calcd. 895.3039); 1812.6658 (20, $[M_D\text{+}Li^+]$, $C_{90}H_{120}O_{18}S_6Li_5Ti_2^+$, calcd. 1811.6558), 909.3400 (10, $[M_M\text{+}Li^+]$, $C_{45}H_{60}O_9S_3Li_3Ti^+$, calcd. 909.3359). **IR (KBr)**: $\tilde{\nu}$ (cm^{-1}) = 3380, 3062, 2922, 2855, 2670, 2499, 2316, 2192, 2111, 2026, 1899, 1741, 1619, 1548, 1432, 1348, 1248, 1210, 1094, 1045, 948, 863, 797, 692. **Elemental analysis** $C_{90}H_{120}O_{18}S_6Li_4Ti_2 \cdot 5\ H_2O$: calcd. C = 57.02 %, H = 6.91 %; found C = 57.04 %, H = 6.79 %.

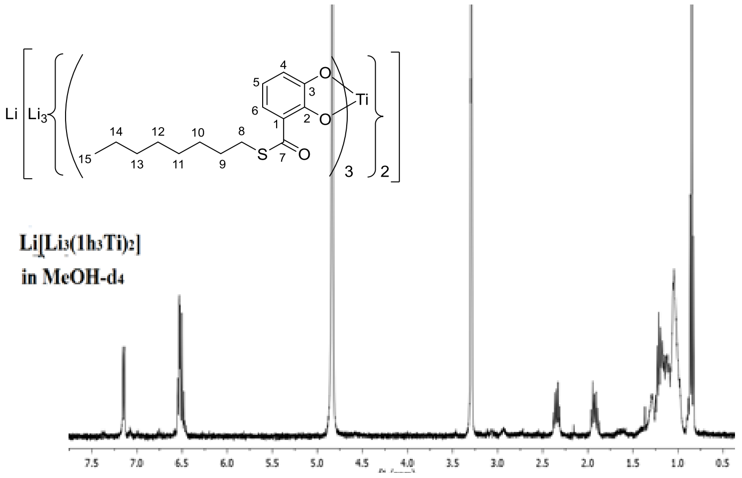

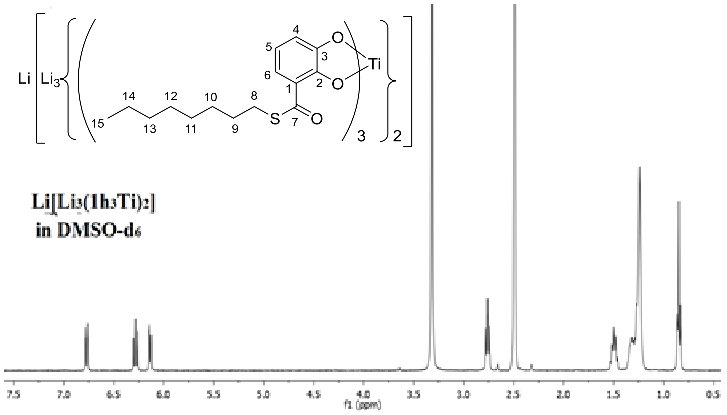

Li[Li$_3$(1i$_3$Ti)$_2$]:

Ligand **1i**-H_2 (50 mg, 0.17 mmol) is converted into the corresponding complex in methanol (50mL). The product is obtained after removal of the solvent under reduced pressure as red solid (quantitative). **^{1}H NMR** (400 MHz, MeOH-d_4): Dimer (major component): δ = 7.15 (dd, J = 8.0, 1.5 Hz, 1H, H_{arom}), 2.35-2.33 (m, 1H, SCH_2), 1.94-1.92 (m, 1H, SCH_2) ppm. Monomer (minor component): δ = 7.08 (dd, J = 8.0, 1.5 Hz, 1H, H_{arom}), 2.93 (t, J =7.3 Hz, 2H, SCH_2), 1.62-1.60 (m, 2H, CH_2CH_2) ppm. Signals not listed are overlapping and cannot be assigned. **^{1}H NMR** (400 MHz, DMSO-d_6): Only

monomer: δ = 6.77 (dd, J = 7.8, 1.4 Hz, 1H, H_{arom}), 6.28 (t, J = 7.8 Hz, 1H, H_{arom}), 6.14 (dd, J = 7.8, 1.4 Hz, 1H, H_{arom}), 2.76 (t, J = 7.3 Hz, 2H, SCH_2), 1.47-1.45 (m, 2H, CH_2CH_2), 1.35-1.15 (m, 12H, 6×CH_2), 0.84 (t, J = 6.8 Hz, 3H, CH_2CH_3) ppm. **MS** (negative and positive ESI-MS, MeOH): *m/z* (%) = 1882.7238 (70, [M_D-Li^+], $C_{96}H_{132}O_{18}S_6Li_3Ti_2^-$, calcd. 1882.9320); 1896.7512 (60, [M_D+Li^+], $C_{96}H_{132}O_{18}S_6Li_5Ti_2^+$, calcd. 1896.9640), 951.3826 (20, [M_M+Li^+], $C_{48}H_{66}O_9S_3Li_3Ti^+$, calcd. 951.3829). **IR (KBr)**: $\tilde{\nu}$ (cm^{-1}) = 3972, 3063, 2922, 2853, 2678, 2321, 2165, 2110, 1884, 1621, 1552, 1432, 1359, 1251, 1211, 1169, 1091, 1049, 945, 863, 797, 726, 693. **Elemental analysis** $C_{96}H_{132}O_{18}S_6Li_4Ti_2 \cdot 4\ H_2O$: calcd. C = 58.77 %, H = 7.19 %; found C = 58.75 %, H = 7.33 %.

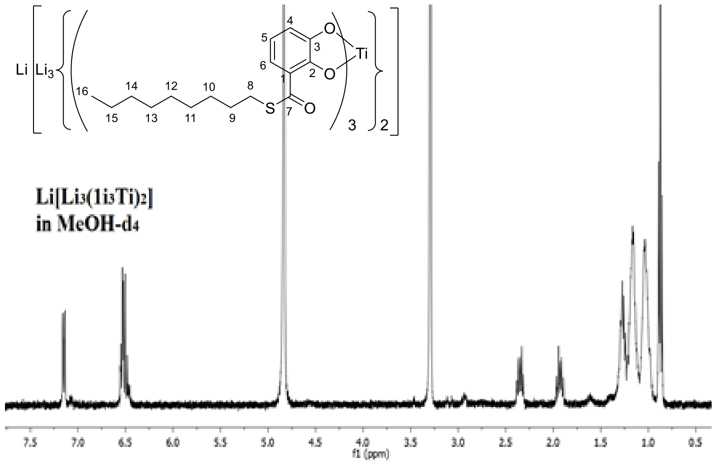

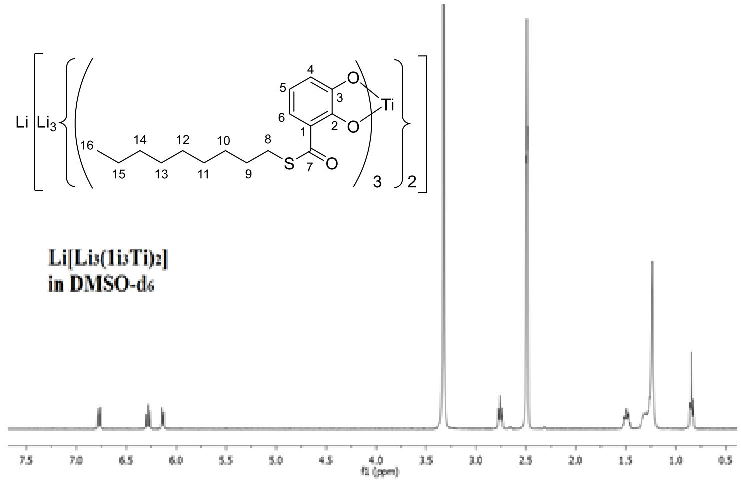

Li[Li_3($1j_3$Ti)$_2$]:

Ligand **1j**-H_2 (50 mg, 0.15 mmol) is converted into the corresponding complex in methanol (50mL). The product is obtained after removal of the solvent under reduced pressure as red solid (quantitative). **^{1}H NMR** (400 MHz, MeOH-d_4): Dimer (major component): δ = 7.15 (dd, J = 7.8, 1.6 Hz, 1H, H_{arom}), 2.35-2.33 (m, 1H, SCH_2), 1.93-1.91 (m, 1H, SCH_2) ppm. Monomer (minor component): δ =

7.07 (dd, J = 7.8, 1.5 Hz, 1H, H_{arom}), 2.94 (t, J =7.3 Hz, 2H, SCH_2), 1.62-1.60 (m, 2H, CH_2) ppm. Signals not listed are overlapping and cannot be assigned. **^{1}H NMR** (400 MHz, DMSO-d$_6$): Only monomer: δ = 6.77 (dd, J = 7.9, 1.5 Hz, 1H, H_{arom}), 6.28 (t, J = 7.9 Hz, 1H, H_{arom}), 6.13 (dd, J = 7.9, 1.5 Hz, 1H, H_{arom}), 2.73 (t, J = 7.3 Hz, 2H, SCH_2), 1.48 (m, 2H, CH_2), 1.34-1.20 (m, 18 H, 9×CH_2), 0.84 (t, J = 6.8 Hz, 3H, CH_3) ppm. **MS** (negative and positive ESI-MS, MeOH): m/z (%) = 2134.9997 (100, [M_D-Li^+], $C_{114}H_{168}O_{18}S_6Li_3Ti_2^-$, calcd. 2134.9994), 2149.0332 (10, [M_D+Li^+], $C_{114}H_{168}O_{18}S_6Li_5Ti_2^+$, calcd. 2149.0314), 1077.5240 (10, [M_M+Li^+], $C_{57}H_{84}O_9S_3Li_3Ti^+$, calcd. 1077.5237). **IR (KBr)**: $\tilde{\nu}$ (cm^{-1}) = 3369, 3062, 2922, 2852, 2679, 2167, 1621, 1552, 1518, 1433, 1360, 1252, 1213, 1170, 1091, 1050, 918, 863, 800, 726, 695. **Elemental analysis** $C_{114}H_{168}O_{18}S_6Li_4Ti_2$ · 2 H_2O: calcd. C = 62.85 %, H = 7.96 %; found C = 62.51 %, H = 7.75 %.

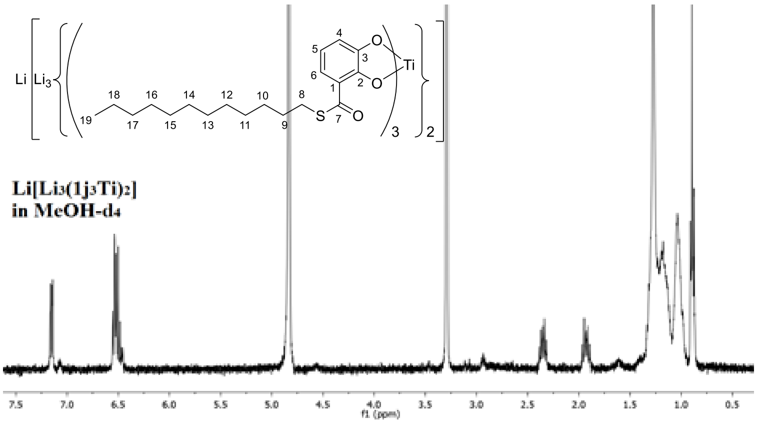

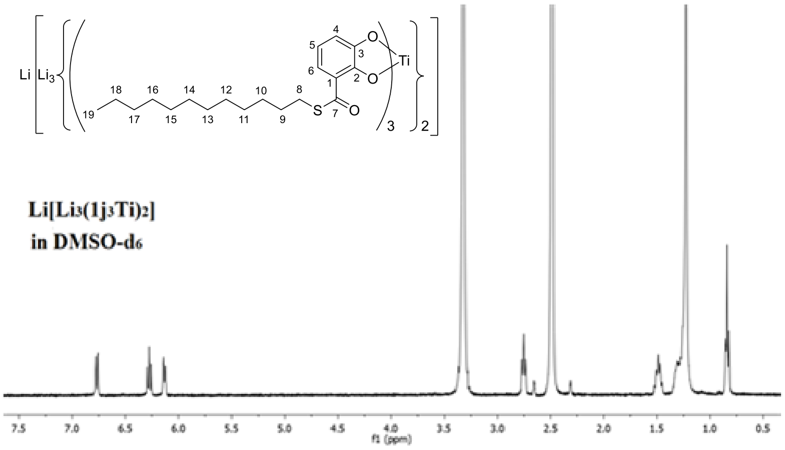

Li[Li$_3$(1k$_3$Ti)$_2$]:

Ligand **1k**-H_2 (50 mg, 0.24 mmol) is converted into the corresponding complex in methanol (50mL). The product is obtained after removal of the solvent under reduced pressure as red solid (quantitative). **^{1}H NMR** (400 MHz, MeOH-d$_4$): Dimer (major component): δ = 7.14 (dd, J = 7.9, 1.5 Hz,

1H, H_{arom}), 6.57-6.55 (m, 2H, H_{arom}), 2.79-2.77 (m, 1H, SCH), 0.99 (d, *J* = 7.2 Hz, 3H, CH_3), 0.90 (d, *J* = 6.5 Hz, 3H, CH_3) ppm. Monomer (minor component): δ = 7.05 (dd, *J* = 7.9, 1.6 Hz, 1H, H_{arom}), 6.47-6.45 (m, 2H, H_{arom}), 3.56-3.54 (m, 1H, SCH), 1.40 (d, *J* = 6.7 Hz, 3H, CH_3), 1.36 (d, *J* = 6.9 Hz, 3H, CH_3) ppm. **^{1}H NMR** (400 MHz, DMSO-d$_6$): Only monomer: δ = 6.76 (dd, *J* = 7.9, 1.5 Hz, 1H, H_{arom}), 6.28 (t, *J* = 7.9 Hz, 1H, H_{arom}), 6.13 (dd, *J* = 7.9, 1.5 Hz, 1H, H_{arom}), 3.57-3.55 (m, 1H, SCH), 1.26 (d, *J* = 6.9 Hz, 6H, 2×CH_3) ppm. **MS** (negative and positive ESI-MS, MeOH): *m/z* (%) = 1377.1578 (98, [M_D-Li^+], $C_{60}H_{60}O_{18}S_6Li_3Ti_2^-$, calcd. 1377.1542), 685.0666 (100, [M_M-Li^+], $C_{30}H_{30}O_9S_3LiTi^-$, calcd. 685.0691); 1391.1895 (100, [M_D+Li^+], $C_{60}H_{60}O_{18}S_6Li_5Ti_2^+$, calcd. 1391.1862), 699.1014 (20, [M_M+Li^+], $C_{30}H_{30}O_9S_3Li_3Ti^+$, calcd. 699.1011). **IR (KBr)**: $\tilde{\nu}$ (cm^{-1}) = 3630, 3393, 3183, 3061, 2966, 2926, 2863, 2678, 2314, 2173, 2047, 1983, 1920, 1742, 1617, 1552, 1430, 1359, 1250, 1209, 1165, 1091, 1046, 862, 796, 728, 693. **Elemental analysis** $C_{60}H_{60}O_{18}S_6Li_4Ti_2 \cdot 4\ H_2O$: calcd. C = 49.46 %, H = 4.70 %; found C = 49.26, H = 4.74 %.

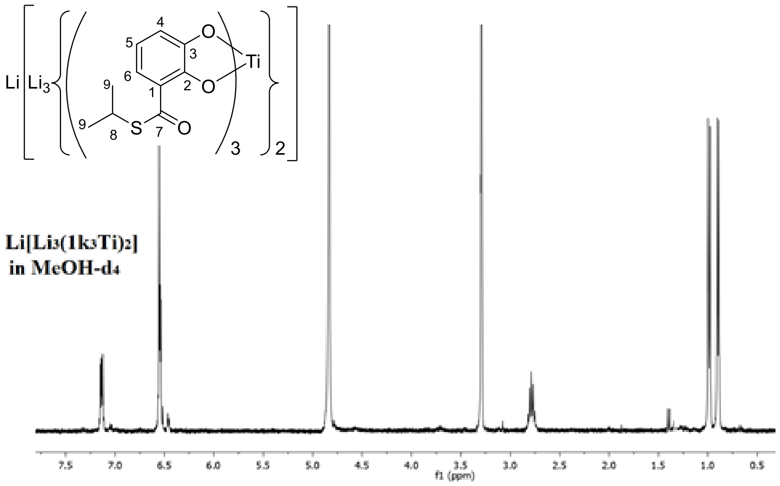

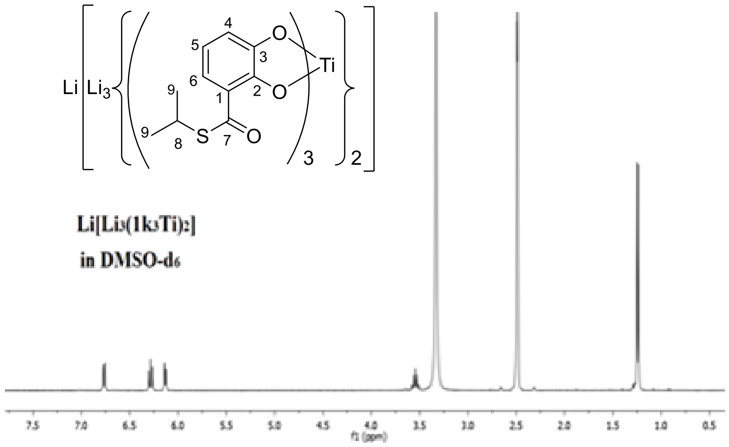

Li[Li$_3$(1l$_3$Ti)$_2$]:

Ligand **1l**-H$_2$ (50 mg, 0.24 mmol) is converted into the corresponding complex in methanol (50mL). The product is obtained after removal of the solvent under reduced pressure as red solid (quantitative). **^{1}H NMR** (400 MHz, MeOH-d$_4$): dimer (major component): δ = 7.19 (dd, *J* = 8.0, 1.6 Hz, 1H, H$_{arom}$), 2.33-2.31 (m, 1H, SCH$_2$), 1.84-1.82 (m, 1H, SCH$_2$), 1.38-1.36 (m, 1H, CH), 0.68 (d, *J* = 6.4 Hz, 3H, CH$_3$), 0.62(d, *J* = 6.4 Hz, 3H, CH$_3$) ppm. Monomer (minor component): δ = 7.10 (dd, *J* = 8.0, 1.6 Hz, 1H, H$_{arom}$), 6.47 (dd, *J* = 8.0, 1,6 Hz, 1H, H$_{arom}$), 2.87 (d, *J* = 6.9 Hz, 2H, SCH$_2$) ppm. **^{1}H NMR** (400 MHz, DMSO-d$_6$): Only monomer: δ = 6.78 (dd, *J* = 8.2, 1.4 Hz, 1H, H$_{arom}$), 6.28 (t, *J* = 8.2 Hz, 1H, H$_{arom}$), 6.14 (dd, *J* = 8.2, 1.4 Hz, 1H, H$_{arom}$), 2.77 (d, *J* = 6.7 Hz, 2H, SCH$_2$), 1.75-1.73 (m, 1H, CH), 0.93 (d, *J* = 6.7 Hz, 6H, 2×CH$_3$) ppm. **MS** (negative and positive ESI-MS, MeOH): *m/z* (%) = 1461.2508 (100, [M$_D$-Li$^+$], $C_{66}H_{72}O_{18}S_6Li_3Ti_2^-$, calcd. 1461.2482), 727.1163 (20, [M$_M$-Li$^+$], $C_{33}H_{36}O_9S_3LiTi^-$, calcd. 727.1161); 1475.2833 (50, [M$_D$+Li$^+$], $C_{66}H_{72}O_{18}S_6Li_5Ti_2^+$, calcd. 1475.2802), 741.1485 (30, [M$_M$+Li$^+$], $C_{33}H_{36}O_9S_3Li_3Ti^+$, calcd. 741.1481). **IR (KBr)**:$\tilde{\nu}$ (cm^{-1}) = 3642, 3396, 3061, 2957, 2869, 2658, 2501, 2322, 2167, 2111, 1990, 1952, 1906, 1623, 1552, 1432, 1365, 1252,1211, 1169, 1089, 1047, 945, 920, 863, 798, 729, 694. **Elemental analysis** $C_{66}H_{72}O_{18}S_6Li_4Ti_2 \cdot H_2O$: calcd. C = 53.30 %, H = 5.02 %; found C = 53.38 %, H = 5.19 %.

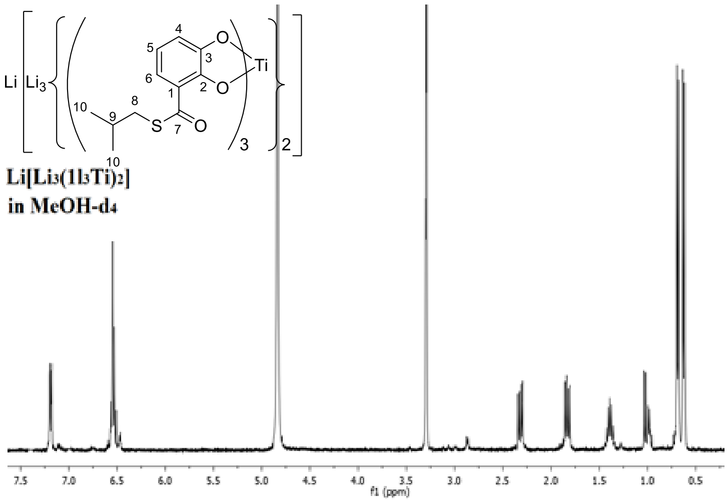

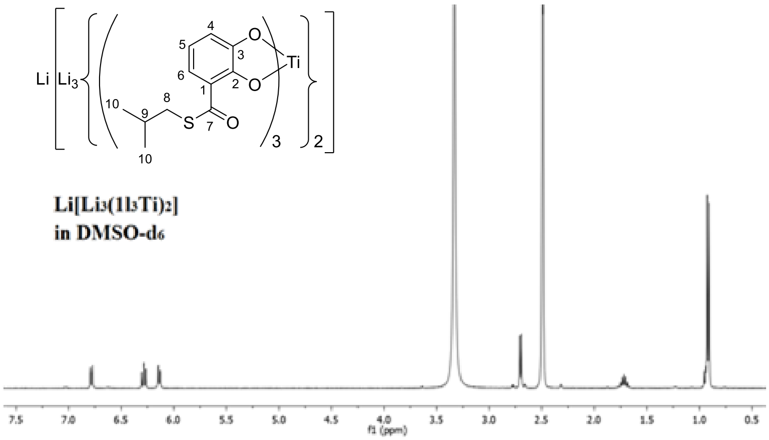

$Li[Li_3(1m_3Ti)_2]$:

Ligand **1m**-H_2 (50 mg, 0.21 mmol) is converted into the corresponding complex in methanol (50mL). The product is obtained after removal of the solvent under reduced pressure as red solid (quantitative). **^{1}H NMR** (400 MHz, MeOH-d_4): Dimer (major component): δ = 7.13 (dd, *J* = 7.9, 1.5 Hz, 1H, H_{arom}), 2.75-2.73 (m, 1H, SCH), 1.73-1.71 (m, 2H, $CH_{2cypent}$) ppm. Monomer (minor component): δ = 7.04 (dd, *J* = 7.9, 1.6 Hz, 1H, H_{arom}), 3.85-3.83 (m, 1H, SCH), 2.09-2.07 (m, 2H, $CH_{2cypent}$) ppm. Signals not listed are overlapping and cannot be assigned. **^{1}H NMR** (400 MHz, DMSO-d_6): Only monomer: δ = 6.75 (dd, *J* = 8.0, 1.4 Hz, 1H, H_{arom}), 6.28 (t, *J* = 8.0 Hz, 1H, H_{arom}), 6.13 (dd, *J* = 8.0, 1.4 Hz, 1H, H_{arom}), 3.63-3.61 (m, 1H, SCH), 2.03-2.01 (m, 2H, $CH_{2cypent}$), 1.65-1.50 (m, 6H, 3×$CH_{2cypent}$) ppm. **MS** (negative and positive ESI-MS, MeOH): *m/z* (%) = 763.1105 (100, [M_M-Li^+], $C_{36}H_{36}O_9S_3LiTi^-$, calcd. 763.1161); 1547.2811 (80, [M_D+Li^+], $C_{72}H_{72}O_{18}S_6Li_5Ti_2^+$, calcd. 1547.2802), 777.1471 (100, [M_M+Li^+], $C_{36}H_{36}O_9S_3Li_3Ti^+$, calcd. 777.1481). **IR (KBr)**:$\tilde{\nu}$ (cm^{-1}) = 3901, 3626, 3403, 3062, 2952, 2862, 2659, 2307, 2184, 2080, 19772, 1911, 1741, 1621, 1548, 1431, 1351, 1250, 1210, 1091, 1045, 940, 863, 797, 694. **Elemental analysis** $C_{72}H_{72}O_{18}S_6Li_4Ti_2 \cdot 4\ H_2O$: calcd. C = 53.61 %, H = 5.00 %; found C = 53.59 %, H = 4.96 %.

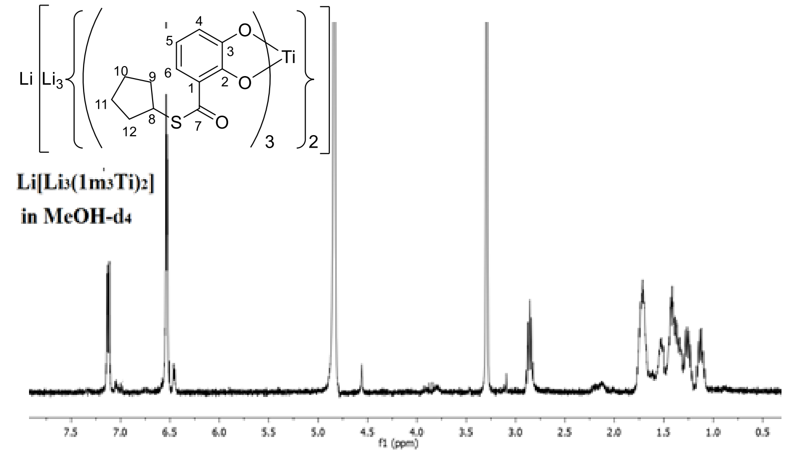

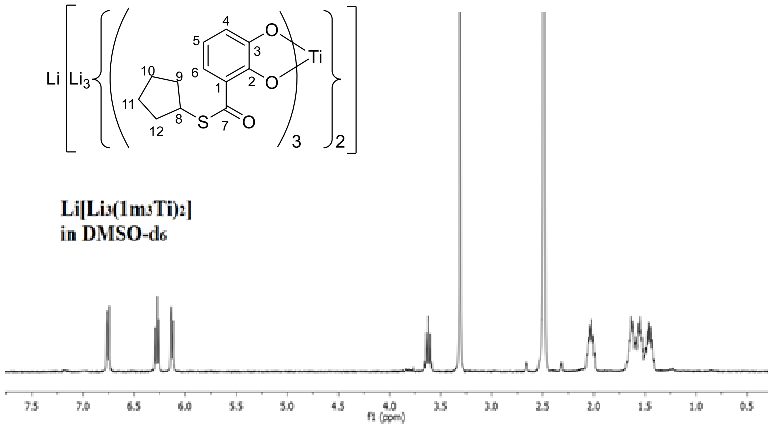

Li[Li$_3$(1n$_3$Ti)$_2$]:

Ligand **1n**-H$_2$ (50 mg, 0.20 mmol) is converted into the corresponding complex in methanol (50mL). The product is obtained after removal of the solvent under reduced pressure as red solid (quantitative). **^{1}H NMR** (400 MHz, MeOH-d$_4$): Dimer (major component): δ = 7.10 (dd, J = 7.9, 1.6 Hz, 1H, H$_{arom}$), 2.68-2.66 (m, 1H, SCH), 1.58-1.56 (m, 4H, 2×CH$_{2cyhex}$) ppm. Monomer (minor component): δ = 7.05 (dd, J = 7.9, 1.4 Hz, 1H, H$_{arom}$), 3.60-3.58 (m, 1H, SCH), 1.98-1.96 (m, 2H, CH$_{2cyhex}$), 1.79-1.77 (m, 2H, CH$_{2cyhex}$) ppm. Signals not listed are overlapping and cannot be assigned. **^{1}H NMR** (400 MHz, DMSO-d$_6$): Only monomer: δ = 6.76 (dd, J = 7.8, 1.5 Hz, 1H, H$_{arom}$), 6.28 (t, J = 7.8 Hz, 1H, H$_{arom}$), 6.13 (dd, J = 7.8, 1.5 Hz, 1H, H$_{arom}$), 3.43-3.41 (m, 1H, SCH), 1.90-1.88 (m, 2H, CH$_{2cyhex}$), 1.67-1.65 (m, 2H, CH$_{2cyhex}$), 1.58-1.15 (m, 6H, 3×CH$_{2cyhex}$) ppm. **MS** (negative and positive ESI-MS, MeOH): m/z (%) = 1617.3471 (100, [M$_D$-Li$^+$], $C_{78}H_{84}O_{18}S_6Li_3Ti_2^-$, calcd. 1617.3422), 805.1650 (20, [M$_M$-Li$^+$], $C_{39}H_{42}O_9S_3LiTi^-$, calcd. 805.1631); 1631.3758 (100, [M$_D$+Li$^+$], $C_{78}H_{84}O_{18}S_6Li_5Ti_2^+$, calcd. 1631.3742), 819.1952 (60, [M$_M$+Li$^+$], $C_{39}H_{42}O_9S_3Li_3Ti^+$, calcd. 819.1951). **IR (KBr)**: $\tilde{\nu}$ (cm^{-1}) = 3638, 3412, 3185, 3061, 2925, 2852, 2651, 2313, 2182, 2045, 1976,1889, 1741, 1619, 1431, 1346, 1249, 1210, 1091, 1045, 995, 864, 796,

691. **Elemental analysis** $C_{78}H_{84}O_{18}S_6Li_4Ti_2 \cdot 4\ H_2O$: calcd. C = 55.19 %, H = 5.46 %; found C = 55.46 %, H = 5.43 %.

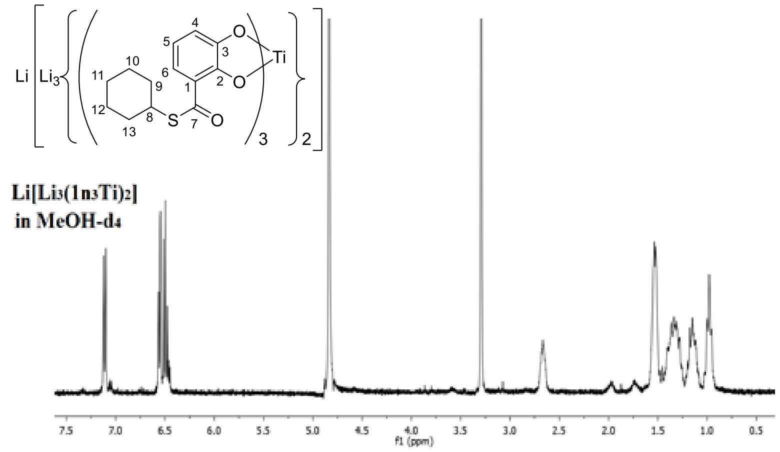

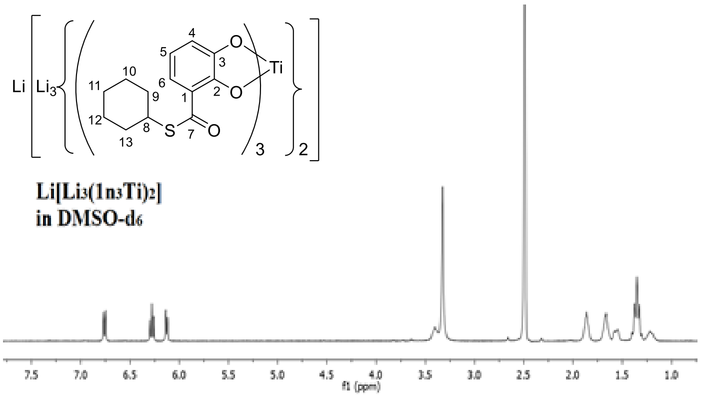

Li[Li$_3$(1o$_3$Ti)$_2$]:

Ligand **1o**-H$_2$ (100 mg, 0.41 mmol) is converted into the corresponding complex in methanol (50mL). The product is obtained after removal of the solvent under reduced pressure as a red solid (quantitative). **^{1}H NMR** (400 MHz, MeOH-d$_4$): Dimer (major component): δ = 7.50-7.45 (m, 2H, H$_{arom}$), 7.33-7.28 (m, 3H, H$_{arom}$), 7.25-7.22 (m, 1H, H$_{arom}$), 6.99 (dd, J = 7.8, 1.5 Hz, 1H, H$_{arom}$), 6.73 (t, J = 7.8 Hz, 1H, H$_{arom}$) ppm. Monomer (minor component): δ =7.40-7.37 (m, 2H, H$_{arom}$), 7.21-7.16 (m, 3H, H$_{arom}$), 7.10-7.04 (m, 1H, H$_{arom}$), 6.83 (t, J = 7.8 Hz, 1H, H$_{arom}$), 6.57-6.55 (m, 1H, H$_{arom}$) ppm. **MS** (negative and positive ESI-MS, MeOH): m/z (%) = 1581.0575 (80, [M$_D$-Li$^+$], $C_{78}H_{48}O_{18}S_6Li_3Ti_2^-$, calcd. 1581.0604), 787.0198 (100, [M$_M$-Li$^+$], $C_{39}H_{24}O_9S_3LiTi^-$, calcd. 787.0222); 1589.1939 (40, [M$_D$+H$^+$], $C_{78}H_{49}O_{18}S_6Li_4Ti_2^+$, calcd. 1589.0842), 801.0533 (100, [M$_M$+Li$^+$], $C_{39}H_{24}O_9S_3Li_3Ti^+$, calcd. 801.0542). **IR (KBr)**: $\tilde{\nu}$ (cm^{-1}) = 3635, 3359, 3059, 2927, 2849, 2655, 2501, 2305, 2110, 2026, 1984, 1889, 1747,

1640, 1586, 1546, 1433, 1344, 1302, 1248, 1206, 1025, 914, 858, 792, 735, 680. **Elemental analysis** $C_{78}H_{48}O_{18}S_6Li_4Ti_2 \cdot 8\ H_2O$: calcd. C = 54.05 %, H = 3.72 %; found C = 54.04 %, H = 3.87 %.

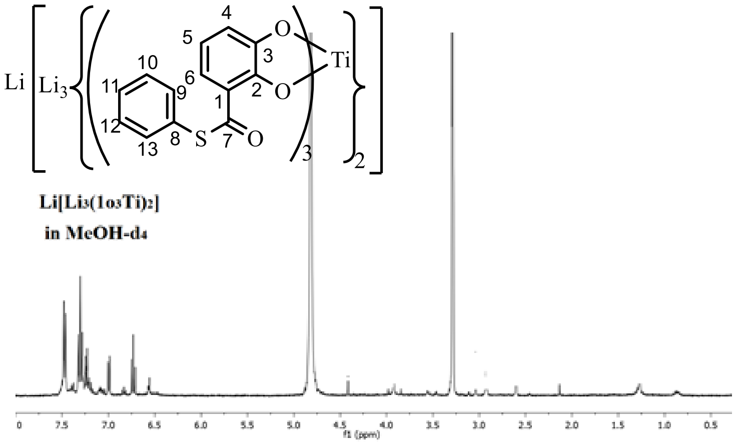

Li[Li$_3$(1p$_3$Ti)$_2$]:

Ligand **1p**-H_2 (50 mg, 0.19 mmol) is converted into the corresponding complex in methanol (50mL). The product is obtained after removal of the solvent under reduced pressure as red solid (quantitative). **^{1}H NMR** (400 MHz, MeOH-d$_4$): Dimer (major component): δ = 7.36-7.28 (m, 2H, H_{arom}), 3.40 (d, J = 13.8 Hz, 1H, H-8), 3.10 (d, J = 13.8 Hz, 1H, H-8) ppm. Monomer (minor component): δ = 7.21-7.17 (m, 2H, H_{arom}), 6.47 (dd, J = 7.9, 1.7 Hz, 1H, H_{arom}), 4.19 (s, 2H, H-8) ppm. Signals not listed are overlapping and cannot be assigned. **^{1}H NMR** (400 MHz, DMSO-d$_6$): Only monomer: δ = 7.28-7.15 (m, 5H, H_{arom}), 6.77 (dd, J = 7.8, 1.4 Hz, 1H, H_{arom}), 6.26 (t, J = 7.8 Hz, 1H, H_{arom}), 6.10 (dd, J = 7.8, 1.4 Hz, 1H, H_{arom}), 4.02 (s, 2H, SCH_2) ppm. **MS** (negative and positive ESI-MS, MeOH, acidified): m/z (%) = 1666.1683 (100, [M_D-Li^+], $C_{84}H_{60}O_{18}S_6Li_3Ti_2^-$, calcd. 1666.1542), 1679.1876 (10, [M_D+Li^+], $C_{84}H_{60}O_{18}S_6Li_5Ti_2^+$, calcd. 1679.1862). **IR (KBr)**: $\tilde{\nu}$ (cm^{-1}) = 3857, 3378, 3059, 3027, 2922, 2679, 2322, 2182, 2107, 2003, 1889, 1621, 1550, 1494, 1429, 1359, 1301, 1249, 1208, 1169, 1090, 1047, 916, 862, 793, 729, 689. **Elemental analysis** $C_{84}H_{60}O_{18}S_6Li_4Ti_2 \cdot 4\ H_2O$: calcd. C = 57.81 %, H = 3.93 %; found C = 57.91 %, H = 4.00 %.

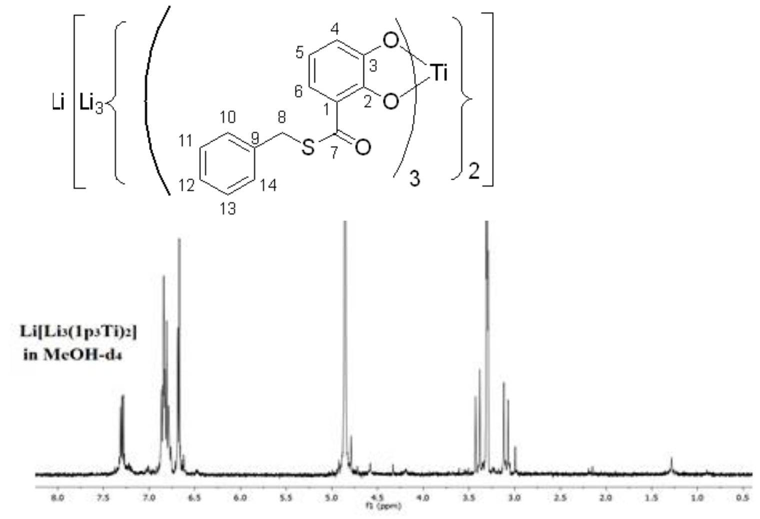

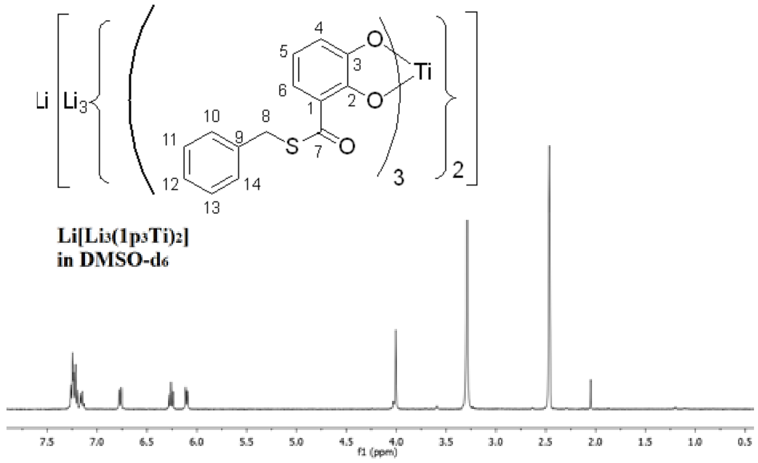

Ligand 2-H$_4$

Decane-1,10-diyl-bis(2,3-dihydroxybenzoate) (2-H$_4$):

The ligand is synthesized from 1,10-decanedithiol (0.24 ml, 1.10 mmol) by modification of the general procedure. Column chromatography (DCM, R_f = 0.20) results in the product as a white solid (62 %, 326 mg, 0.68 mmol). **M.p.**: 77 °C - 78 °C (last solvent: DCM). **^{1}H NMR** (600 MHz, $CDCl_3$): δ = 11.26 (s, 2H, 2xOH), 7.41 (dd, *J* = 7.8, 1.5 Hz, 2H, H_{arom}), 7.09 (dd, *J* = 7.8, 1.4 Hz, 2H, H_{arom}), 6.80 (t, *J* = 7.8 Hz, 2H, H_{arom}), 5.67 (s, 2H, 2xOH), 3.06 (t, *J* = 7.9 Hz, 4H), 1.70-1.65 (m, 4H), 1.45-1.40 (m, 4H), 1.36-1.28 (m, 8H) ppm. **MS** (negative and positive ESI-MS, MeOH, acidified): *m/z* (%) = 477.1390 (100, [M-H$^+$], $C_{24}H_{29}O_6S_2^-$, calcd. 477.1406); 501.1387 (100, [M+Na$^+$], $C_{24}H_{30}O_6S_2Na^+$, calcd. 501.1384). **IR** (KBr): $\tilde{\nu}$ (cm^{-1}) = 3474, 3096, 2923, 2852, 2324, 2160, 2109, 1931, 1695, 1629, 1577, 1449, 1334, 1265, 1212, 1168, 1080, 1032, 903, 855, 767, 728, 663. **Elemental Analysis** $C_{24}H_{30}O_6S_2$: calcd. C = 60.23 %, H = 6.32 %; found C = 60.24 %, H = 5.54 %.

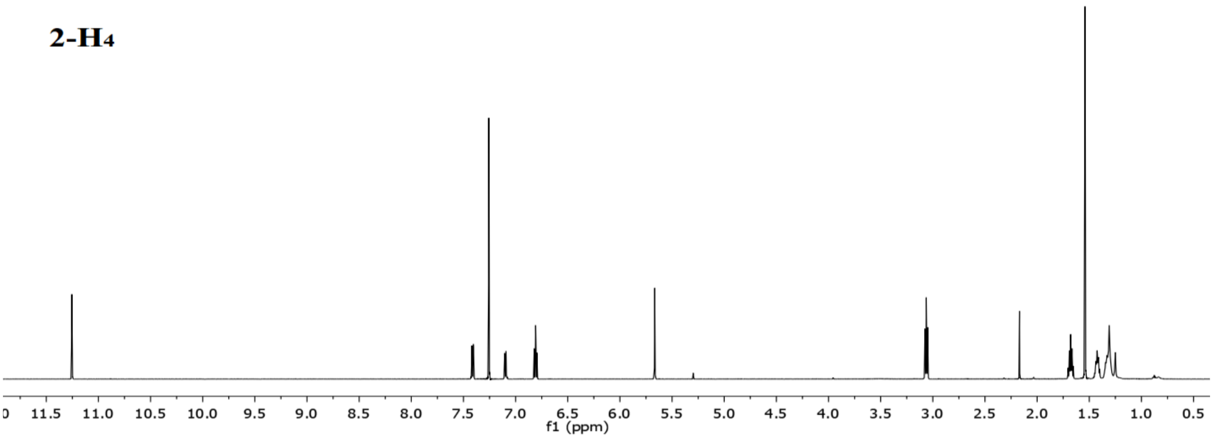

Complexes $M_4(2_3Ti_2)$

$Li[Li_3(2_3Ti_2)]$:

Ligand **2**-H_4 (50 mg, 0.10 mmol) is converted into the corresponding complex in methanol (50mL) by modification of the general procedure. The product is obtained after removal of the solvent under reduced pressure as red solid (quantitative). **^{1}H NMR** (400 MHz, MeOH-d_4): Compressed form: δ = 7.25 (dd, J = 7.6, 1.5 Hz, 2H, H_{arom}), 6.59-6.53 (m, 4H, H_{arom}), 2.60-2.57 (m, 2H), 2.00-1.92 (m, 2H), 1.90-1.83 (m, 4H), 1.37-0.91 (m, 12H) ppm. Expanded form: δ = 6.99-6.95 (m, 2H, $H_{arom.}$), 6.42 (dd, J = 7.8, 1.3 Hz, 2H, $H_{arom.}$), 6.35 (dd, J = 7.8, 1.3 Hz, 2H, $H_{arom.}$) ppm. Signals not listed are overlapping with LiCl and solvent and cannot be assigned. **^{1}H NMR** (600 MHz, DMSO-d_6): Compressed form: δ = 7.09 (dd, J = 7.6, 1.4 Hz, 2H, H_{arom}), 6.52 (t, J = 7.6 Hz, 2H, H_{arom}), 6.43 (dd, J = 7.6, 1.4 Hz, 2H, H_{arom}), 2.43-2.34 (m, 2H), 1.94-1.89 (m, 2H), 1.79-1.56 (m, 8H), 1.34-1.27 (m, 4H), 1.14-1.08 (m, 4H) ppm. Expanded form: δ = 6.76 (m, 2H, H_{arom}), 6.26 (m, 2H, H_{arom}), 6.10 (m, 2H, H_{arom}), 2.77-2.68 (m, 4H), 1.51-1.45 (m, 4H), 1.35-1.22 (m, 12H) ppm. **MS** (negative ESI-MS, MeOH): *m/z* (%) = 1539.2938 (100, $[M-Li^+]$, $C_{72}H_{78}O_{18}S_6Li_3Ti_2^-$, calcd. 1539.2952). **IR** (KBr): $\tilde{\upsilon}$ (cm^{-1}) = 3857, 3391, 2924, 2853, 2679, 2301, 2085, 1992, 1894, 1621, 1592, 1530, 1431, 1359, 1252, 1211, 1169, 1090, 1048, 933, 862, 797, 729, 692.

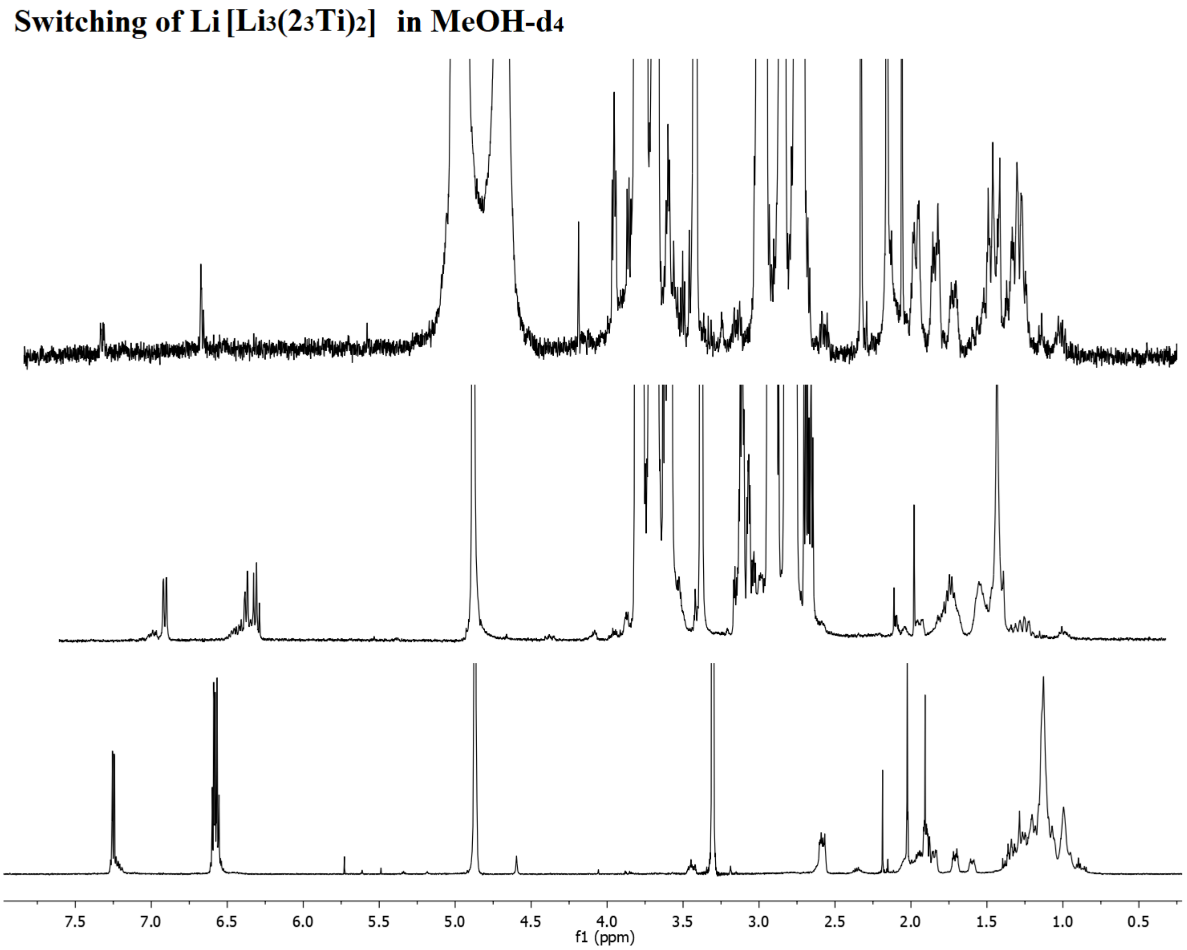

Figure. Reversible switching of the compressed $Li[Li_3(\mathbf{2})_3Ti_2]$ (bottom) in methanol-d_4 by successive addition of an excess of [2.1.1]cryptand (centre) and LiCl (top).

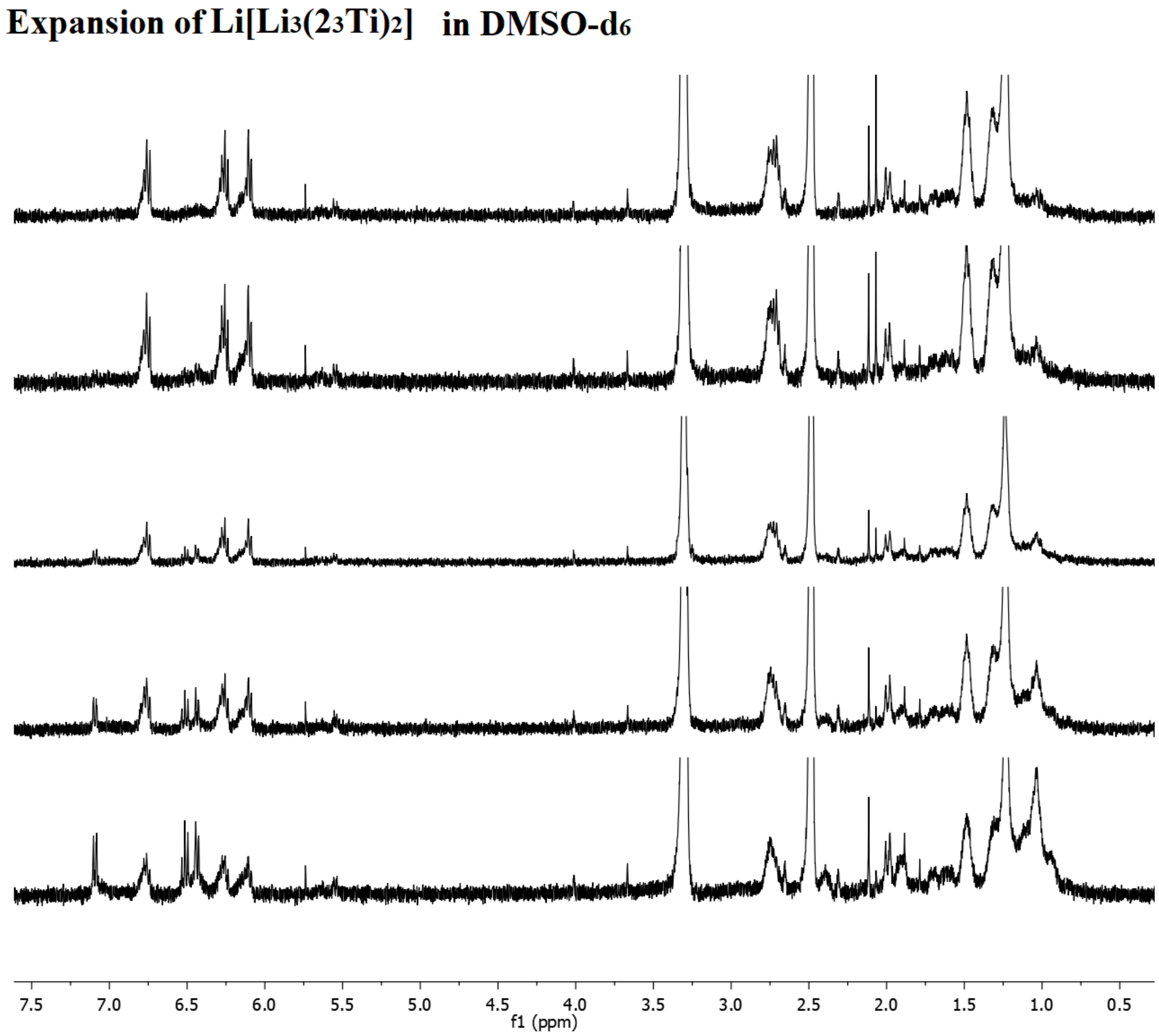

Figure Spontaneous expansion of Li[Li_3(**2**)$_3$$Ti_2$] upon dissolution in DMSO-d_6 (bottom to top).

K[K_3(2_3Ti_2)]:

Ligand **2**-H_4 (50 mg, 0.10 mmol) is converted into the corresponding complex in methanol (50mL) by modification of the general procedure. The product is obtained after removal of the solvent under reduced pressure as red solid (quantitative). **^{1}H NMR** (600 MHz, MeOH-d_4): δ = 6.97 (dd, J = 7.6, 1.5 Hz, 2H, H_{arom}), 6.43-6.34 (m, 4H, H_{arom}), 2.93-2.89 (m, 4H), 2.05-1.95 (m, 4H), 1.73-1.56 (m, 4H), 1.42-1.26 (m, 8H) ppm. **^{1}H NMR** (400 MHz, DMSO-d_6): δ = 6.88 (dd, J = 8.0, 1.5 Hz, 2H, H_{arom}), 6.34 (t, J = 8.0 Hz, 2H, H_{arom}), 6.23 (dd, J = 8.0, 1.5 Hz, 2H, H_{arom}), 2.87-2.76 (m, 4H), 2.66-2.60 (m, 4H),2.14-1.85 (m, 4H), 1.60-1.20 (m, 8H) ppm. **MS** (negative ESI-MS, MeOH): *m/z* (%) = 1636.1414 (100, [M-K^+], $C_{72}H_{78}O_{18}S_6K_3Ti_2^-$, calcd. 1636.7869). **IR** (KBr): $\tilde{\upsilon}$ (cm^{-1}) = 3331, 2925, 2853, 2652, 2323, 2213, 2173, 2104, 2021, 1993, 1947, 1880, 1586, 1524, 1437, 1371, 1263, 1208, 1168, 1083, 1043, 1016, 929, 863, 742, 666.

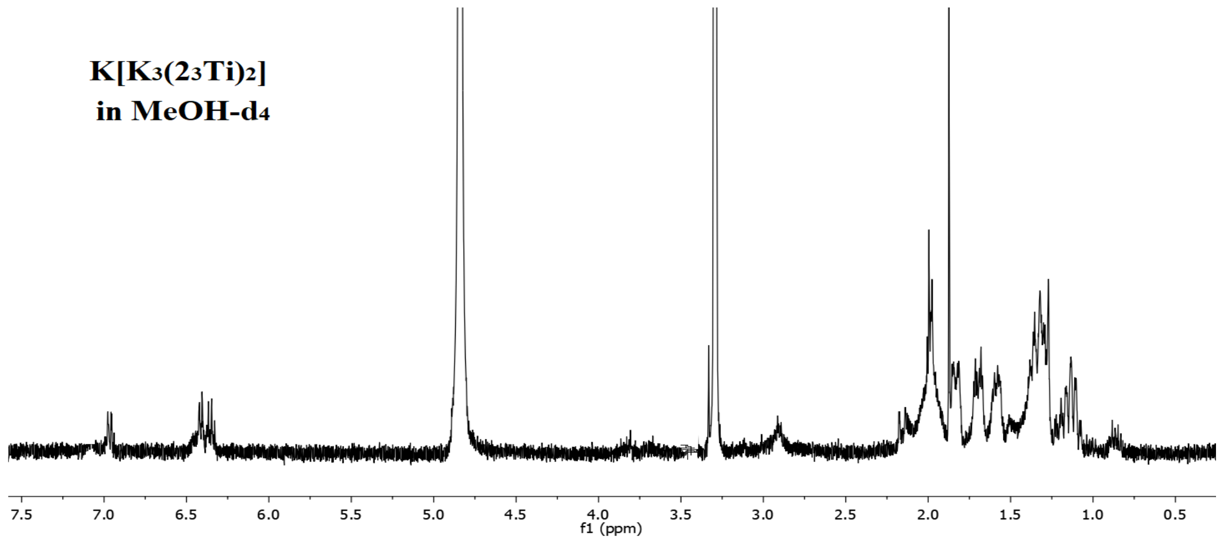
K[K3(23Ti)2]
in MeOH-d4
7.5
7.0
6.5
6.0
5.5
5.0
4.5
4.0
3.5
3.0
2.5
2.0
1.5
1.0
0.5
f1 (ppm)

3. Crystallographic studies

Single crystal x-ray diffraction data were collected by using ω-scans on a Stoe Stadivari diffractometer with a Eulerian 4 circle geometry, equipped with a CuK_{α} micro focus source (GeniX 3D HF Cu) and a Pilatus 200 K hybrid pixel detector (Dectris) at 100(2) K. Data collection and absorption correction were performed with the software package X-Area (STOE, X-Area 1.78, 2017), space group determination was performed with XPREP (1997). The structure was solved using a dual-space method with SHELXT (2018/2), structure refinement was done using SHELXL (2018/3) with a least squares procedure against F^2. Disordered solvent was removed by using Squeeze routine in the program PLATON. The solvent accessible volume amounts to 2003 Å^3 with 491 electrons.

CCDC 1965632 contains the supplementary crystallographic data for this structure.
